# Heterocycle compounds synthesized by amide ligand-promoted copper salt catalyzed construction of C–O(S) bonds†

**DOI:** 10.1039/d4ra00701h

**Published:** 2024-03-26

**Authors:** Ruiting Yin, Hailong Liu, Xue Yang, Xiaoyu Zhou, Xia Chen, Shenmin Li

**Affiliations:** a School of Environmental and Chemical Engineering, Dalian University Dalian 116622 Liaoning Province China; b School of Chemical and Materials Engineering, Liupanshui Normal College Guizhou Province 553004 China hailongliu@lpssy.edu.cn

## Abstract

We introduce a mild method for the ligand-promoted copper-catalyzed coupling of 2-halophenol to construct DBDO using cost-effective copper salts, ligands, and alkaline reagents. This method cleverly makes 2-bromophenol complete the Ullman reaction twice, achieves efficient C–O(S) bond coupling and intermolecular cyclization, and yields high amounts of oxygen(sulfur)-containing six-membered ring products. Less reactive 2-chlorophenol was also applied in this catalytic system. The application range of the copper-amide catalytic system was further expanded. Moreover, the success of a gram-scale reaction demonstrated that this operationally simple process is scalable.

## Abstract 2

We introduce a mild method for the ligand-promoted copper-catalyzed coupling of 2-halophenol to construct DBDO using cost-effective copper salts, ligands, and alkaline reagents.

## Graphical abstract

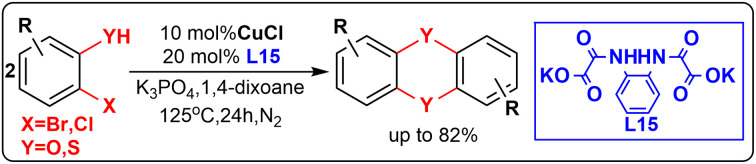

## Introduction

**Scheme 1:**
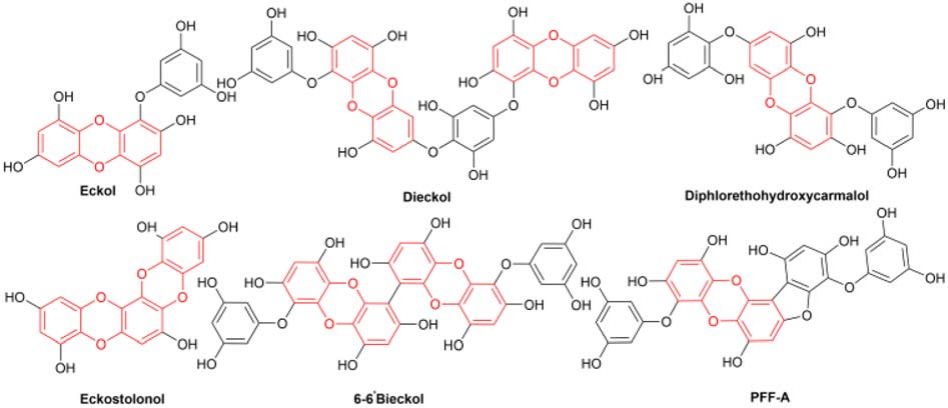
Selected drugs containing the core skeleton of dibenzo-*p*-dioxins.

The dibenzoxin group is an important fragment of a bioactive and effective drug molecule in seaweed extract.^1^ In recent years, it has been found that seaweed extract shows important biological activities, such as neuroprotection,^2^ hypoglycemic activity,^3^ antiviral properties (SARS-CoV-2, SARS-CoV-3CL^pro^)^4–6^ and the inhibition of the HIV-1 strain.^7^Thus, the dibenzoxin group has great potential in future drug research (Scheme 1).

**Scheme 2:**
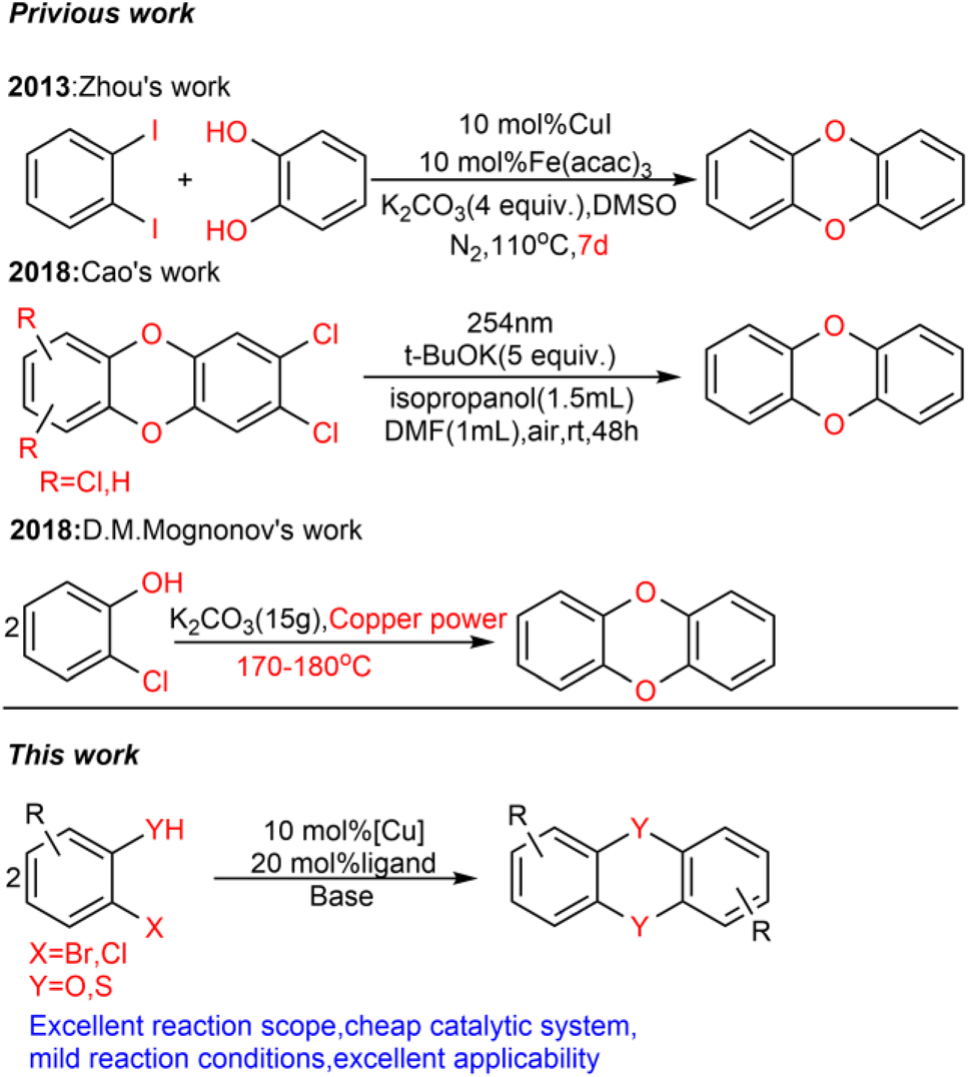
Previous synthetic methods for dibenzo-*p*-dioxins and the present catalytic system.

Accordingly, the development of synthetic methods for the construction of dibenzo-*p*-dioxins (DBDO) and their derivatives has been an area of intense research. At present, the synthesis protocols of DBDO are limited by low yields, harsh reaction conditions, and no substrate scope. In 1972, Albert E. Pohland^8^ and co-workers obtained a series of chlorinated DBDO, which was prepared for use as standards in the development of analytical methodology and for use in toxicological studies. In 2005, Catherine S. Evans^9^ and co-workers obtained DBDO with a low yield. In 2007, Jae-Yong Ryu^10^ and co-workers obtained DBDO in a 69% yield, but it needed to be carried out at a high temperature of 400 °C. In 2013, Zhou^11^ and co-workers used 1,2-diiodobenzene and diphenol as substrates and catalyzed by CuI/Fe(acac)_3_ under 110 °C and nitrogen atmosphere for 7 days to obtain six-membered ring compounds of DBDO with a yield of 12–18%. In 2018, Cao^12^ and co-workers reduced 2,3-dichlorodibenzo [*b*,*e*][1,4] dioxin and 2,3,7,8-tetrachlorodibenzo [*b*,*e*][1,4] dioxin (TCDD) by UV(254 nm) irradiation in a strong alkaline environment with yields of 81% and 83%, respectively. In 2018, D. M. Mognonov^13^ and co-workers found that in the presence of anhydrous potassium carbonate and ultrafine copper powder, *o*-chlorophenol was thermally condensed for 6–8 hours to obtain DBDO at 170–180 °C and recrystallized from benzene with a yield of 45% (Scheme 2).

Transition metal-catalyzed C–O bond coupling of aryl halides with alcohols to synthesize aryl ethers has become a very common method. However, the high cost of palladium and phosphine ligands hinder their development. MacMillan^14^ and co-workers used a more expensive ruthenium catalyst to complete their research, and Stradiotto^15^ and co-workers used nickel instead of palladium but it required pairing with phosphine ligands. The copper(i)-catalyzed Ullmann-type reaction developed by Ma and co-workers^16^ is one of the most effective and important synthetic strategies to construct C–C and C-heteroatom bonds, which is widely used in the synthesis of bioactive molecules, natural products and functional materials. However, to the best of our knowledge, the synthesis of DBDO and its derivatives catalyzed by copper salt has not yet been reported.

In order to fill this gap, we envisaged the use of appropriate ligands and reaction conditions to effectively synthesize DBDO. Therefore, we systematically studied many oxalate diamide ligands that showed excellent activity in promoting the arylation of other nucleophilic reagents catalyzed by Cu^17^ and tried to use Cu salts as catalyst precursors with amides and amide salts as ligands to promote the C–O bond binding of halogenated phenols. It was found that with the help of amide salt ligands, Cu-catalyzed 2-bromophenols to form DBDO can be carried out under relatively mild reaction conditions. Here, we explored the suitable ligands and reaction conditions.

## Results and discussion

As shown in Table 1, we selected CuI-catalyzed coupling of 2-bromophenol as a model reaction to optimize the reaction conditions of amide ligands. It was found that more than ten examples gave much lower yields, but the catalytic efficiency of L15 was the best. Therefore, we subsequently used L15 for futher optimization.

**Table 1:** Coupling reaction of 1a catalyzed by copper with different ligands^a^^,^^b^

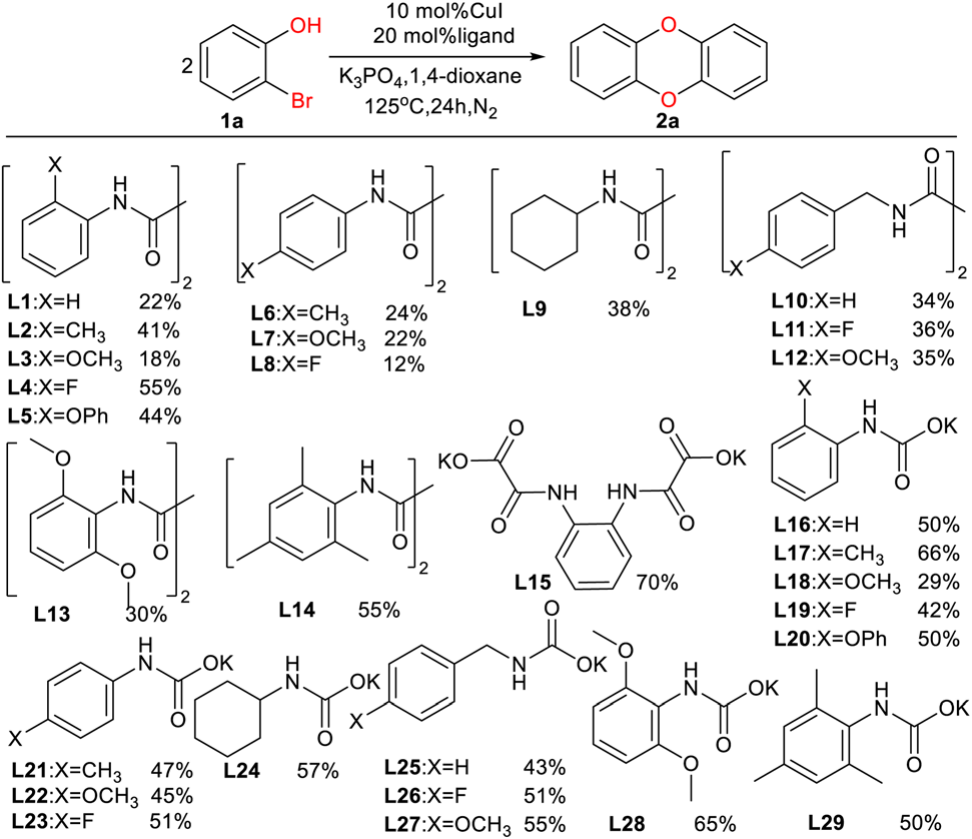

a The general conditions are as follows: 1a (2.0 mmol), CuI (0.1 mmol), ligand (0.2 mmol), base (3.0 mmol), 1,4-dioxane (3.0 mL).

b Determined by gas chromatography using *n*-decane as the internal standard.

As shown in Table 2, the anion of copper salts was found to be important for this reaction. Our copper salt screening investigations demonstrated that CuCl was suitable for our system, affording 2a in 85% yield (Table 2, entries 1, 5–9). K_3_PO_4_ can provide a suitable alkaline environment for the coupling of 2-bromophenol. After a series of solvents were tried, it was found that 1,4-dioxane showed a prominent advantage as a solvent (Table 2, entries 13–17) (see ESI† for detailed optimization procedures).

**Table 2:** Optimization of reaction conditions

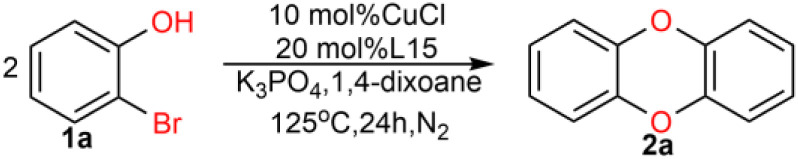		
Entry^a^	Deviation from standard conditions	Yield^b^ (%)
1	None	85(82)^c^
2	CuCl (20 mol%)	84
3	CuCl (5 mol%)	76
4	No CuCl	0
5	CuI instead of CuCl	70
6	Cu_2_O instead of CuCl	45
7	CuBr instead of CuCl	58
8	Cu(OAc)_2_ instead of CuCl	61
9	Cu(OTf)_2_ instead of CuCl	49
10	L15 (40 mol%)	79
11	No L15	5
12	No K_3_PO_4_	10
13	DMSO instead of 1,4-dioxane	13
14	DMF instead of 1,4-dioxane	10
15	Toluene instead of 1,4-dioxane	50
16	H_2_O instead of 1,4-dioxane	0
17	EtOH instead of 1,4-dioxane	38
18	90 °C instead of 125 °C	67
19	Air instead of N_2_	10

a The general conditions are as follows: 1a (2.0 mmol), CuCl (0.1 mmol), L15 (0.2 mmol), K_3_PO_4_ (3.0 mmol), 1,4-dioxane (3.0 mL).

b Determined by gas chromatography using *n*-decane as the internal standard.

c Isolated yield.

Under the optimized reaction conditions, we set out to investigate the scope of the reaction with respect to *o*-halo(thio)phenols (Table 3). 2-Bromophenol with methyl substituent could proceed smoothly and gave the products in good yields (Table 3, entries 3–6). Moreover, the steric hindrance affected the outcome of the reaction to a certain extent. When –CH_3_ was present *ortho* to the reactive site, the yields of the reaction decreased slightly (Table 3, entries 3 and 4 *vs.* entries 5 and 6). The electronic properties of the substituents have no obvious effect on the reaction results. Electron-rich and electron-deficient substrates all afforded the expected products in good yields (Table 3, entries 7–12). Unfortunately, nitro and cyano-substituted 2-bromophenols (1l and 1m) have poor performance in the coupling reaction, which might be due to the electron-withdrawing nature of the nitro and cyano substituents. To our delight, we tried to apply the above experimental scheme to the C–S bond coupling reaction of 2-bromothiophenol and received good feedback to obtain the target product thianthrene in 75% yield. It is proved that L15 is not only an effective ligand that can efficiently promote copper salt-catalyzed C–O bond coupling and complete cyclization but also has good potential in promoting C–S bond coupling reaction. Aryl chlorides remain an uncommon reagent in coupling reactions because of the low reactivity of the relatively inert C–Cl bond. However, because of their lower cost and wider diversity, aryl chlorides are always attractive substrates instead of their bromo counterparts. We were pleased to find that *o*-chlorophenol was also reactive under the established catalytic conditions, albeit with a low yield (Table 3, entry 2).

**Table 3:** Coupling reaction of 2-bromophenol (heteroaryl) catalyzed by CuCl

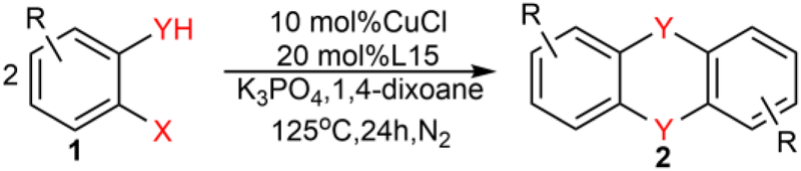			
Entry^a^	1	2	Yield^b^ (%)
1	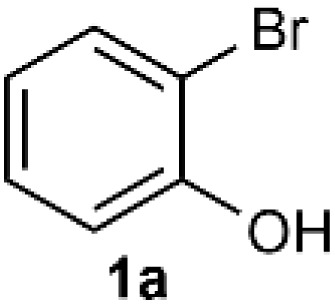	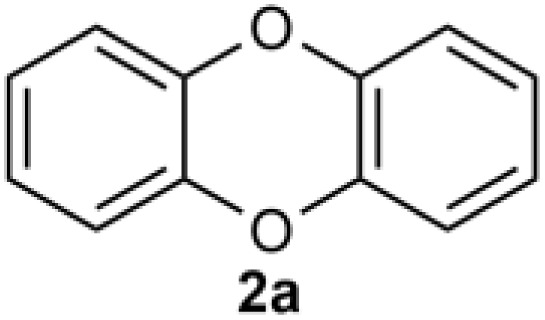	82
2	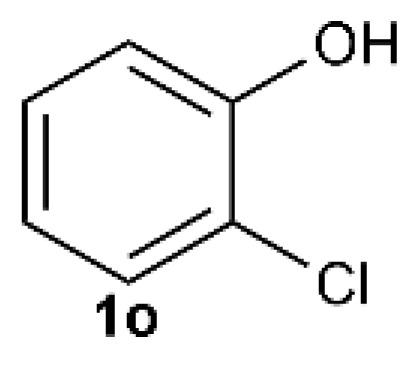		33
3	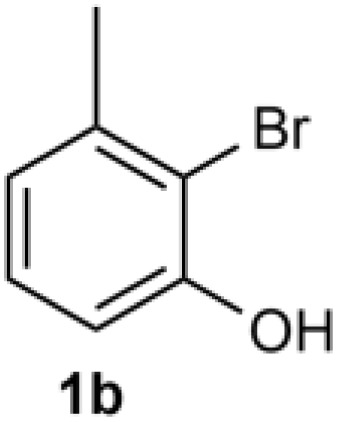	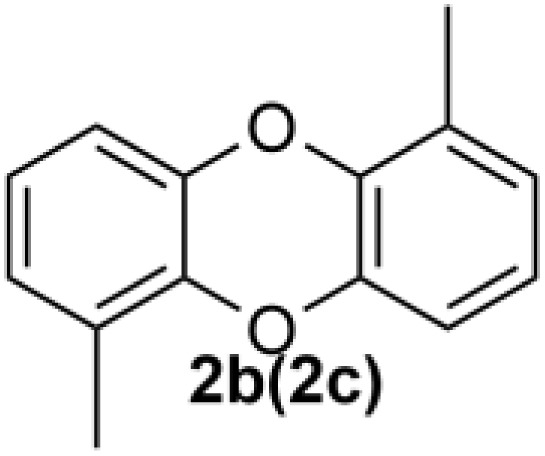	74
4	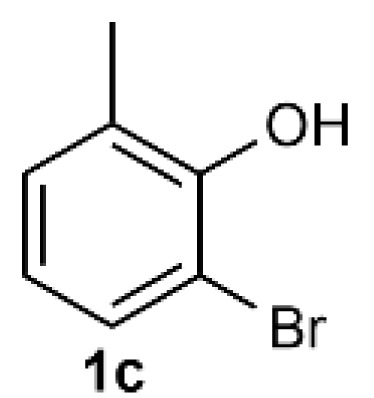		76
5	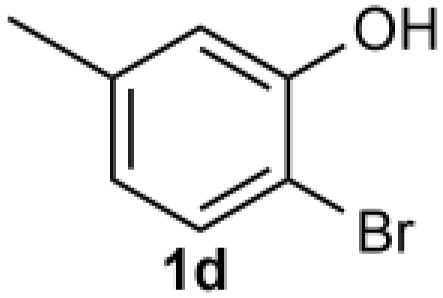	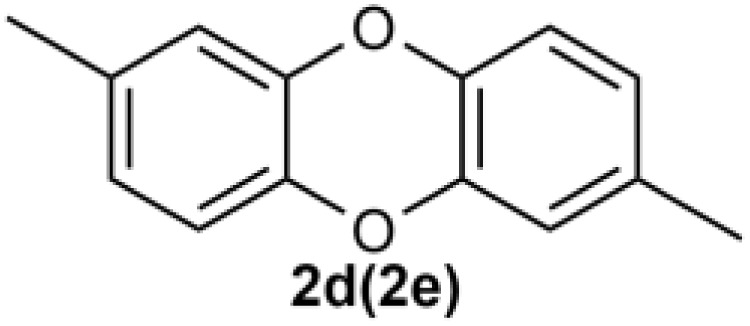	80
6	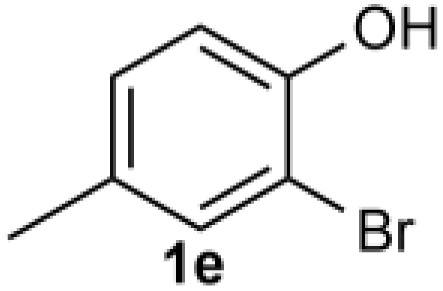		81
7	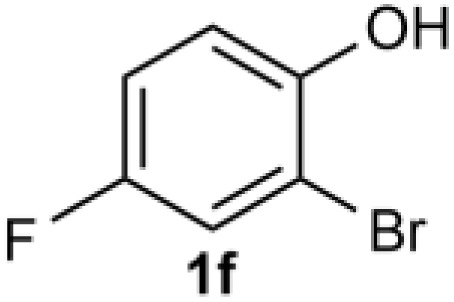	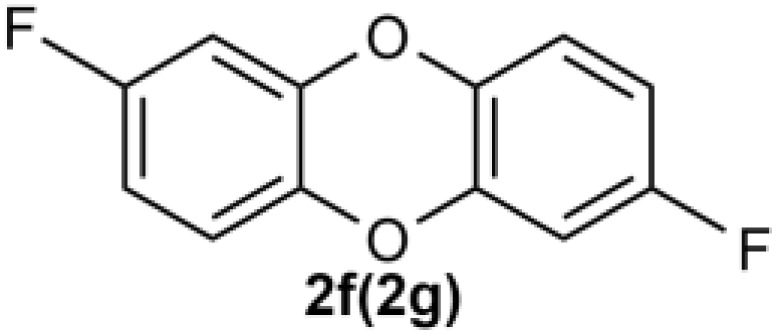	74
8	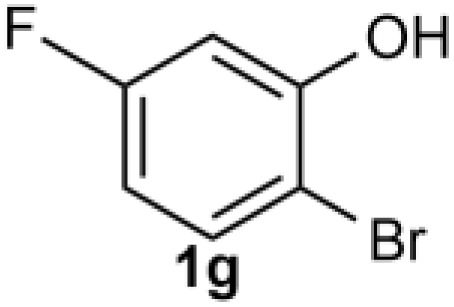		76
9	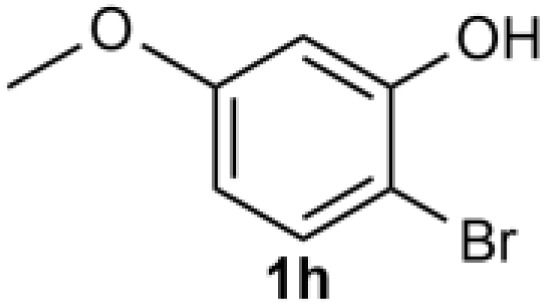	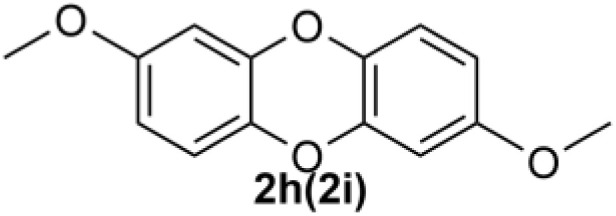	77
10	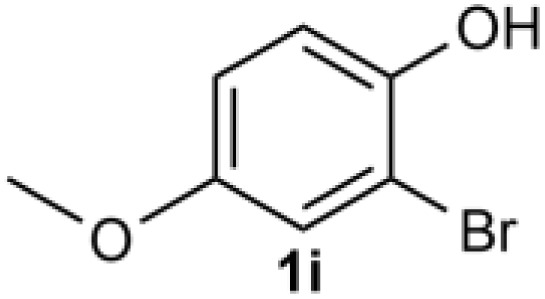		75
11	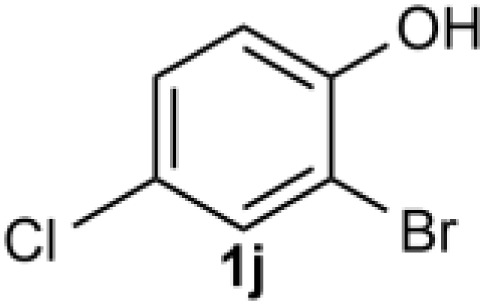	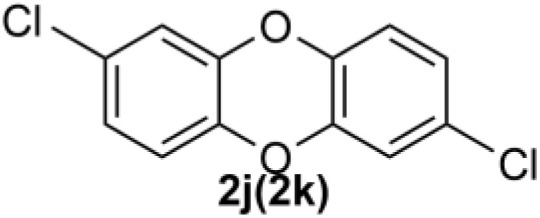	74
12	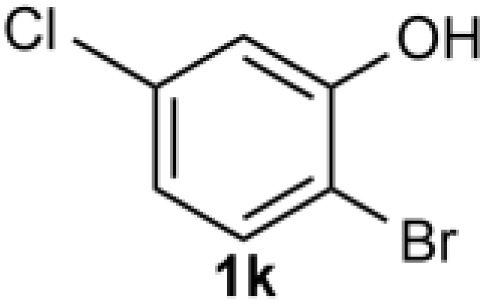		73
13	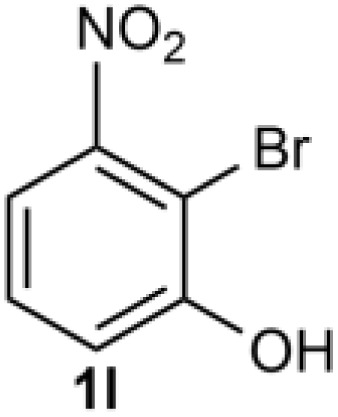	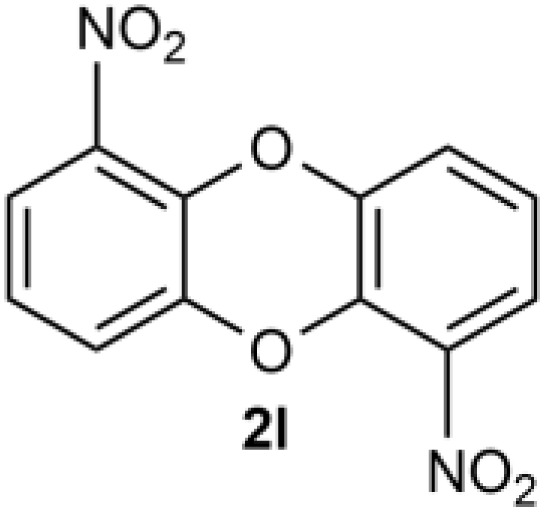	0
14	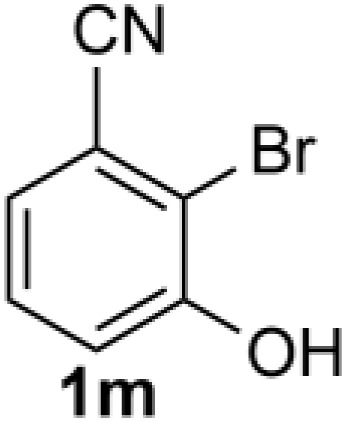	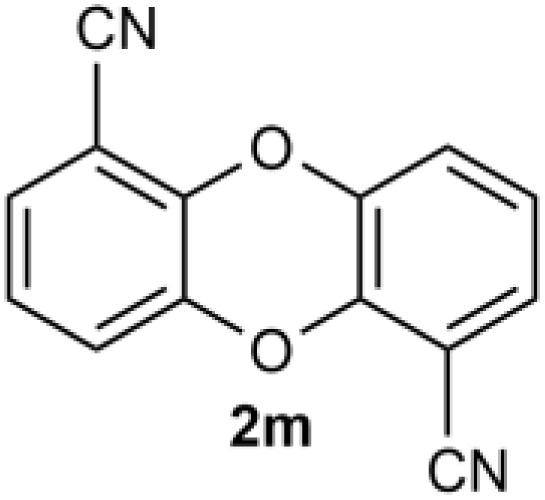	0
15	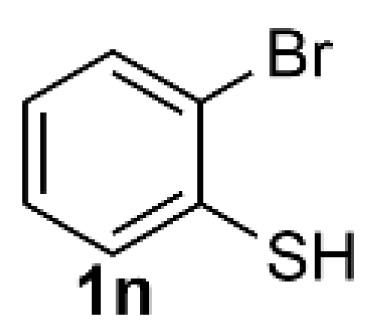	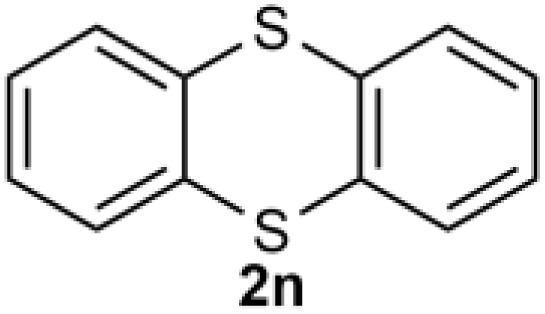	75

a The general conditions are as follows: 1 (2.0 mmol), CuCl (0.1 mmol), L15 (0.2 mmol), K_3_PO_4_ (3.0 mmol), 1,4-dioxane (3.0 mL).

b Isolated yield.

**Scheme 3:**
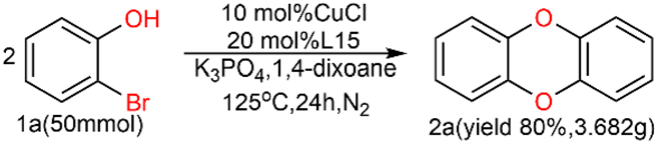
Scaled-up reaction for the formation of DBDO.

A scaled-up experiment (Scheme 3) was performed on a gram-scale reaction under the same conditions. Scaled up by 25 times to 50 mmol, the reaction proceeded as expected to give the desired product 2a in 80% isolated yield, demonstrating the practicality of this method.

**Scheme 4:**
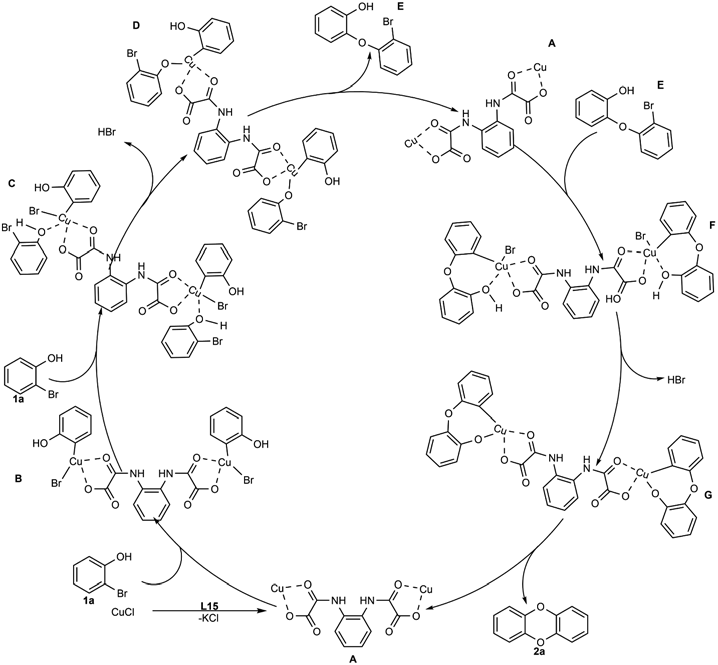
Possible mechanism for the formation of DBDO by CuCl/L15-catalyzed coupling of 2-bromophenol.

In this study, DBDO was formed by C–O bond binding of 2-bromophenol twice, so we propose that the coupling reaction might undergo a typical Ullmann reaction, as described in (Scheme 4) The catalytic cycle was completed under the catalysis of CuCl and L15.

## Conclusions

In summary, we have presented a convenient and mild method for ligand-promoted copper-catalyzed coupling of 2-halophenol to construct DBDO. We utilized cost-effective and readily available copper salts, ligands and alkaline reagents to achieve C–O(S) bond coupling and facilitate intermolecular cyclization. High yields of oxygen-containing six-membered ring products were obtained, effectively promoting the cross-coupling of C–S bonds and completing the construction of sulfur-containing six-membered rings. Furthermore, the less reactive 2-chlorophenol could also be employed in this catalytic system.

## Author contributions

Ruiting Yin performed the experiments and analysed the data. Ruiting Yin and Hailong Liu designed the study and supervised the project. Ruiting Yin wrote the manuscript. All authors had approved the final version.

## Conflicts of interest

There are no conflicts to declare.

## Supplementary Material

RA-014-D4RA00701H-s001
