# Supplementary material for: Heterocycle compounds synthesized by amide ligand-promoted copper salt catalyzed construction of C–O(S) bonds
Source: RSC Adv. 2024 Mar 26;14(14):10034–8. doi: 10.1039/d4ra00701h (PMC10964132; doi:10.1039/d4ra00701h)
Supplement: RA-014-D4RA00701H-s001 [file RA-014-D4RA00701H-s001.pdf]

Electronic Supplementary Material (ESI) for RSC Advances.
This journal is © The Royal Society of Chemistry 2024

Synthesis of heterocyclic compounds by C-O bond catalyzed by copper catalyzed by amide ligands

Supporting information

1. General information 1

2. General procedures for the preparation of ligands 2

3. General procedure for copper-catalyzed 2-bromophenol coupling 12

4. Copies of ^{1}H and ^{13}C spectra of ligands and (hetero) dibenzoxins 17

1. General information

Reagents: All commercial materials are used as-is unless otherwise stated. THF is distilled in Na for the preparation of ligands. 1, 4-Dioxane is distilled in Na for copper catalyzed reactions.

Reactions: All reactions of copper-catalyzed C-O coupling are carried out on an open workbench and in a nitrogen atmosphere in resealable Schlenk test tubes with teflon tees. Unless otherwise indicated, the solution of solvent and reagent/reactant is transferred to the reaction tube by microinjector or plastic syringe (equipped with metal needle) under positive nitrogen pressure.

Instruments: NMR spectra was recorded on JEOL ECS-400 nuclear magnetic resonance spectrometer and calibrated using residual solvent peaks as an internal reference, gas chromatography was recorded on SCION 456C, high resolution mass experiments were operated on a commercial instrument, melting point was recorded on INESA WRS-1B. Multiplicities are recorded as: br = broad, s = singlet, d = doublet, t = triplet, q = quartet, hept = heptet, dd = doublet of doublets, m = multiplet.

2. General procedures for the preparation of ligands

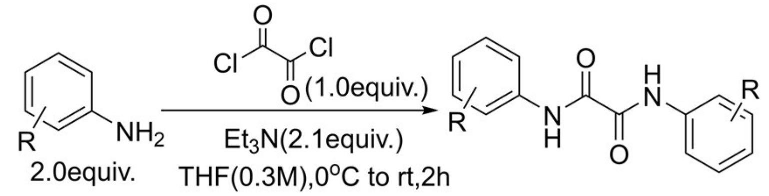

General procedure A: To a solution of the corresponding aniline (2.0 equiv.) in THF (0.3 M) was added Et_3N (2.1 equiv.). Oxaloyl chloride (1.0 equiv.) was then slowly dripped into the mixture under an ice bath. The resulting mixture was stirred at room temperature for 2 hours, then vacuum concentrated to remove the solvent, and water was added to the resulting residue to dissolve $Et_3N{\cdot}HCl$. The slurry is then filtered and the solids on the filter paper are washed with water and cold ether. These solids are recrystallized, dried in a vacuum, and the corresponding N,N '-diaryloxamide is obtained. They are pure enough to be used without further purification.

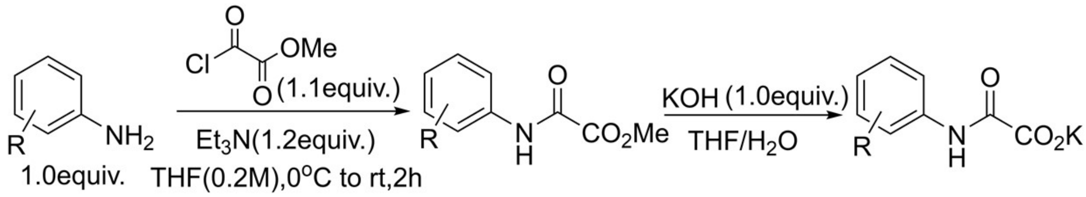

General procedure B: To a solution of the corresponding aniline (1.0 equiv.) in THF (0.2M) was added Et_3N (1.2 equiv.). Mono-methyl oxalyl chloride (1.1 equiv.) was then slowly added to the solution under an ice water bath. After stirring the resulting mixture at room temperature for 2 hours, wash the mixture with the same volume of water. The organic phase is dried with Na_2SO_4 and evaporated. The crude product was purified by silica gel chromatography to obtain a light yellow solid with a yield of 89%. The obtained yellow solid was dissolved in THF (0.5M), KOH (2.0M aqueous solution,1.0eq) was added to the mixed solution, and the obtained mixture was stirred at room temperature, and tested by TLC until it was completely consumed. Vacuum concentration removes THF and water, and oven drying produces a white solid at 98% yield.

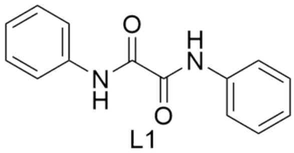

L1

The L1 was prepared as a white solid in 83% yield from aniline and oxalyl chloride following the general procedure A. M.P.: 253-254°C.^{1}H NMR (400 MHz, DMSO-d_6) δ 10.86 (s, 2H), 7.87 (d, J = 8.5 Hz, 4H), 7.38 (t, J = 7.9 Hz, 4H), 7.16 (t, J = 7.4 Hz, 2H). ^{13}C NMR (101 MHz, DMSO-d_6) δ 158.64, 137.70, 128.81, 124.67, 120.49; ESI-HRMS m/z calcd for $C_{14}H_{13}N_2O_2$ $(M + H)^+$ 241.0950, found: 241.0955.

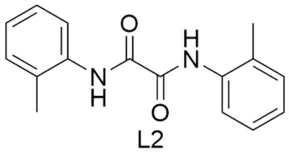

L2

The L2 was prepared as a white solid in 85% yield from 2-methylaniline and oxalyl chloride following the general procedure A. M.P.: 216-217°C. ^{1}H NMR (400 MHz, DMSO-d_6) δ 10.30 (s, 2H), 7.50 (d, J = 7.9 Hz, 2H), 7.32 – 7.13 (m, 6H), 2.25 (s, 6H). ^{13}C NMR (101 MHz, DMSO-d_6) δ 158.57, 135.09, 132.40, 130.47, 126.25, 124.97, 17.69; ESI-HRMS m/z calcd for $C_{16}H_{17}N_2O_2$ $(M + H)^+$ 269.0965, found: 269.0963.

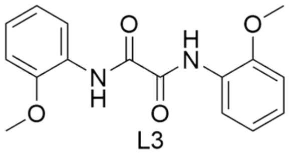

L3

The L3 was prepared as a white solid in 86% yield from 2-methoxyaniline and oxalyl chloride following the general procedure A. M.P.: 275-277°C. ^{1}H NMR (400 MHz, DMSO-d_6) δ 10.74 (s, 2H), 7.73 (d, *J* = 8.3 Hz, 4H), 7.17 (d, *J* = 8.3 Hz, 4H), 2.28 (s, 6H). ^{13}C NMR (101 MHz, DMSO-d_6) δ 158.52, 135.20, 133.73, 129.19, 120.40, 20.58; ESI-HRMS m/z calcd for $C_{16}H_{17}N_2O_4$ $(M + H)^+$ 301.0995, found: 301.0990.

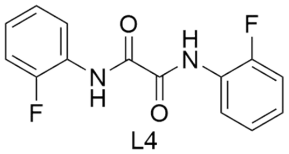

L4

The L4 was prepared as a white solid in 82% yield from 2-fluoroaniline and oxalyl chloride following the general procedure A. M.P.: 231-232°C. ^{1}H NMR (400 MHz, DMSO-d_6) δ 10.56 (s, 2H), 7.67 (t, *J* = 7.8 Hz, 2H), 7.41 – 7.17 (m, 6H). ^{13}C NMR (101 MHz, DMSO-d_6) δ 158.30, 155.32 (d, *J* = 247.6 Hz), 127.65 (d, *J* = 7.7 Hz), 126.15 (d),

124.58 (d, J = 3.9 Hz), 124.32 (d, J = 12.0 Hz), 115.99 (d, J = 19.7 Hz); ESI-HRMS m/z calcd for $C_{14}H_{11}F_2N_2O_2$ $(M + H)^+$ 277.0975, found: 277.0977.

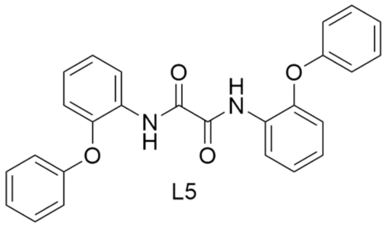

L5

The L5 was prepared as a white solid in 81% yield from 2-phenoxyaniline and oxalyl chloride following the general procedure A. M.P.: 206-207°C. ^{1}H NMR (400 MHz, $CDCl_3$) δ 9.86 (s, 2H), 8.36 (d, J = 8.0 Hz, 2H), 7.28 (s, 1H), 7.24 (s, 1H), 7.16 – 6.74 (m, 14H). ^{13}C NMR (101 MHz, $CDCl_3$) δ 157.50, 156.20, 146.87, 130.12, 128.11, 125.55, 124.33, 123.89, 120.61, 119.18, 117.73; ESI-HRMS m/z calcd for $C_{26}H_{21}N_2O_4$ $(M + H)^+$ 425. 0955, found: 425. 0957.

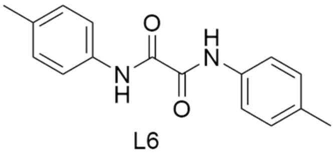

L6

The L6 was prepared as a white solid in 88% yield from 4-methylaniline and oxalyl chloride following the general procedure A. M.P.: 280-281°C. ^{1}H NMR (400 MHz, DMSO-d_6) δ 9.97 (s, 2H), 7.76 – 6.76 (m, 8H), 3.94 (s, 6H). ^{13}C NMR (101 MHz, DMSO-d_6) δ 157.56, 148.96, 129.92, 126.26, 125.38, 121.11, 119.78, 110.37, 55.91, 21.14; ESI-HRMS m/z calcd for $C_{16}H_{17}N_2O_4$ $(M + H)^+$ 301.0945, found: 301.0943.

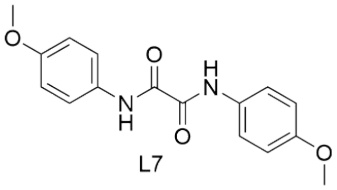

L7

The L7 was prepared as a white solid in 87% yield from 4-methoxyaniline and oxalyl chloride following the general procedure A. M.P.: 268-269°C. ^{1}H NMR (400 MHz, DMSO-d_6) δ 10.72 (s, 2H), 7.89 – 7.64 (m, 4H), 7.07 – 6.81 (m, 4H), 3.74 (s, 6H). ^{13}C NMR (101 MHz, DMSO-d_6) δ 158.30, 156.14, 130.82, 121.89, 113.89; ESI-HRMS m/z calcd for $C_{16}H_{17}N_2O_4$ $(M + H)^+$ 301.0945, found: 301.0943.

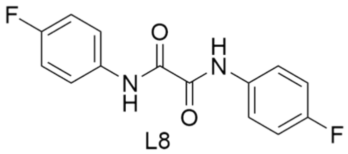

L8

The L8 was prepared as a white solid in 89% yield from 4-fluoroaniline and oxalyl chloride following the general procedure A. M.P.: 256-258^oC. ^1H NMR (400 MHz, DMSO-d_6) δ 7.89 (dd, *J* = 9.3, 5.0 Hz, 4H), 7.22 (d, *J* = 9.0 Hz, 4H).^{13}C NMR (101 MHz, DMSO-d_6) δ 160.05, 158.06 (d, *J* = 81.9 Hz), 134.11 (d, *J* = 2.9 Hz), 122.38 (d, *J* = 7.7 Hz), 115.45 (d, *J* = 22.2 Hz); ESI-HRMS m/z calcd for $C_{14}H_{10}F_2N_2O_2$ (M + H)$^+$ 277.0983, found: 277.0985.

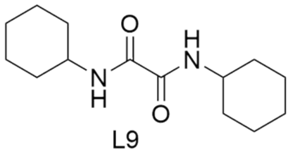

L9

The L9 was prepared as a white solid in 92% yield from cyclohexylamine and oxalyl chloride following the general procedure A. M.P.: 276-277^oC. ^1H NMR (400 MHz, $CDCl_3$) δ 7.39 (d, *J* = 9.3 Hz, 2H), 3.95 – 3.50 (m, 2H), 1.95 – 1.60 (m, 10H), 1.42 – 1.12 (m, 10H). ^{13}C NMR (101 MHz, $CDCl_3$) δ 159.18, 48.88, 32.75, 25.48, 24.84; ESI-HRMS m/z calcd for $C_{14}H_{25}N_2O_2$ (M + H)$^+$ 253.1965, found: 253.1967.

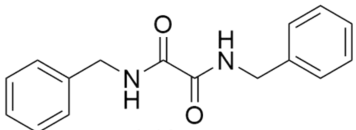

L10

The L10 was prepared as a white solid in 91% yield from benzylamine and oxalyl chloride following the general procedure A. M.P.: 225-227^oC. ^1H NMR (400 MHz, DMSO-d_6) δ 9.34 (t, *J* = 6.6 Hz, 2H), 7.28 (dq, *J* = 15.6, 8.0 Hz, 10H), 4.33 (d, *J* = 6.5 Hz, 4H).^{13}C NMR (101 MHz, DMSO-d_6) δ 160.16, 138.78, 128.34, 127.40, 126.98, 42.41; ESI-HRMS m/z calcd for $C_{16}H_{17}N_2O_2$ (M + H)$^+$ 269.0973, found: 269. 0971.

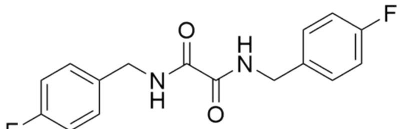

L11

The L11 was prepared as a white solid in 90% yield from 4-flubenzylamine and oxalyl chloride following the general procedure A. M.P.: 235-237^oC. ^1H NMR (400 MHz, DMSO-d_6) δ 9.36 (t, *J* = 6.5 Hz, 2H), 7.42 – 7.00 (m, 8H), 4.30 (d, *J* = 6.5 Hz, 4H).^{13}C NMR

(101 MHz, DMSO-d_6) δ 162.46, 160.08 (d, *J* = 4.8 Hz), 134.98 (d, *J* = 2.9 Hz), 129.48 (d, *J* = 8.2 Hz), 115.06 (d, *J* = 21.2 Hz), 41.72; ESI-HRMS m/z calcd for $C_{16}H_{15}F_2N_2O_2$ $(M + H)^+$ 305.0985, found: 305.0983.

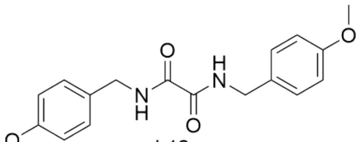

L12

The L12 was prepared as a white solid in 89% yield from 4-methoxybenzylamine and oxalyl chloride following the general procedure A. M.P.: 242-243°C. ^1H NMR (400 MHz, DMSO-d_6) δ 9.24 (t, *J* = 6.7 Hz, 2H), 7.19 (d, *J* = 8.6 Hz, 4H), 6.86 (d, *J* = 8.7 Hz, 4H), 4.24 (d, *J* = 6.5 Hz, 4H), 3.71 (s, 6H). ^{13}C NMR (101 MHz, DMSO-d_6) δ 162.76, 160.01, 158.29, 139.37, 130.78, 128.83, 113.68, 41.82; ESI-HRMS m/z calcd for $C_{18}H_{21}N_2O_4$ $(M + H)^+$ 329.0956, found: 329.0954.

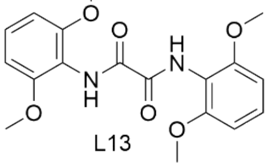

L13

The L13 was prepared as a white solid in 85% yield from 2, 6-dimethoxyaniline and oxalyl chloride following the general procedure A. M.P.: 269-270°C. ^1H NMR (400 MHz, DMSO-d_6) δ 9.54 (s, 2H), 7.27 (t, *J* = 8.4 Hz, 2H), 6.73 (d, *J* = 8.5 Hz, 4H), 3.77 (s, 12H).^{13}C NMR (101 MHz, DMSO-d_6) δ 158.66, 155.81, 128.24, 113.33, 104.40 (d, *J* = 33.2 Hz), 55.75;ESI-HRMS m/z calcd for $C_{18}H_{21}N_2O_6$ $(M + H)^+$ 361.0987, found: 361.0988.

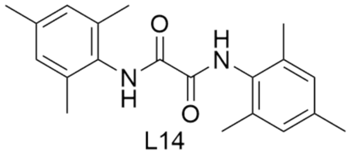

L14

The L14 was prepared as a white solid in 84% yield from 2,4,6-trimethylaniline and oxalyl chloride following the general procedure A. M.P.: 288-291°C. ^1H NMR (400 MHz, $CDCl_3$) δ 8.81 (s, 2H), 6.94 (s, 4H), 2.27 (d, *J* = 25.7 Hz, 18H). ^{13}C NMR (101 MHz, $CDCl_3$) δ 158.40, 137.76, 134.82, 129.79, 129.24, 21.09, 18.48; ESI-HRMS m/z calcd for $C_{20}H_{25}N_2O_2$ $(M + H)^+$ 325.1968, found: 325.1969.

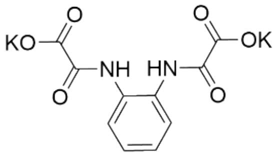

L15

The L15 was prepared as a light yellow solid in 92% yield from 1, 2-phenylenediamine, mono-methyl oxalyl chloride and KOH following the general procedure B. M.P.: 311-314°C. ^{1}H NMR (400 MHz, D_2O) δ 7.46 (dd, *J* = 6.1, 3.5 Hz, 2H), 7.28 (dd, *J* = 6.1, 3.5 Hz, 2H). ^{13}C NMR (101 MHz, D_2O) δ 165.45, 163.70, 129.83, 127.59, 125.81; ESI-HRMS m/z calcd for $C_{10}H_6N_2O_6$(M - K)$^-$ 249.989, found: 249.992.

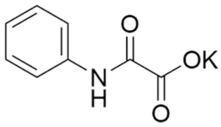

L16

The L16 was prepared as a light yellow solid in 90% yield from aniline, mono-methyl oxalyl chloride and KOH following the general procedure B. M.P.: >324°C. ^{1}H NMR (400 MHz, D_2O) δ 7.44 – 7.27 (m, 4H), 7.15 (t, *J* = 7.4 Hz, 1H). ^{13}C NMR (101 MHz, D_2O) δ 166.12, 163.29, 136.25, 129.23, 126.05, 121.92; ESI-HRMS m/z calcd for $C_8H_6NO_3$ (M - K)$^-$ 163.985, found: 163.982.

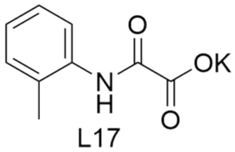

L17

The L17 was prepared as a light yellow solid in 89% yield from 2-methylaniline, mono-methyl oxalyl chloride and KOH following the general procedure B. M.P.: 271-273°C.^{1}H NMR (400 MHz, D_2O) δ 7.30 – 7.13 (m, 4H), 2.13 (s, 3H). ^{13}C NMR (101 MHz, D_2O) δ 166.08, 164.13, 133.99, 130.78, 127.58, 126.68, 125.80, 16.84; ESI-HRMS m/z calcd for $C_9H_8NO_3$ (M - K)$^-$ 178.0765, found: 178.0768.

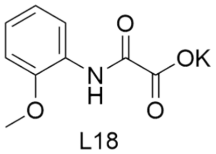

L18

The L18 was prepared as a light yellow solid in 86% yield from 2-methoxyaniline, mono-methyl oxalyl chloride and KOH following the general procedure B. M.P.: >324°C. ^{1}H NMR (400 MHz, D_2O) δ 7.68 (dd, *J* = 7.9, 2.2 Hz, 1H), 7.21 – 7.04 (m, 1H),

7.06 – 6.81 (m, 2H), 3.76 (d, *J* = 4.6 Hz, 3H). ^{13}C NMR (101 MHz, D_2O) δ 165.73, 162.68, 150.55, 127.01, 125.08, 122.39, 121.05, 111.92, 55.92; ESI-HRMS m/z calcd for $C_9H_8NO_4$ (M - K)$^-$ 193.975, found: 193.977.

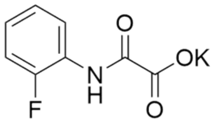

L19

The L19 was prepared as a light yellow solid in 83% yield from 2-fluoroaniline, mono-methyl oxalyl chloride and KOH following the general procedure B. M.P.: 295-296°C. ^{1}H NMR (400 MHz, D_2O) δ 7.56 (td, *J* = 7.7, 1.7 Hz, 1H), 7.32 – 6.98 (m, 3H). ^{13}C NMR (101 MHz, D_2O) δ 164.59 (d, *J* = 185.4 Hz), 155.09 (d, *J* = 244.7 Hz), 149.91, 134.87, 124.41 (d, *J* = 154.6 Hz). ESI-HRMS m/z calcd for $C_8H_5FNO_3$ (M - K)$^-$ 181.953, found: 181.956.

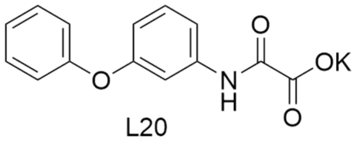

L20

The L20 was prepared as a light yellow solid in 82% yield from 2-phenoxyaniline, mono-methyl oxalyl chloride and KOH following the general procedure B. M.P.: 250-253°C.^{1}H NMR (400 MHz, D_2O) δ 7.77 (d, *J* = 8.0 Hz, 1H), 7.03 (t, *J* = 7.9 Hz, 2H), 6.92 (t, *J* = 7.8 Hz, 1H), 6.84 (t, *J* = 7.4 Hz, 1H), 6.78 (t, *J* = 7.8 Hz, 1H), 6.66 (d, *J* = 8.2 Hz, 2H), 6.54 (d, *J* = 8.1 Hz, 1H). ^{13}C NMR (101 MHz, D_2O) δ 165.10, 162.39, 156.27, 147.41, 130.14, 129.82, 127.76, 126.39, 124.39, 123.94, 122.89, 118.05, 117.47; ESI-HRMS m/z calcd for $C_{14}H_{10}NO_4$ (M - K)$^-$ 256.0987, found: 256.0983.

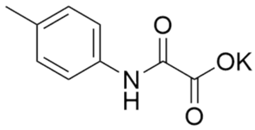

L21

The L21 was prepared as a light yellow solid in 89% yield from 4-methylaniline, mono-methyl oxalyl chloride and KOH following the general procedure B. ^{1}H NMR (400 MHz, D_2O) δ 7.27 (dd, *J* = 8.3, 3.2 Hz, 2H), 7.14 (dd, *J* = 8.6, 3.0 Hz, 2H), 2.20 (d, *J* = 3.2 Hz, 3H). ^{13}C NMR (101 MHz, D_2O) δ 166.18, 163.18, 136.17, 133.62, 129.69, 121.96, 20.10; ESI-HRMS m/z calcd for $C_9H_8NO_3$ (M - K)$^-$ 178.0989, found: 178.1.0986.

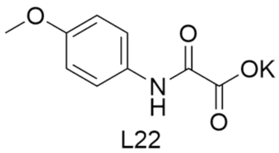

L22

The L22 was prepared as a light yellow solid in 81% yield from 4-methoxyaniline, mono-methyl oxalyl chloride and KOH following the general procedure B. M.P.: 307-310ºC . ^{1}H NMR (400 MHz, D_2O) δ 7.30 (d, *J* = 8.5 Hz, 2H), 6.88 (d, *J* = 9.1 Hz, 2H), 3.70 (s, 3H). ^{13}C NMR (101 MHz, D_2O) δ 166.18, 163.23, 156.70, 129.57, 123.86, 114.45, 55.52; ESI-HRMS m/z calcd for $C_9H_8NO_4$ $(M - K)^-$ 193.973, found: 193.975.

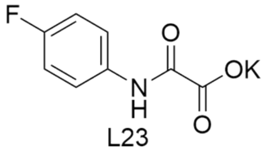

L23

The L23 was prepared as a light yellow solid in 88% yield from 4-fluoroaniline, mono-methyl oxalyl chloride and KOH following the general procedure B. M.P.: >324ºC. ^{1}H NMR (400 MHz, D_2O) δ 7.66 – 7.27 (m, 2H), 7.10 (dt, *J* = 49.9, 8.9 Hz, 2H). ^{13}C NMR (101 MHz, D_2O) δ 174.55, 165.61, 132.22, 125.01 (d, *J* = 171.6 Hz), 114.73 (d, *J* = 212.9 Hz); ESI-HRMS m/z calcd for $C_8H_5FNO_3$ $(M - K)^-$ 181.964, found: 181.971.

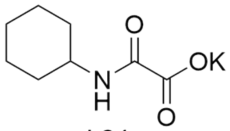

L24

The L24 was prepared as a light yellow solid in 92% yield from cyclohexylamine, mono-methyl oxalyl chloride and KOH following the general procedure B.M.P.: 252-257ºC. ^{1}H NMR (400 MHz, D_2O) δ 3.47 (t, *J* = 10.5 Hz, 1H), 1.84 – 1.36 (m, 5H), 1.29 – 1.01 (m, 5H). ^{13}C NMR (101 MHz, D_2O) δ 166.22, 165.24, 163.09, 160.68, 133.44, 129.11, 115.39, 42.37; ESI-HRMS m/z calcd for $C_8H_{12}NO_3$ $(M - K)^-$ 170.0948, found: 170.0946.

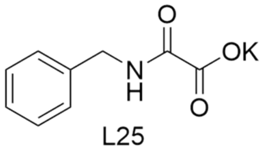

L25

The L25 was prepared as a light yellow solid in 90% yield from benzylamine, mono-methyl oxalyl chloride and KOH following the general procedure B. M.P.: 251-256ºC. ^{1}H NMR (400 MHz, D_2O) δ 7.41 – 7.07 (m, 5H), 4.31 (s, 2H). ^{13}C NMR (101 MHz,

D_2O) δ 166.23, 165.24, 137.63, 127.34, 127.00, 42.96; ESI-HRMS m/z calcd for $C_9H_8NO_3$ $(M - K)^-$ 178.0965, found: 178.0963.

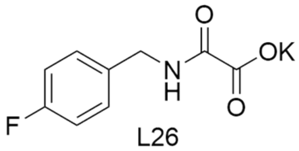

L26

The L26 was prepared as a light yellow solid in 89% yield from 4-flubenzylamine, mono-methyl oxalyl chloride and KOH following the general procedure B. M.P.: 263-267^oC. ^1H NMR (400 MHz, D_2O) δ 7.45 – 7.04 (m, 4H), 4.31 (s, 2H). ^{13}C NMR (101 MHz, D_2O) δ 166.23, 165.24, 137.63, 128.64 (d, J = 599.0 Hz), 127.17 (d, J = 34.2 Hz), 42.96; ESI-HRMS m/z calcd for $C_9H_7FNO_3$ $(M - K)^-$ 195.8673, found: 195.8671.

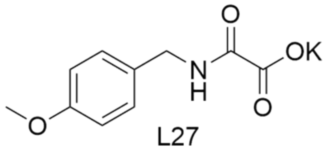

L27

The L27 was prepared as a light yellow solid in 93% yield from 4-methoxybenzylamine, mono-methyl oxalyl chloride and KOH following the general procedure B. M.P.: 273-276^oC. ^1H NMR (400 MHz, D_2O) δ 7.17 (d, J = 8.7 Hz, 2H), 6.86 (d, J = 8.7 Hz, 2H), 4.25 (s, 2H), 3.70 (s, 3H). ^{13}C NMR (101 MHz, D_2O) δ 166.30, 165.13, 158.12, 130.32, 128.96, 128.67, 114.30, 114.14, 55.33, 42.42; ESI-HRMS m/z calcd for $C_{10}H_{10}NO_4$ $(M - K)^-$ 208.0973, found: 208.0966.

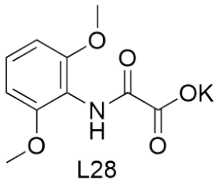

L28

The L28 was prepared as a light yellow solid in 85% yield from 2, 6-dimethoxyaniline, mono-methyl oxalyl chloride and KOH following the general procedure B. M.P.: 152-156^oC.^1H NMR (400 MHz, D_2O) δ 7.24 (t, J = 8.5 Hz, 1H), 6.67 (d, J = 8.6 Hz, 2H), 3.70 (s, 6H). ^{13}C NMR (101 MHz, D_2O) δ 165.66, 164.57, 155.28, 129.61, 129.32, 112.29, 105.77, 104.58, 56.22; ESI-HRMS m/z calcd for $C_{10}H_{10}NO_5$ $(M - K)^-$ 224.0967, found: 224.0957.

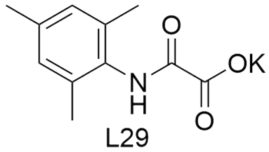

L29

The L29 was prepared as a light yellow solid in 86% yield from 2,4,6-trimethylaniline, mono-methyl oxalyl chloride and KOH following the general procedure B. M.P.: 289-292°C. ^{1}H NMR (400 MHz, D_2O) δ 6.90 (s, 2H), 2.15 (s, 3H), 2.01 (s, 6H). ^{13}C NMR (101 MHz, D_2O) δ 166.15, 164.73, 138.33, 135.67, 130.12, 128.72, 128.58, 20.06, 17.10; ESI-HRMS m/z calcd for $C_{11}H_{12}NO_3$ (M - K)$^-$ 206.0668, found: 206.0671.

3. General procedure for copper-catalyzed 2-bromophenol coupling

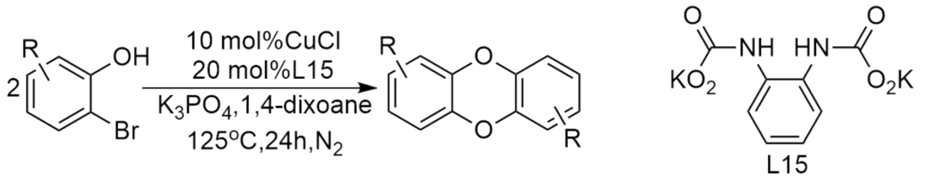

The 2-bromophenol (2.0mmol),CuCl (0.1mmol,10.1mg),K_3PO_4 (3.0mmol, 643.7mg) and ligand L15 (0.2mmol,65.9mg) were placed into a Schlenk tube (25mL) with a magnetic stirring rod. Evacuate the reaction vessel and backfill with nitrogen, cycle three times, then add 1,4-dioxane (3.0mL) (note: for liquid substances, they are added after backfilling the tube with nitrogen). Under intense agitation, the reaction mixture is heated at 125°C for 24 hours. The cooled solution was diluted with ethyl acetate, concentrated in vacuum, and purified by silica gel chromatography to obtain the corresponding cyclic diether.

As shown in Table 1, we selected L17/L28 with medium performance and copper salt to catalyze the coupling of 2-bromophenol as a model reaction to screen the best copper salt, alkali and solvent. It was found that the coupling effect of CuCl as a catalyst was better than that of CuI (items 1-8). When K_3PO_4 was used to provide an alkaline environment, the coupling effect of 2-bromophenol was the best (items 9-16). After a series of solvents were tried, it was found that 1,4-dioxane showed outstanding advantages as a solvent (items 10-20). In order to further explore the best 2-bromophenol coupling reaction conditions, we summarized the previous research results and carried out a complete condition optimization experiment. The coupling reactions under different temperatures, different reaction times, different catalyst dosages and different ligand inputs were compared. It was found that 125°C was the most suitable reaction temperature (compared with items 32,34,35). The effect of L15 with CuCl catalytic system was the most prominent (compared with items 27-32), the conversion rate could reach 85%, and the separation yield reached 82%. It was found that the conversion rate did not increase with the longer reaction time (compare items 32-33). The use of copper salt is 10mol% (comparison items 32-36), and the best effect is 20mol% of amide ligand (comparison items 37-38).

Table 1 Coupling of 1a catalyzed by Cu salt under different reaction conditions

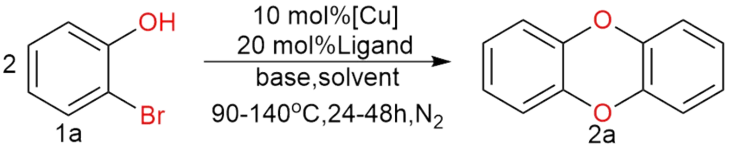

entry[a]	ligand	catalyst	base	solvent	yield(%)[b]
1	L28	CuCl	K_3PO_4	1,4-dixoane	71
2	L28	CuBr	K_3PO_4	1,4-dixoane	58
3	L28	CuO	K_3PO_4	1,4-dixoane	45
4	L28	CuC_2O_4	K_3PO_4	1,4-dixoane	55
5	L28	Cu_2O	K_3PO_4	1,4-dixoane	45
6	L28	$Cu(OAc)_2$	K_3PO_4	1,4-dixoane	61
7	L28	$Cu(OTf)_2$	K_3PO_4	1,4-dixoane	49
8	L28	$Cu(acac)_2$	K_3PO_4	1,4-dixoane	55
9	L28	CuI	K_2CO_3	1,4-dixoane	49
10	L28	CuI	KOH	1,4-dixoane	37
11	L28	CuI	NaOH	1,4-dixoane	35
12	L28	CuI	KOAc	1,4-dixoane	10
13	L28	CuI	Cs_2CO_3	1,4-dixoane	38
14	L28	CuI	Cs_2WO_4	1,4-dixoane	23
15	L28	CuI	CsF	1,4-dixoane	30
16	L28	CuI	KO^tBu	1,4-dixoane	41
17	L17	CuI	K_3PO_4	DMSO	13
18	L17	CuI	K_3PO_4	DMF	10
19	L17	CuI	K_3PO_4	CH_3CN	32
20	L17	CuI	K_3PO_4	DMC	38
21	L17	CuI	K_3PO_4	DEC	35
22	L17	CuI	K_3PO_4	CH_3COOH	39
23	L17	CuI	K_3PO_4	Toluene	50
24	L17	CuI	K_3PO_4	Xylene	29
25	L17	CuI	K_3PO_4	EtOH	38

…

26	L15	CuI	K_3PO_4	H_2O	0
27	L17	CuI	K_3PO_4	1,4-dixoane	59
28	L28	CuI	K_3PO_4	1,4-dixoane	66
29	L15	CuI	K_3PO_4	1,4-dixoane	70
30	L17	CuCl	K_3PO_4	1,4-dixoane	68
31	L28	CuCl	K_3PO_4	1,4-dixoane	68
32	L15	CuCl	K_3PO_4	1,4-dixoane	85[k]
33[c]	L15	CuCl	K_3PO_4	1,4-dixoane	79
34[d]	L15	CuCl	K_3PO_4	1,4-dixoane	30-67
35[e]	L15	CuCl	K_3PO_4	1,4-dixoane	78
36[f]	L15	CuCl	K_3PO_4	1,4-dixoane	79
37[g]	L15	CuCl	K_3PO_4	1,4-dixoane	84
38[h]	L15	CuCl	K_3PO_4	1,4-dixoane	76
39[i]	L15	CuCl	K_3PO_4	1,4-dixoane	0
40[j]	L15	CuCl	K_3PO_4	1,4-dixoane	5
41[l]	L15	CuCl	K_3PO_4	1,4-dixoane	10
42	L15	CuCl	none	1,4-dixoane	10

[a] general situation is as follows : 1a (2.0mmol), copper salt (0.1mmol), ligand (0.2mmol), base (3.0mmol), solvent (3.0mL), in N_2 atmosphere, reaction 24h. [b] Using n-decane as an internal standard, [c] reaction 48h, [d] reaction temperature: 50-90°C, [e] reaction temperature:140°C, [f] L15 (0.4mmol), [g] CuCl (0.2mmol), [h] CuCl (0.05mmol), [i] no copper salt, [j] no ligand, [k] separation yield of 82%,[l] air instead of N_2.

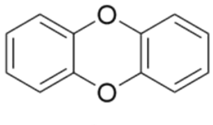

2a

2a:White powder（150.9mg,82%）, M.P.: 120-121°C. ^{1}H NMR (400 MHz, $CDCl_3$) δ 6.95 – 6.78 (m, 8H). ^{13}C NMR (101 MHz, $CDCl_3$) δ 142.36, 123.94, 116.51; ESI-HRMS m/z calcd for $C_{12}H_8O_2$ $(M + H)^+$ 185.0967, found: 185. 0967.

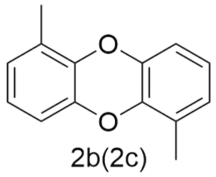

2b(2c)

2b(2c):White powder（2b:156.9mg,74%;2c:161.1mg,76%）, M.P.: 164-165°C. ^{1}H NMR (400 MHz, $CDCl_3$) δ 6.91 – 6.49 (m, 6H), 2.24 (s, 6H). ^{13}C NMR (101 MHz, $CDCl_3$) δ 142.38, 140.60, 126.02, 125.42, 122.80, 114.05, 15.25; ESI-HRMS m/z calcd for $C_{14}H_{12}O_2$ $(M + H)^+$ 213.0977, found: 213.0983.

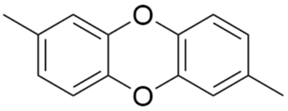

2d(2e)

2d(2e): White powder（2d:169.6mg,80%;2e:171.8mg,81%）, M.P.: 108-112°C .^{1}H NMR (400 MHz, $CDCl_3$) δ 6.81 – 6.58 (m, 6H), 2.24 (s, 6H). ^{13}C NMR (101 MHz, $CDCl_3$) δ 142.03, 140.03, 133.67, 123.99, 116.94, 116.05, 20.83; ESI-HRMS m/z calcd for $C_{14}H_{12}O_2$ $(M + H)^+$ 213.0867, found: 213.0871.

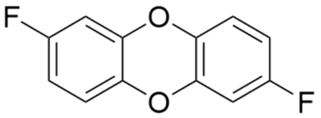

2f(2g)

2f(2g): White powder (2f:162.8mg,74%;2g:167.2mg,76%) , M.P.: 145-146°C. ^{1}H NMR (400 MHz, $CDCl_3$) δ 6.78 (d, *J* = 4.1 Hz, 2H), 6.59 (dd, *J* = 8.2, 4.6 Hz, 4H). ^{13}C NMR (101 MHz, $CDCl_3$) δ 158.83 (d, *J* = 242.5 Hz), 142.50 (d, *J* = 12.3 Hz), 137.68 (d, *J* = 3.2 Hz), 116.84 (d, *J* = 9.5 Hz), 110.11 (d, *J* = 23.2 Hz), 104.42 (d, *J* = 27.5 Hz); ESI-HRMS m/z calcd for $C_{12}H_6F_2O_2$ $(M + H)^+$ 220.781, found: 220.784.

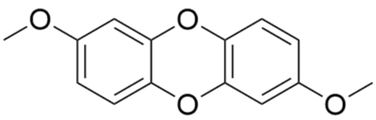

2h(2i)

2h(2i): White powder (2h:187.9mg,77%;2i:183.1mg,75%) , M.P.: 139-140°C. ^{1}H NMR (400 MHz, $CDCl_3$) δ 6.76 (d, *J* = 12.0 Hz, 2H), 6.43 (s, 4H), 3.75 (s, 6H); ^{13}C NMR (101 MHz, $CDCl_3$) δ 156.13, 142.82, 135.75, 116.54, 108.31, 102.60, 55.84; ESI-HRMS m/z calcd for $C_{14}H_{12}O_4$ $(M + H)^+$ 245.0784, found: 245.0789.

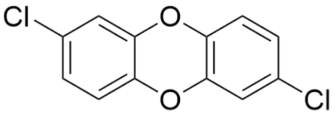

2j(2k)

2j(2k): White powder (2j:186.5mg,74%;2k:183.9mg,73%) , M.P.: 159-160°C. ^{1}H NMR (400 MHz, $CDCl_3$) δ 6.87 (dd, *J* = 10.8, 2.3 Hz, 4H), 6.77 (d, *J* = 8.3 Hz, 2H). ^{13}C NMR (101 MHz, $CDCl_3$) δ 142.37, 140.46, 128.87, 124.04, 117.37, 116.95; ESI-HRMS m/z calcd for $C_{12}H_6Cl_2O_2$ $(M + H)^+$ 252.8782, found: 252.8774.

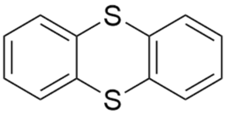

2n

2n: White powder (162.1mg,75%) , M.P.: 89-92°C. ^{1}H NMR (400 MHz, $CDCl_3$) δ 7.00 (s, 4H), 6.81 (s, 4H). ^{13}C NMR (101 MHz, $CDCl_3$) δ 136.24, 133.06, 128.34, 128.05, 127.02, 121.15. ESI-HRMS m/z calcd for $C_{12}H_8S_2$ $(M + H)^+$ 216.9867, found: 216.9865.

4. Copies of ^{1}H and ^{13}C spectra of ligands and (hetero) dibenzoxins

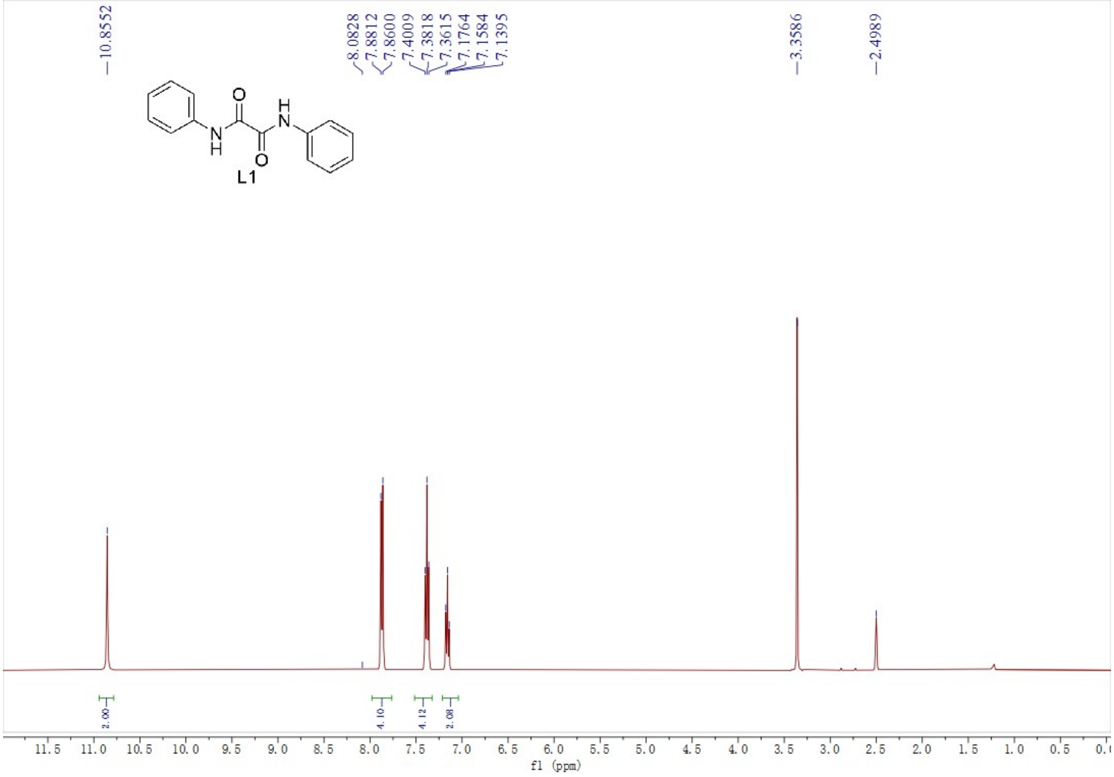

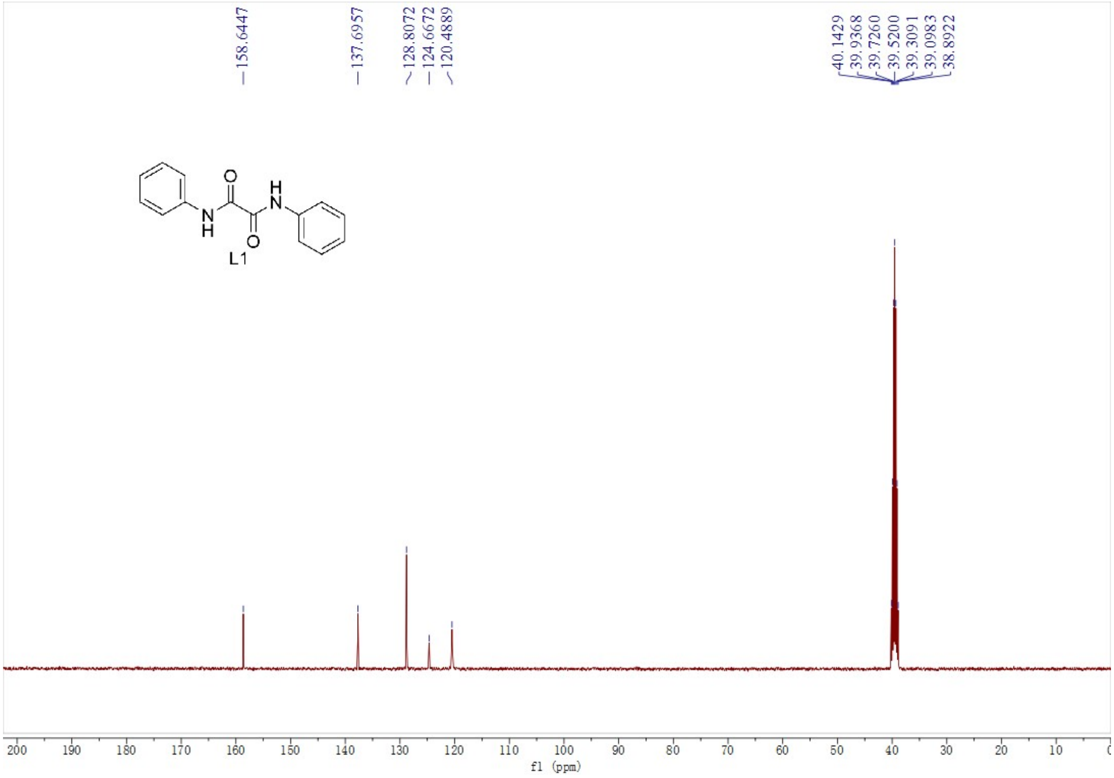

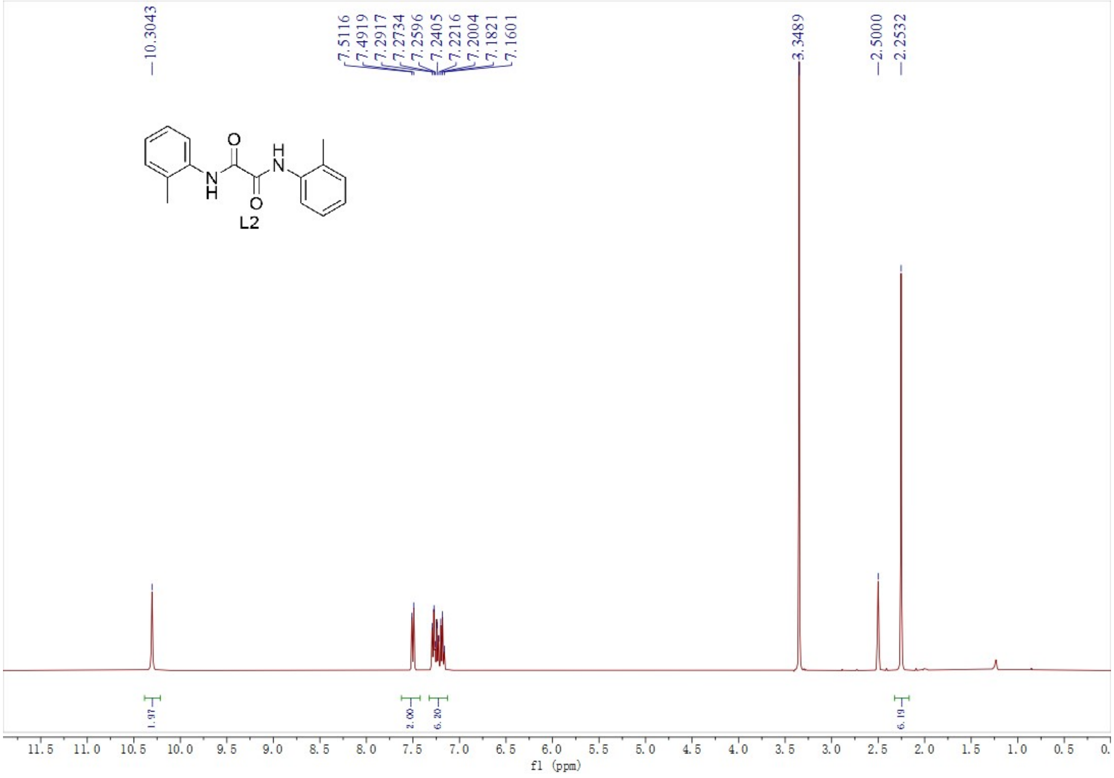
10.3043
7.5116
7.4919
7.2917
7.2734
7.2596
7.2405
7.2216
7.2004
7.1821
7.1601
3.3489
2.5000
2.2532
L2
1.97
2.00
6.20
6.19
11.5 11.0 10.5 10.0 9.5 9.0 8.5 8.0 7.5 7.0 6.5 6.0 5.5 5.0 4.5 4.0 3.5 3.0 2.5 2.0 1.5 1.0 0.5
f1 (ppm)

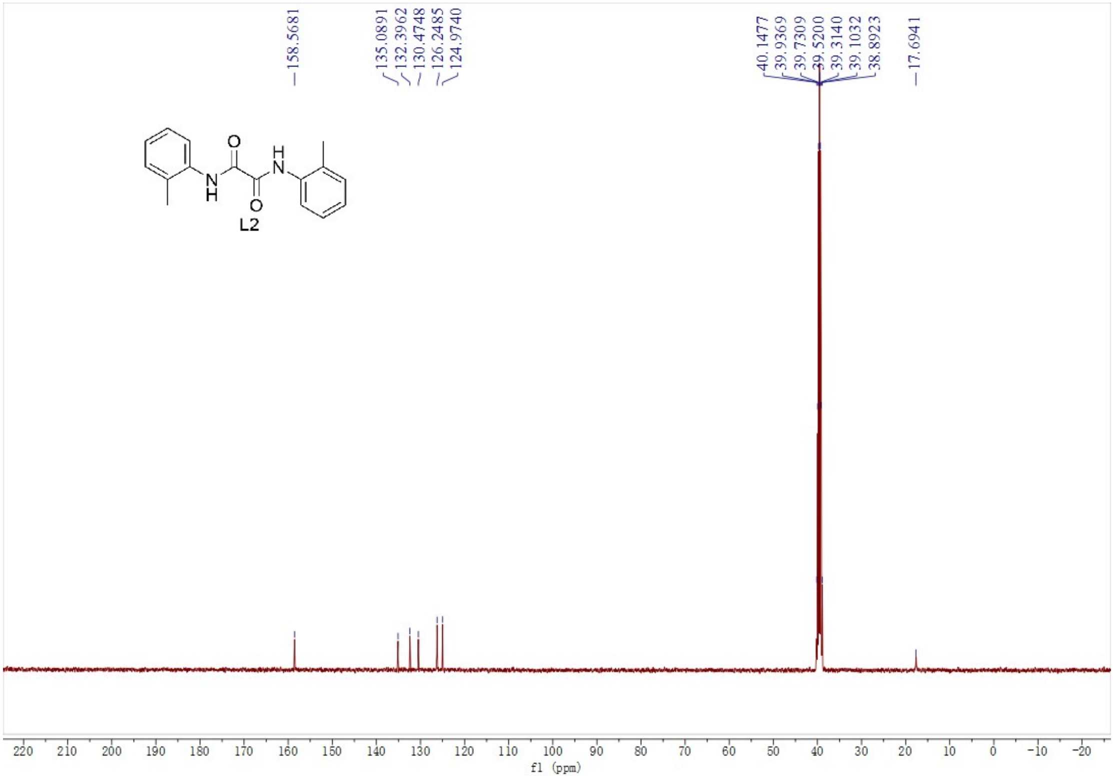
158.5681
135.0891
132.3962
130.4748
126.2485
124.9740
40.1477
39.9369
39.7309
39.5200
39.3140
39.1032
38.8923
17.6941
L2
220 210 200 190 180 170 160 150 140 130 120 110 100 90 80 70 60 50 40 30 20 10 0 -10 -20
f1 (ppm)

…

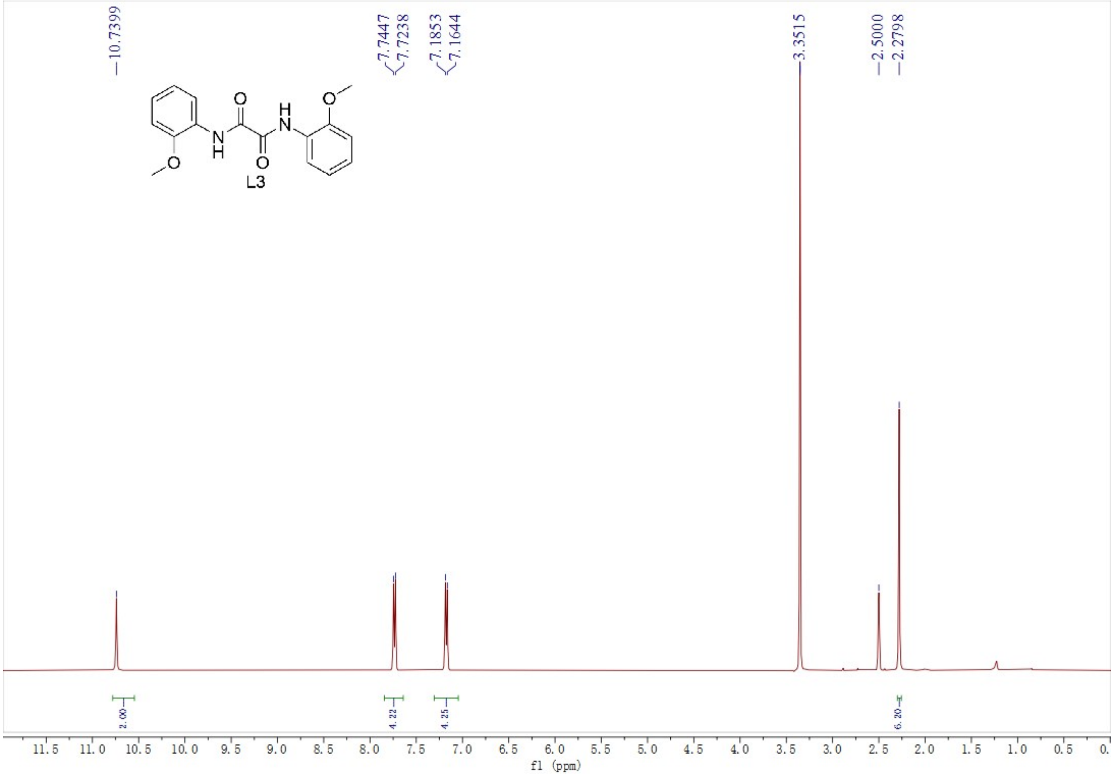
10.7399
7.7447
7.7238
7.1853
7.1644
3.3515
2.5000
2.2798
L3
2.00
4.22
4.25
6.20
f1 (ppm)

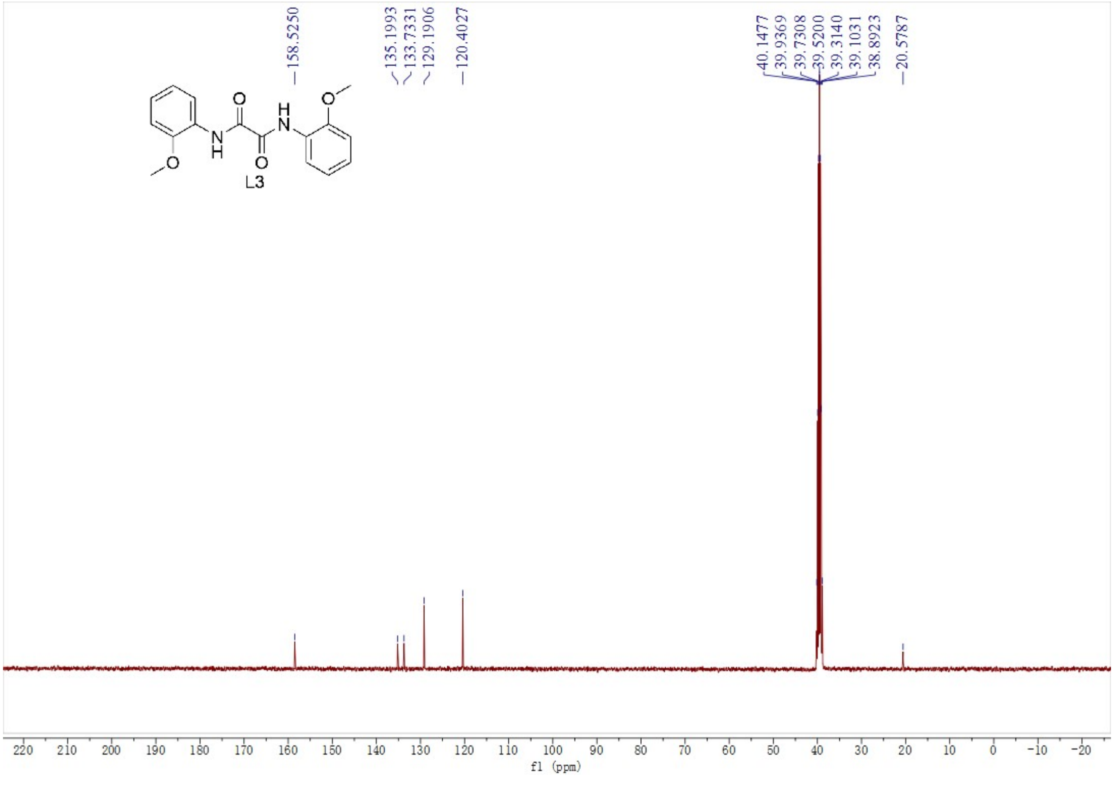
158.5250
135.1993
133.7331
129.1906
120.4027
40.1477
39.9369
39.7308
39.5200
39.3140
39.1031
38.8923
20.5787
L3
f1 (ppm)

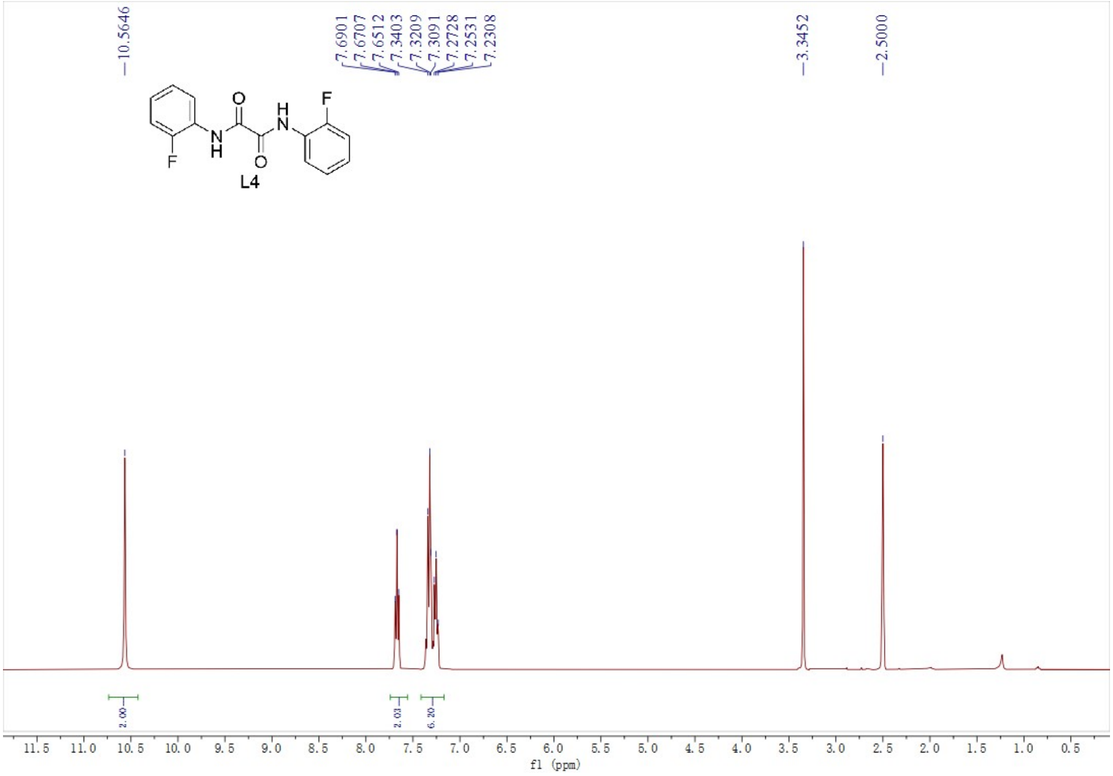
10.5646
7.6901
7.6707
7.6512
7.3403
7.3209
7.3091
7.2728
7.2531
7.2308
3.3452
2.5000
L4
2.00
2.03
6.20
11.5 11.0 10.5 10.0 9.5 9.0 8.5 8.0 7.5 7.0 6.5 6.0 5.5 5.0 4.5 4.0 3.5 3.0 2.5 2.0 1.5 1.0 0.5
f1 (ppm)

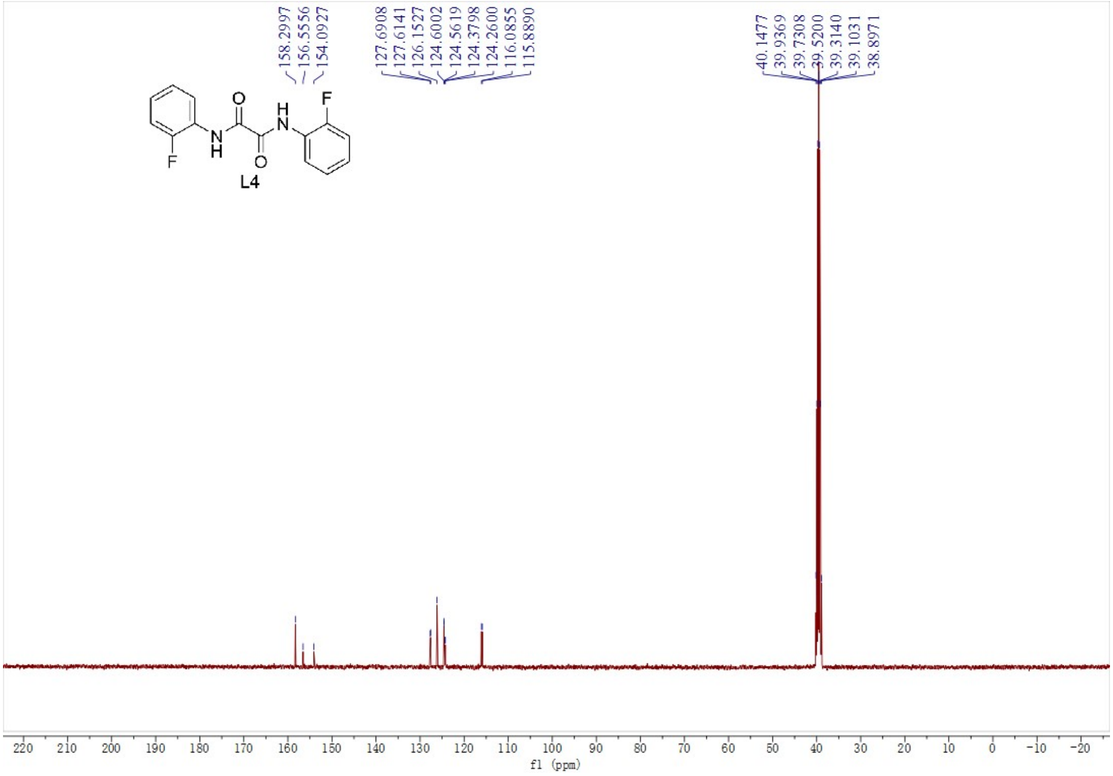
158.2997
156.5556
154.0927
127.6908
127.6141
126.1527
124.6002
124.5619
124.3798
124.2600
116.0855
115.8890
40.1477
39.9369
39.7308
39.5200
39.3140
39.1031
38.8971
L4
220 210 200 190 180 170 160 150 140 130 120 110 100 90 80 70 60 50 40 30 20 10 0 -10 -20
f1 (ppm)

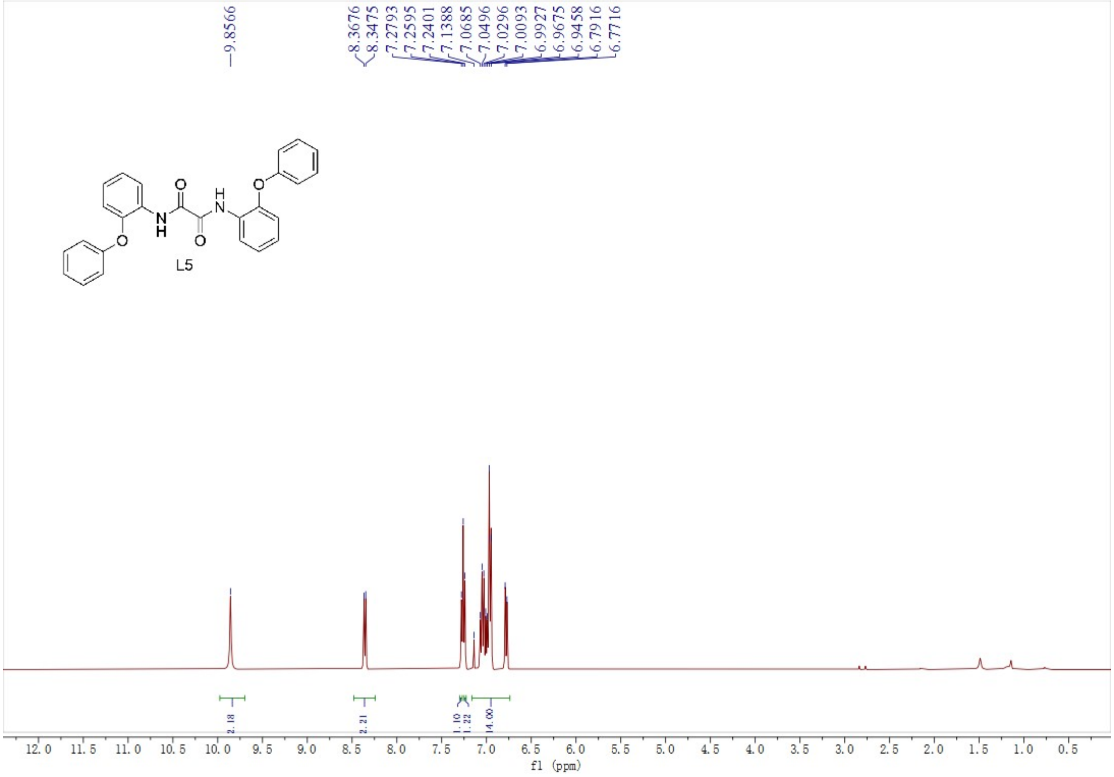

9.8566
8.3676
8.3475
7.2793
7.2595
7.2401
7.1388
7.0685
7.0496
7.0296
7.0093
6.9927
6.9675
6.9458
6.7916
6.7716
L5
2.18
2.21
1.10
1.22
14.00
f1 (ppm)

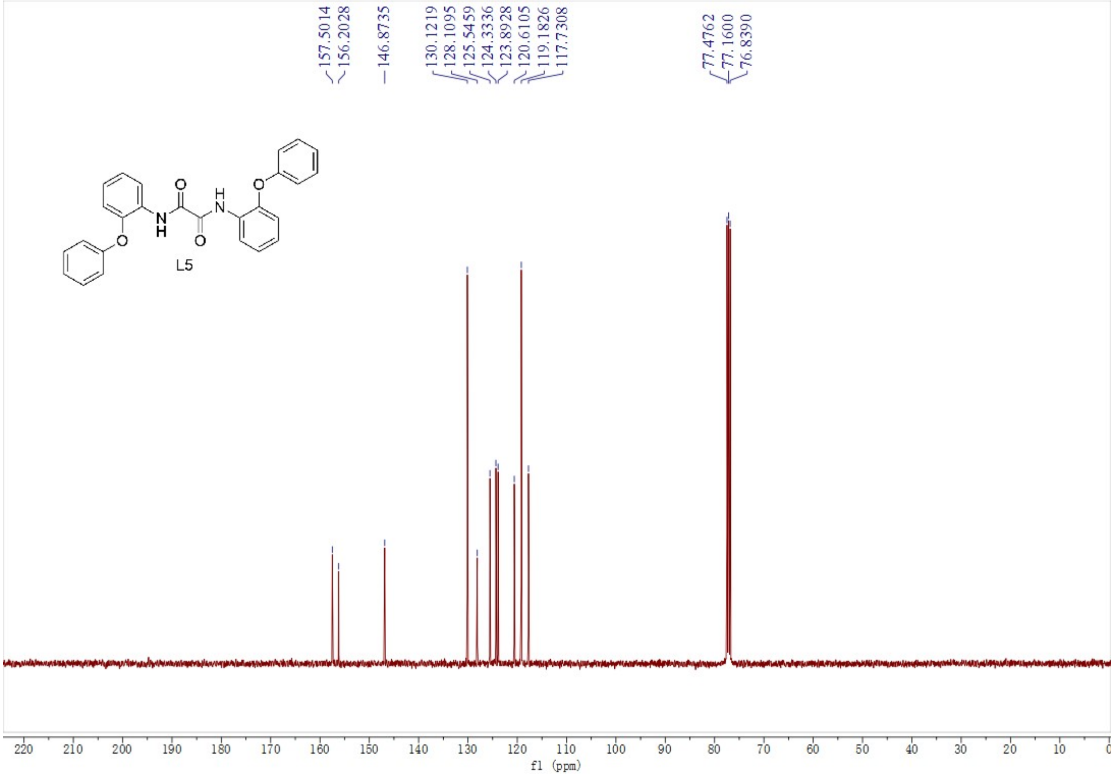

157.5014
156.2028
146.8735
130.1219
128.1095
125.5459
124.3336
123.8928
120.6105
119.1826
117.7308
77.4762
77.1600
76.8390
L5
f1 (ppm)

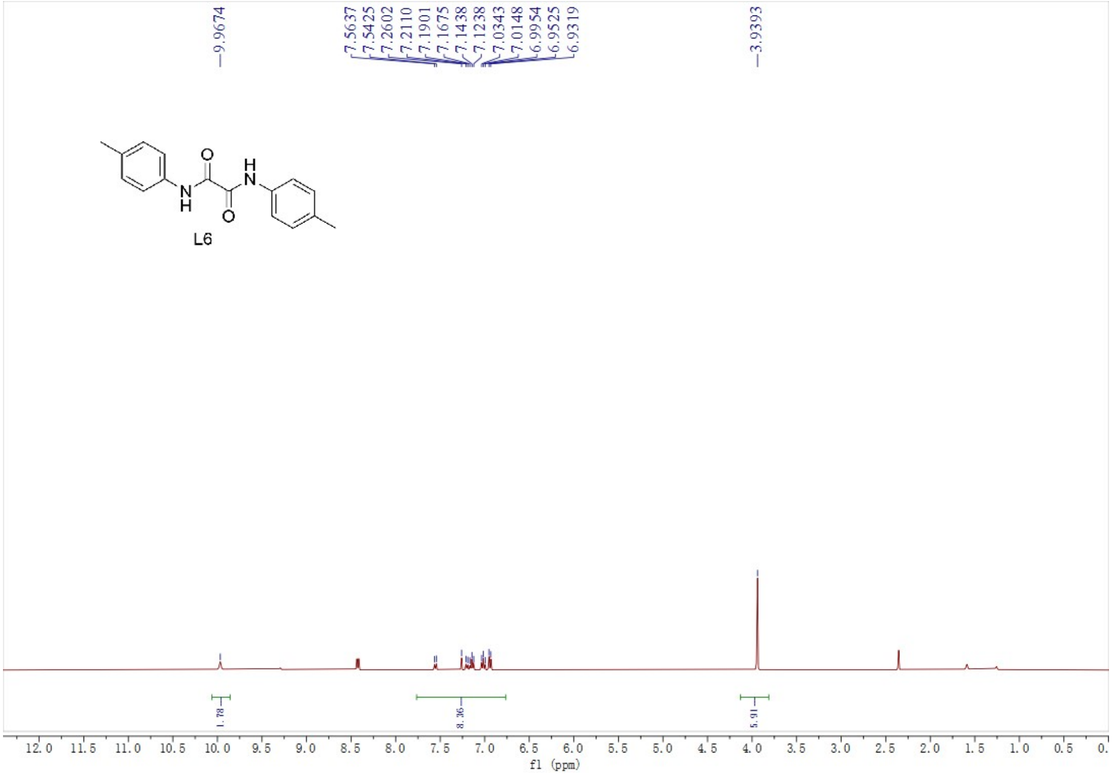

L6
9.9674
7.5637
7.5425
7.2602
7.2110
7.1901
7.1675
7.1438
7.1238
7.0343
7.0148
6.9954
6.9525
6.9319
3.9393
1.78
8.36
5.91
12.0 11.5 11.0 10.5 10.0 9.5 9.0 8.5 8.0 7.5 7.0 6.5 6.0 5.5 5.0 4.5 4.0 3.5 3.0 2.5 2.0 1.5 1.0 0.5 0.
f1 (ppm)

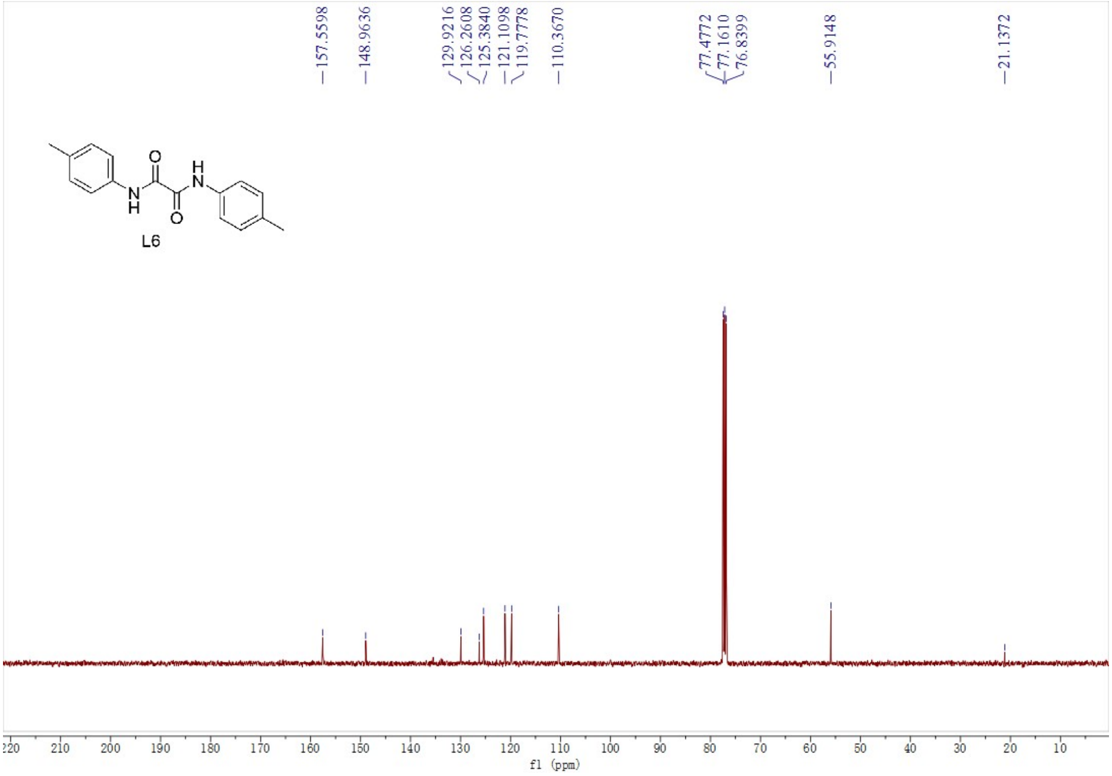

L6
157.5598
148.9636
129.9216
126.2608
125.3840
121.1098
119.7778
110.3670
77.4772
77.1610
76.8399
55.9148
21.1372
220 210 200 190 180 170 160 150 140 130 120 110 100 90 80 70 60 50 40 30 20 10
f1 (ppm)

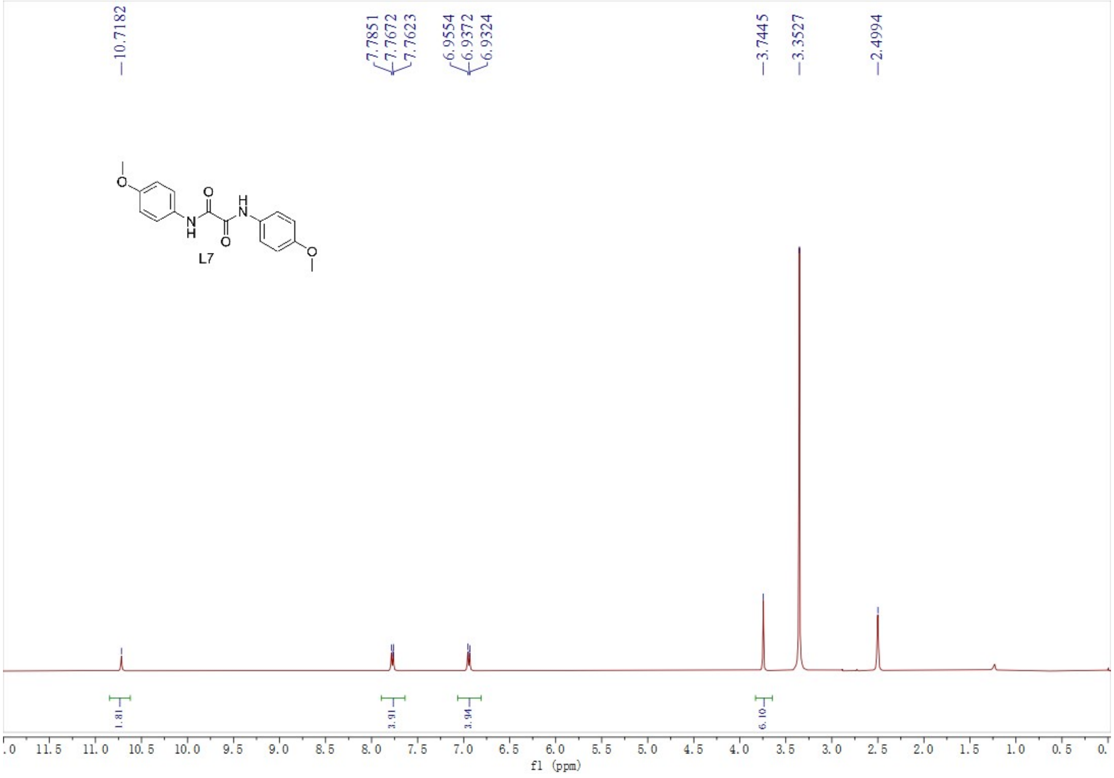

10.7182
7.7851
7.7672
7.7623
6.9554
6.9372
6.9324
3.7445
3.3527
2.4994
L7
1.81
3.91
3.94
6.10
f1 (ppm)

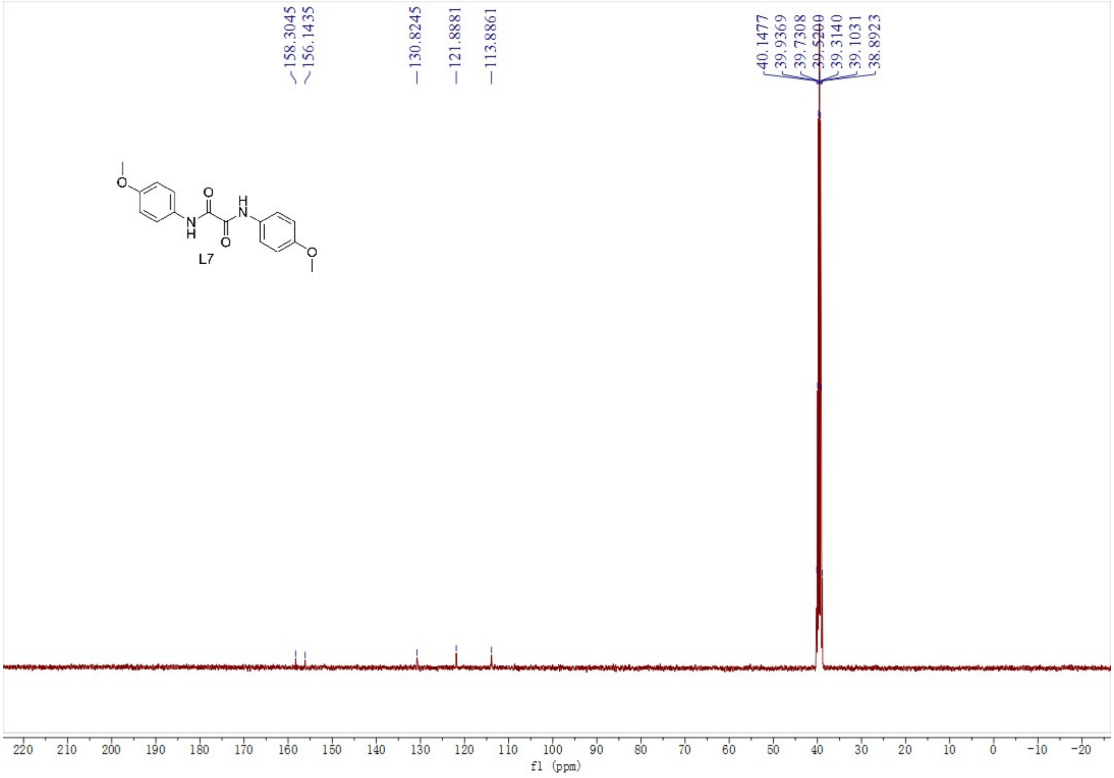

158.3045
156.1435
130.8245
121.8881
113.8861
40.1477
39.9369
39.7308
39.5200
39.3140
39.1031
38.8923
L7
f1 (ppm)

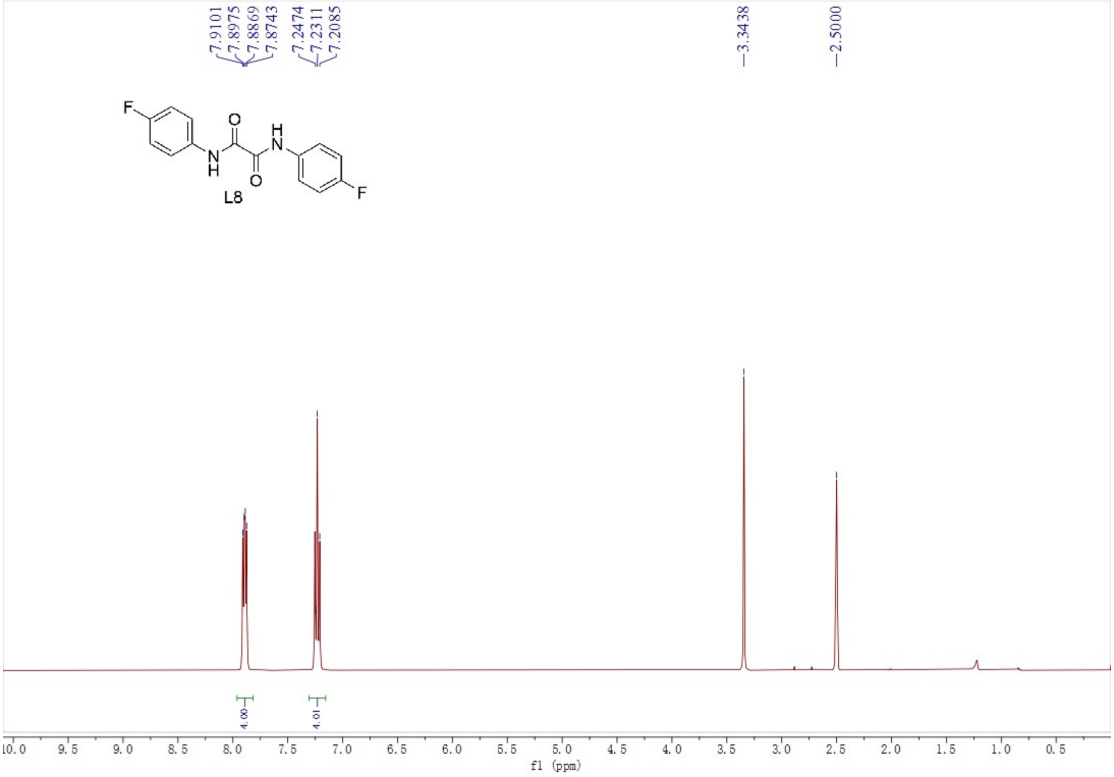
7.9101
7.8975
7.8869
7.8743
7.2474
7.2311
7.2085
3.3438
2.5000
F
O
H
N
N
H
O
F
L8
4.00
4.01
f1 (ppm)

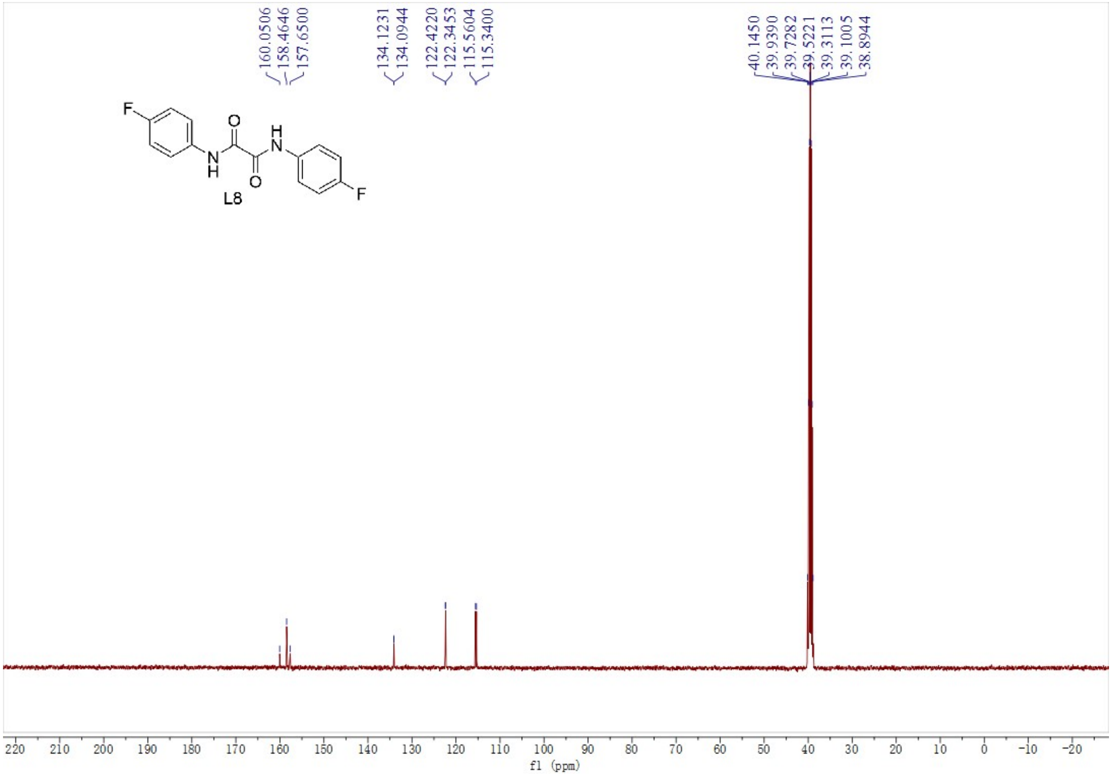
160.0506
158.4646
157.6500
134.1231
134.0944
122.4220
122.3453
115.5604
115.3400
40.1450
39.9390
39.7282
39.5221
39.3113
39.1005
38.8944
F
O
H
N
N
H
O
F
L8
f1 (ppm)

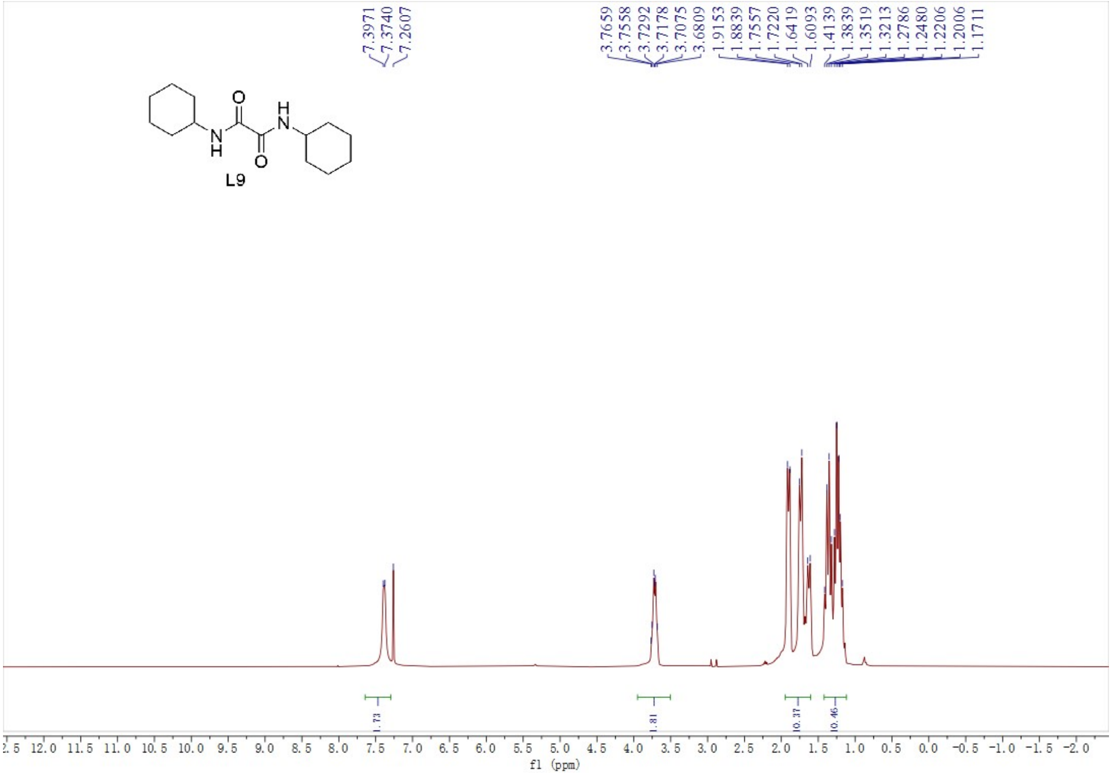
7.3971
7.3740
7.2607
3.7659
3.7558
3.7292
3.7178
3.7075
3.6809
1.9153
1.8839
1.7557
1.7220
1.6419
1.6093
1.4139
1.3839
1.3519
1.3213
1.2786
1.2480
1.2206
1.2006
1.1711
L9
1.73
1.81
10.37
10.46
fl (ppm)

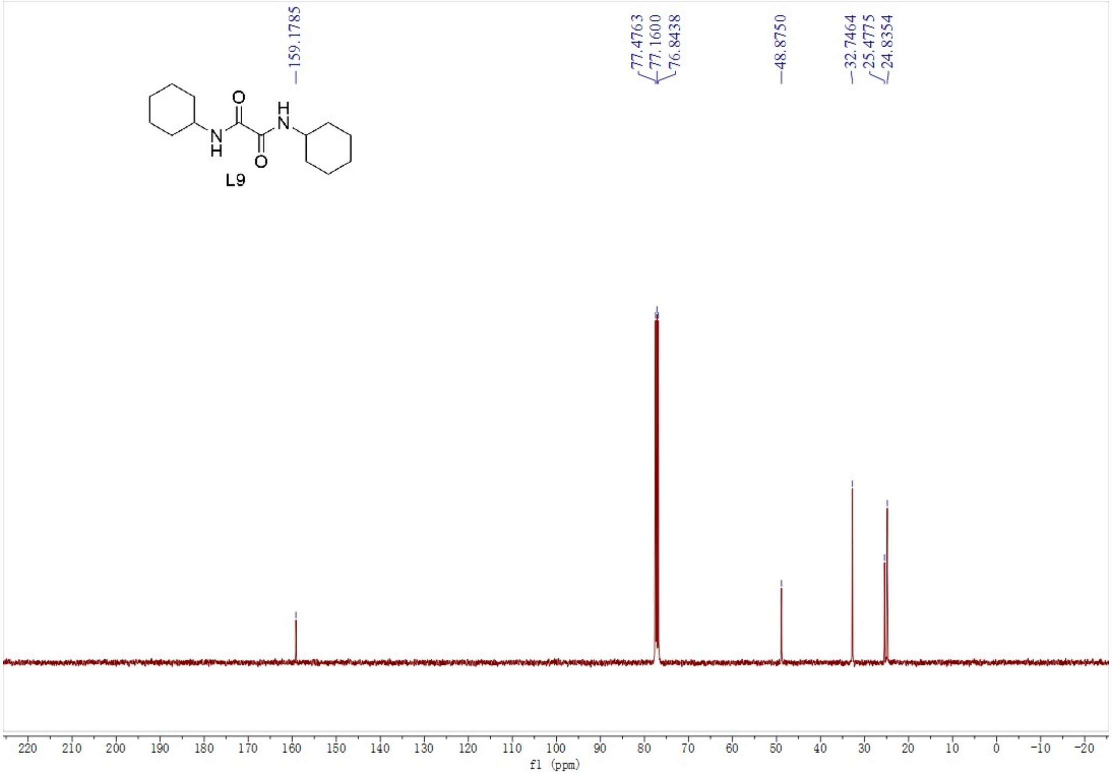
159.1785
77.4763
77.1600
76.8438
48.8750
32.7464
25.4775
24.8354
L9
fl (ppm)

…

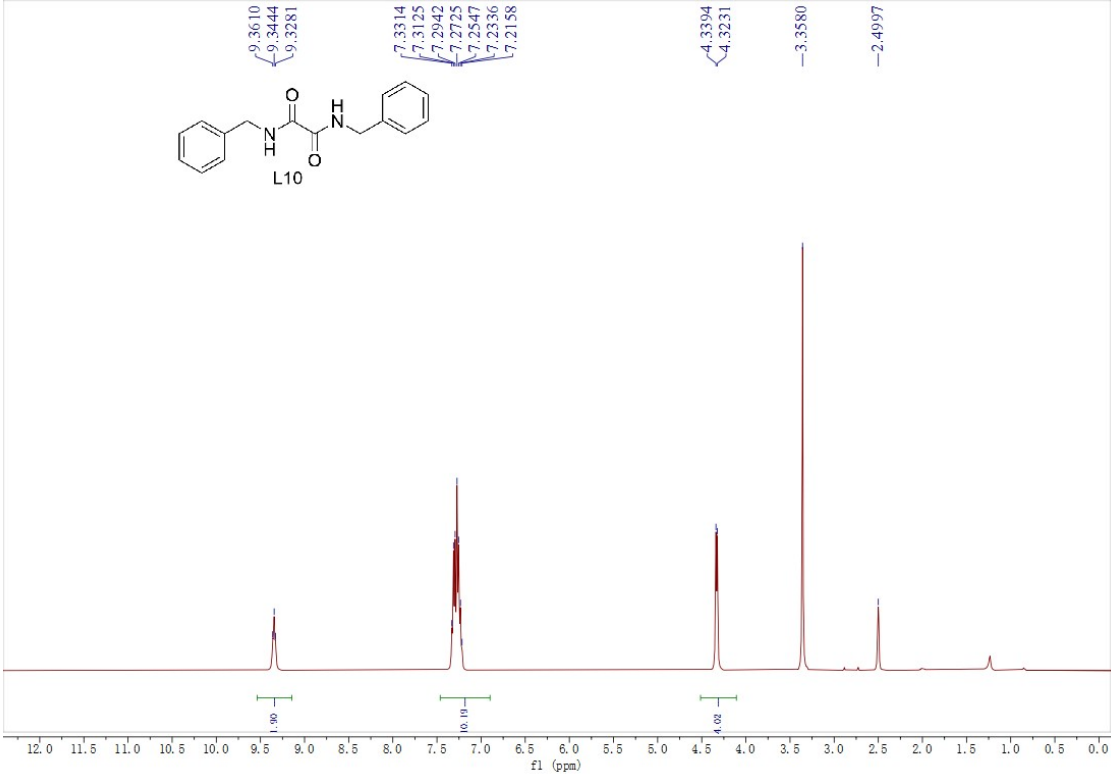
9.3610
9.3444
9.3281
7.3314
7.3125
7.2942
7.2725
7.2547
7.2336
7.2158
4.3394
4.3231
3.3580
2.4997
L10
1.90
10.19
4.02
12.0 11.5 11.0 10.5 10.0 9.5 9.0 8.5 8.0 7.5 7.0 6.5 6.0 5.5 5.0 4.5 4.0 3.5 3.0 2.5 2.0 1.5 1.0 0.5 0.0
f1 (ppm)

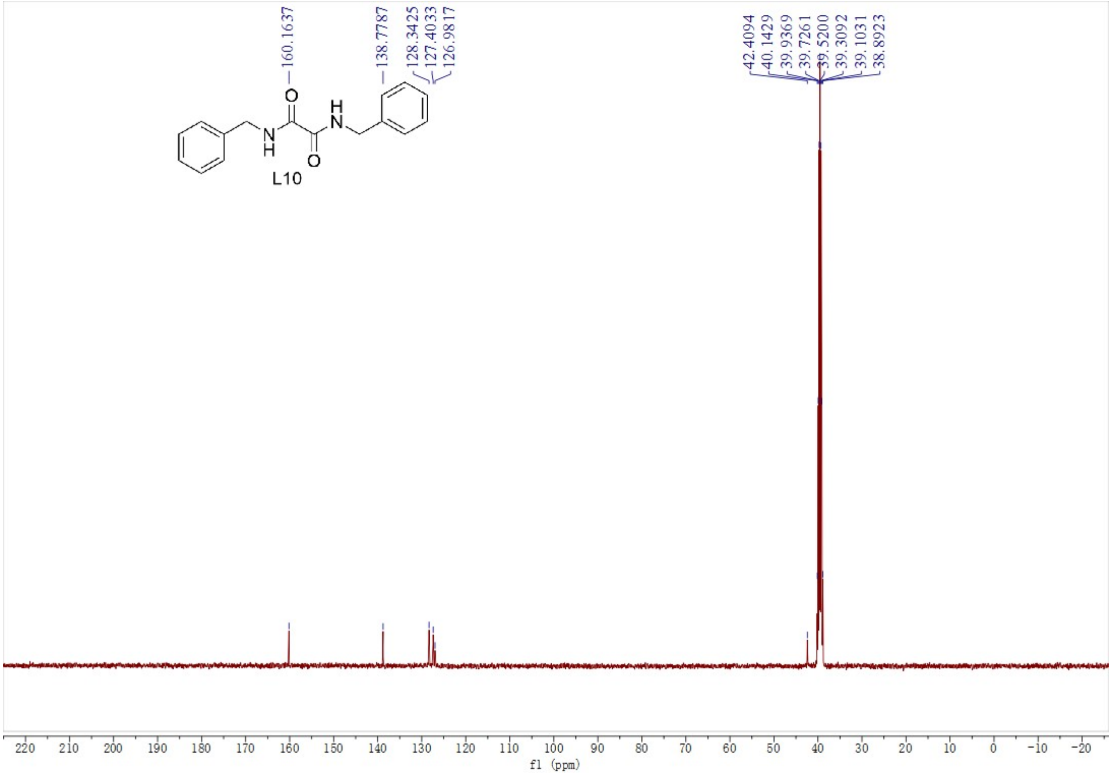
160.1637
138.7787
128.3425
127.4033
126.9817
42.4094
40.1429
39.9369
39.7261
39.5200
39.3092
39.1031
38.8923
L10
220 210 200 190 180 170 160 150 140 130 120 110 100 90 80 70 60 50 40 30 20 10 0 -10 -20
f1 (ppm)

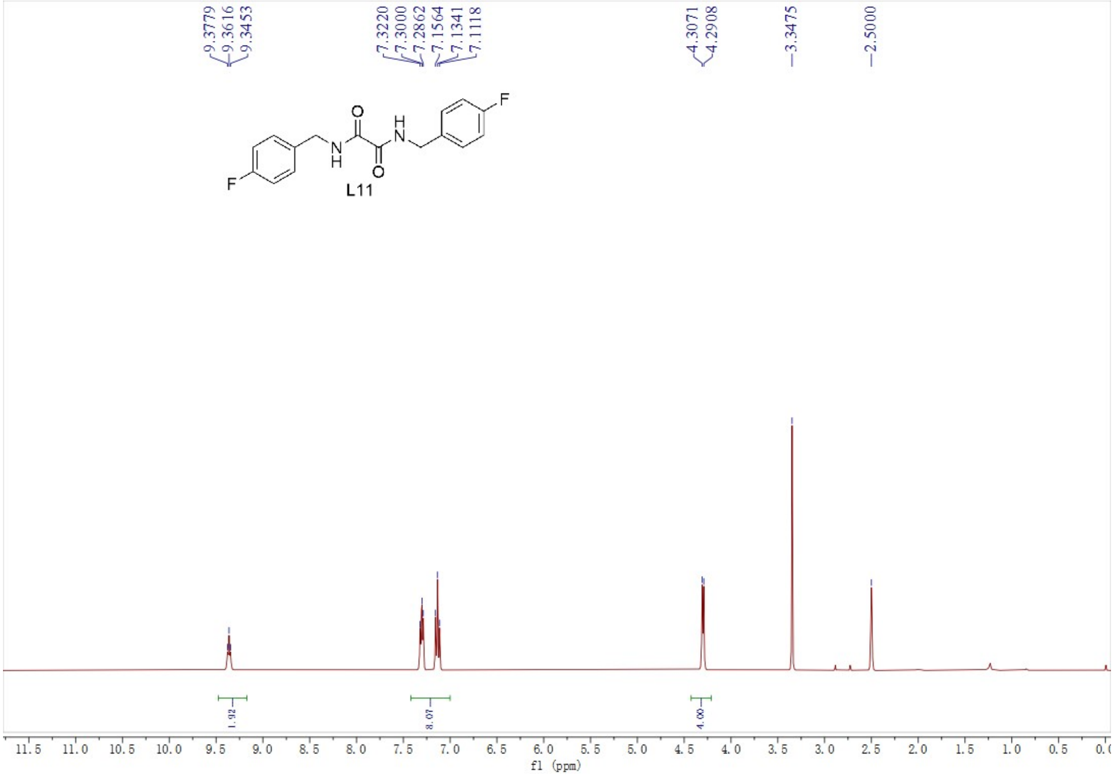
9.3779
9.3616
9.3453
7.3220
7.3000
7.2862
7.1564
7.1341
7.1118
4.3071
4.2908
3.3475
2.5000
L11
1.92
8.07
4.00
11.5 11.0 10.5 10.0 9.5 9.0 8.5 8.0 7.5 7.0 6.5 6.0 5.5 5.0 4.5 4.0 3.5 3.0 2.5 2.0 1.5 1.0 0.5 0.0
f1 (ppm)

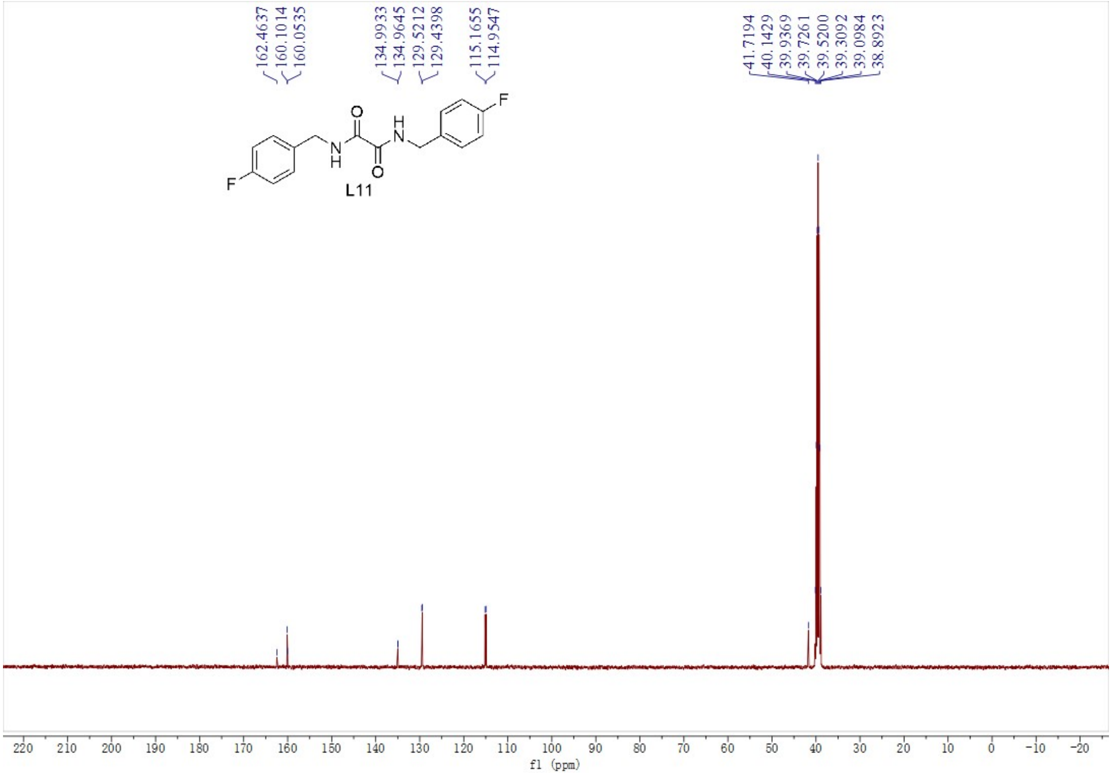
162.4637
160.1014
160.0535
134.9933
134.9645
129.5212
129.4398
115.1655
114.9547
41.7194
40.1429
39.9369
39.7261
39.5200
39.3092
39.0984
38.8923
L11
220 210 200 190 180 170 160 150 140 130 120 110 100 90 80 70 60 50 40 30 20 10 0 -10 -20
f1 (ppm)

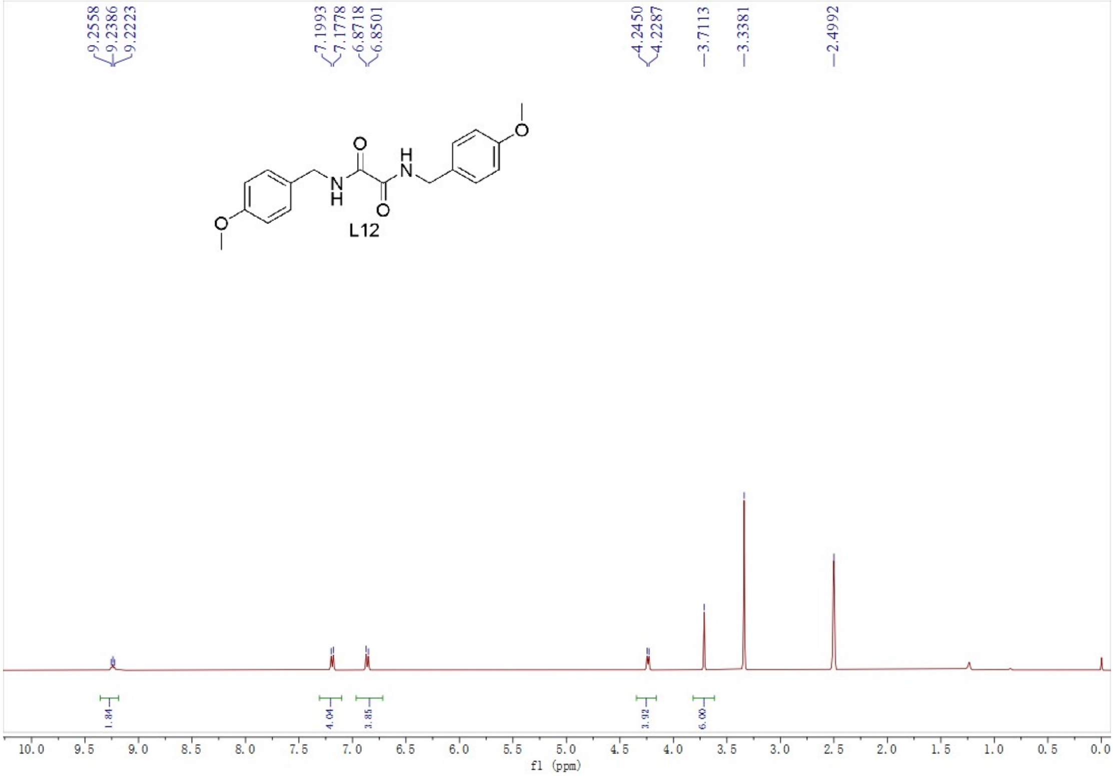
9.2558
9.2386
9.2223
7.1993
7.1778
6.8718
6.8501
4.2450
4.2287
3.7113
3.3381
2.4992
L12
1.84
4.04
3.85
3.92
6.00
10.0 9.5 9.0 8.5 8.0 7.5 7.0 6.5 6.0 5.5 5.0 4.5 4.0 3.5 3.0 2.5 2.0 1.5 1.0 0.5 0.0
f1 (ppm)

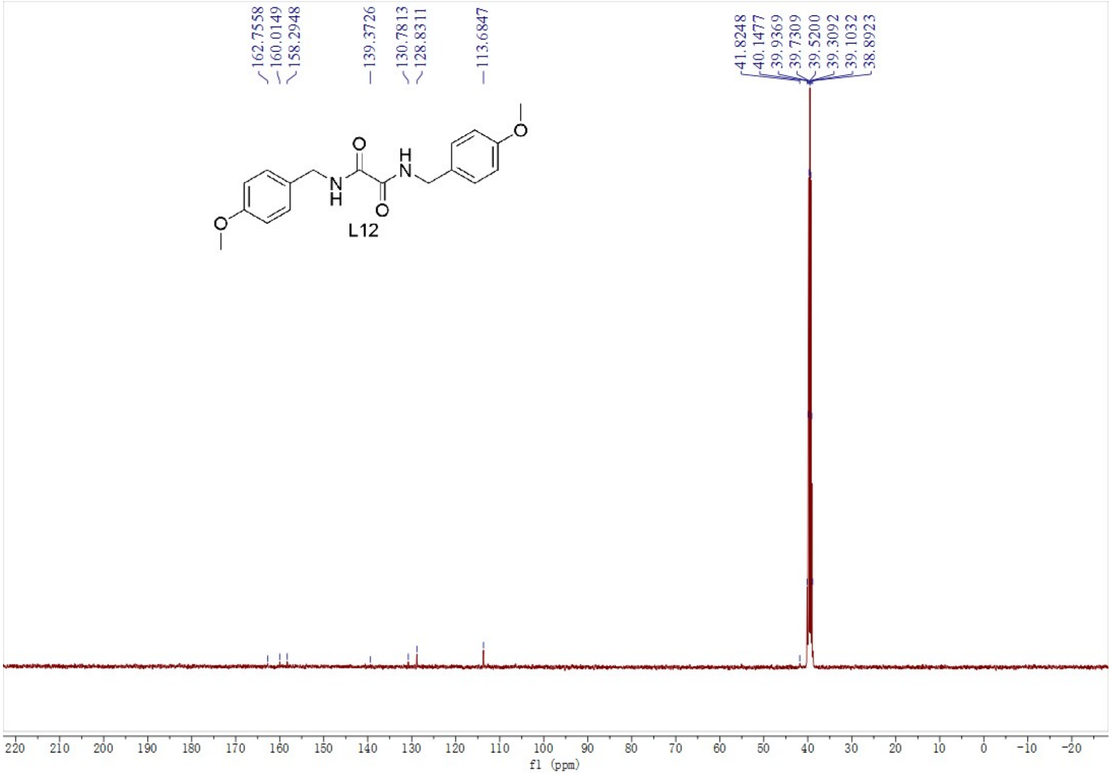
162.7558
160.0149
158.2948
139.3726
130.7813
128.8311
113.6847
41.8248
40.1477
39.9369
39.7309
39.5200
39.3092
39.1032
38.8923
L12
220 210 200 190 180 170 160 150 140 130 120 110 100 90 80 70 60 50 40 30 20 10 0 -10 -20
f1 (ppm)

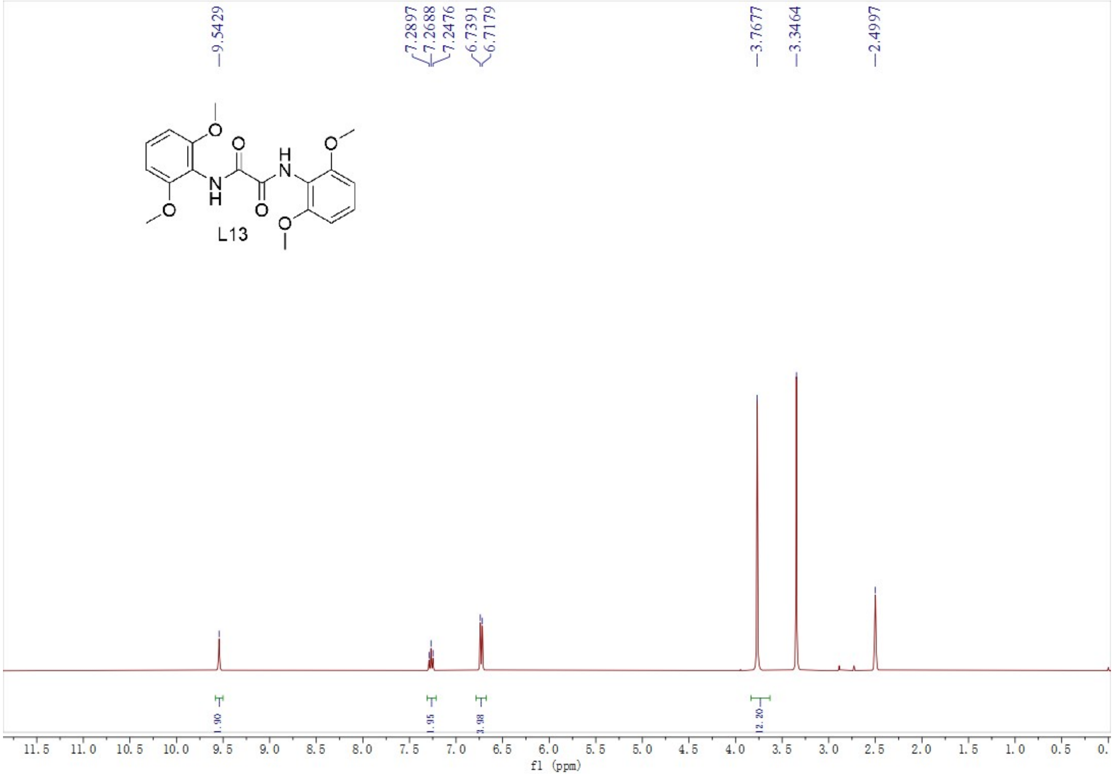
9.5429
7.2897
7.2688
7.2476
6.7391
6.7179
3.7677
3.3464
2.4997
L13
1.90
1.95
3.98
12.20
f1 (ppm)

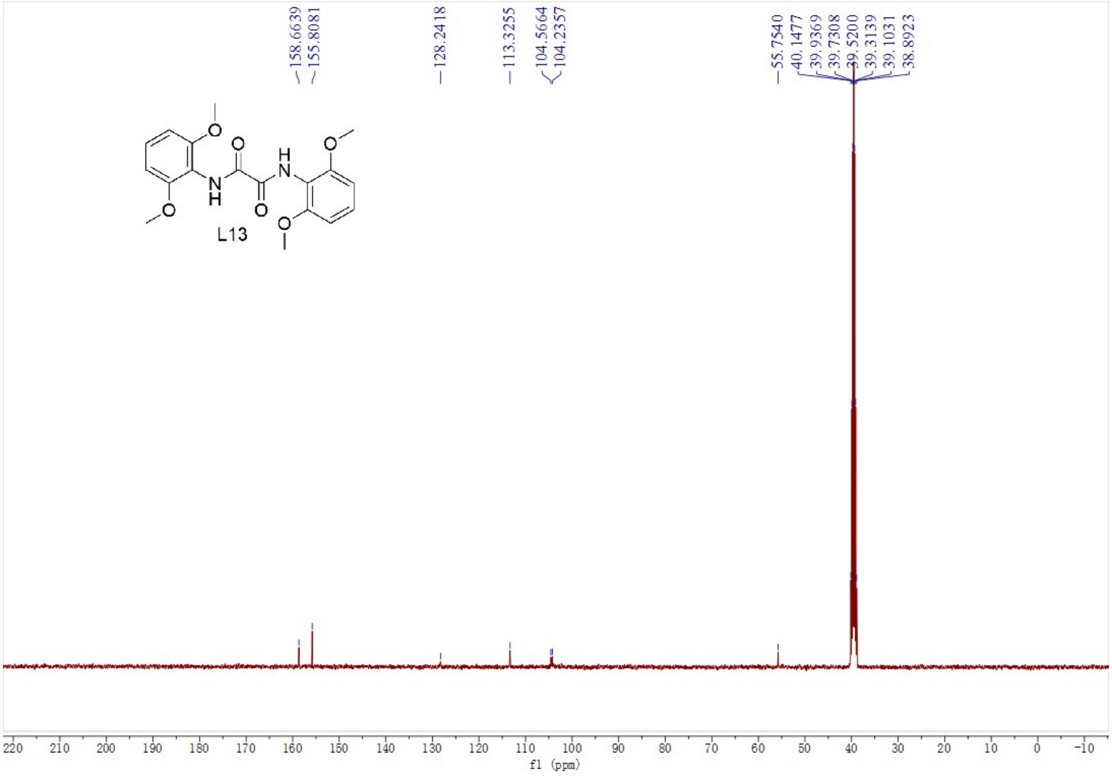
158.6639
155.8081
128.2418
113.3255
104.5664
104.2357
55.7540
40.1477
39.9369
39.7308
39.5200
39.3139
39.1031
38.8923
L13
f1 (ppm)

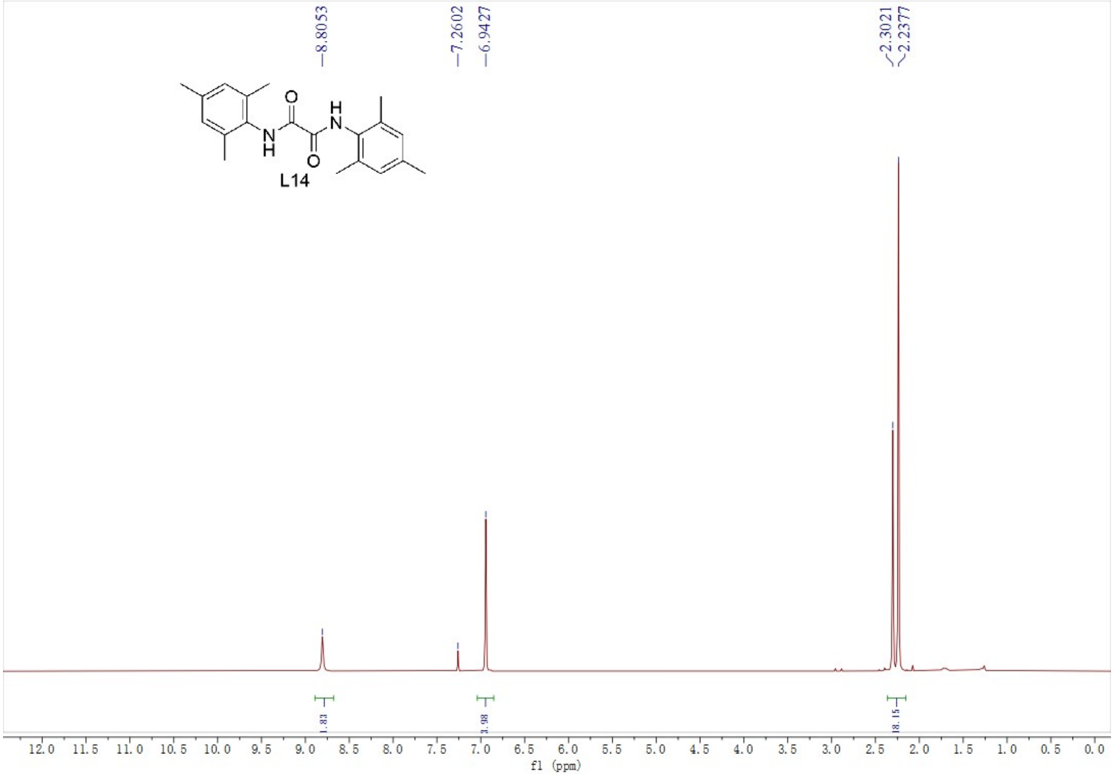
8.8053
7.2602
6.9427
2.3021
2.2377
L14
1.83
3.98
18.15
12.0 11.5 11.0 10.5 10.0 9.5 9.0 8.5 8.0 7.5 7.0 6.5 6.0 5.5 5.0 4.5 4.0 3.5 3.0 2.5 2.0 1.5 1.0 0.5 0.0
f1 (ppm)

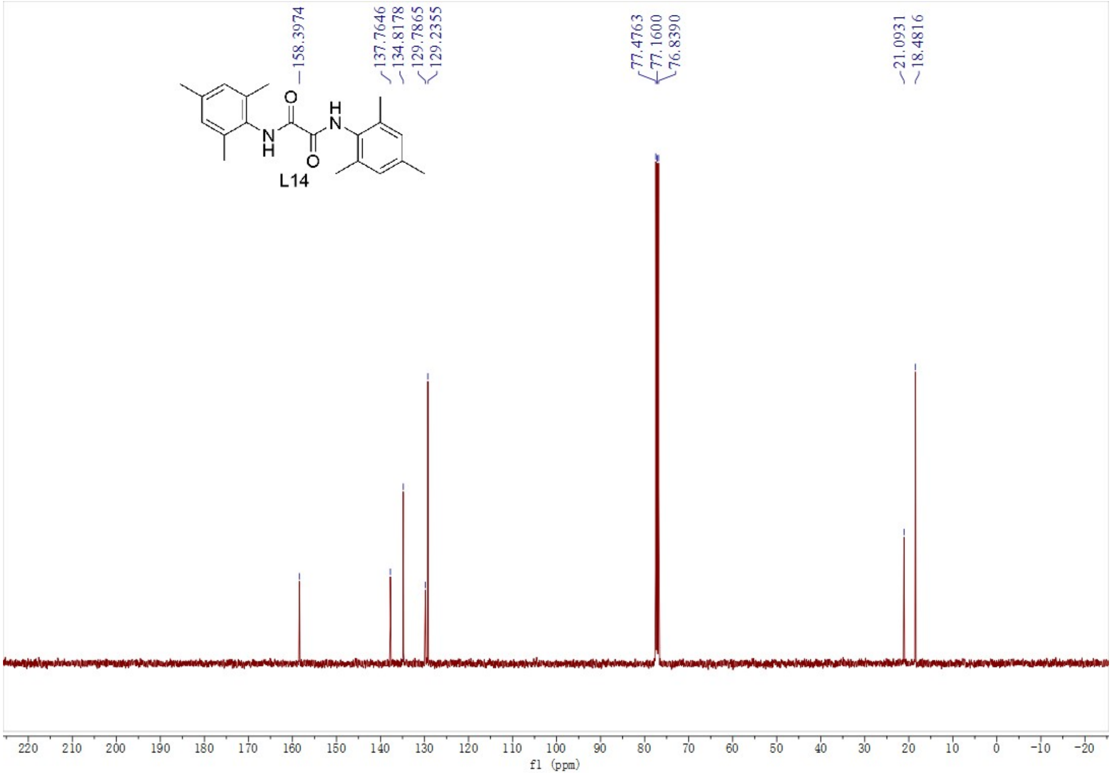
158.3974
137.7646
134.8178
129.7865
129.2355
77.4763
77.1600
76.8390
21.0931
18.4816
L14
220 210 200 190 180 170 160 150 140 130 120 110 100 90 80 70 60 50 40 30 20 10 0 -10 -20
f1 (ppm)

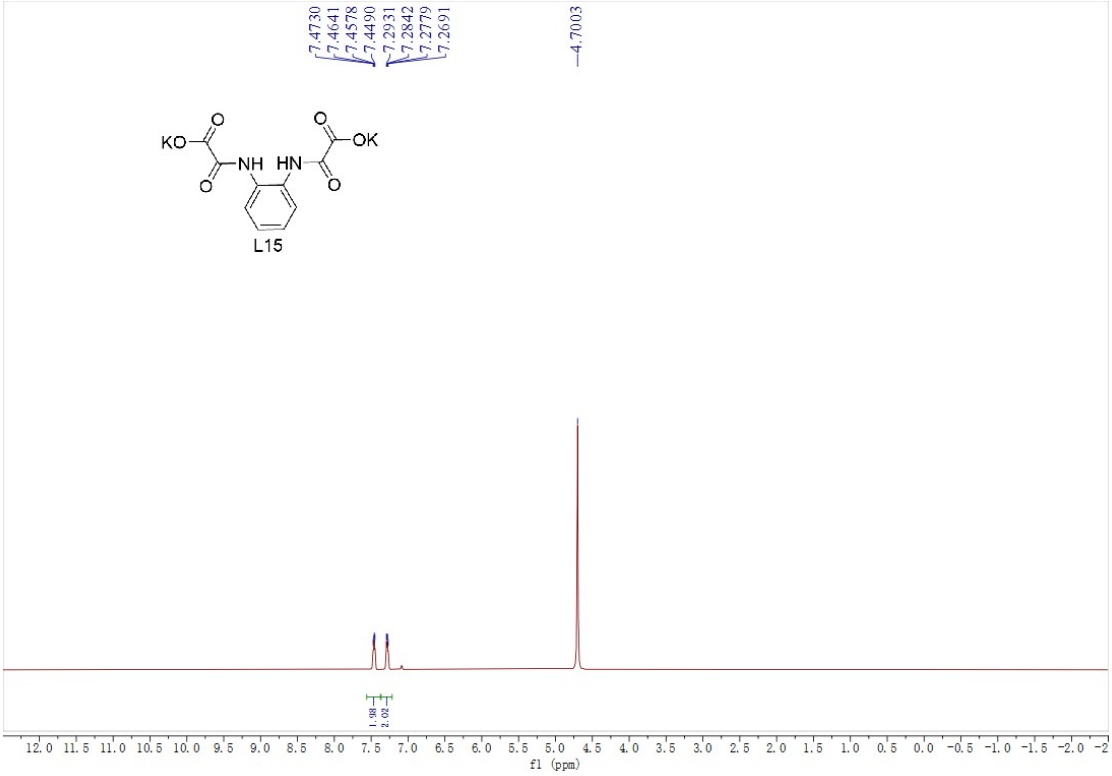

7.4730
7.4641
7.4578
7.4490
7.2931
7.2842
7.2779
7.2691
4.7003
KO
O
O
NH
HN
O
O
OK
L15
1.98
2.02
12.0 11.5 11.0 10.5 10.0 9.5 9.0 8.5 8.0 7.5 7.0 6.5 6.0 5.5 5.0 4.5 4.0 3.5 3.0 2.5 2.0 1.5 1.0 0.5 0.0 −0.5 −1.0 −1.5 −2.0
f1 (ppm)

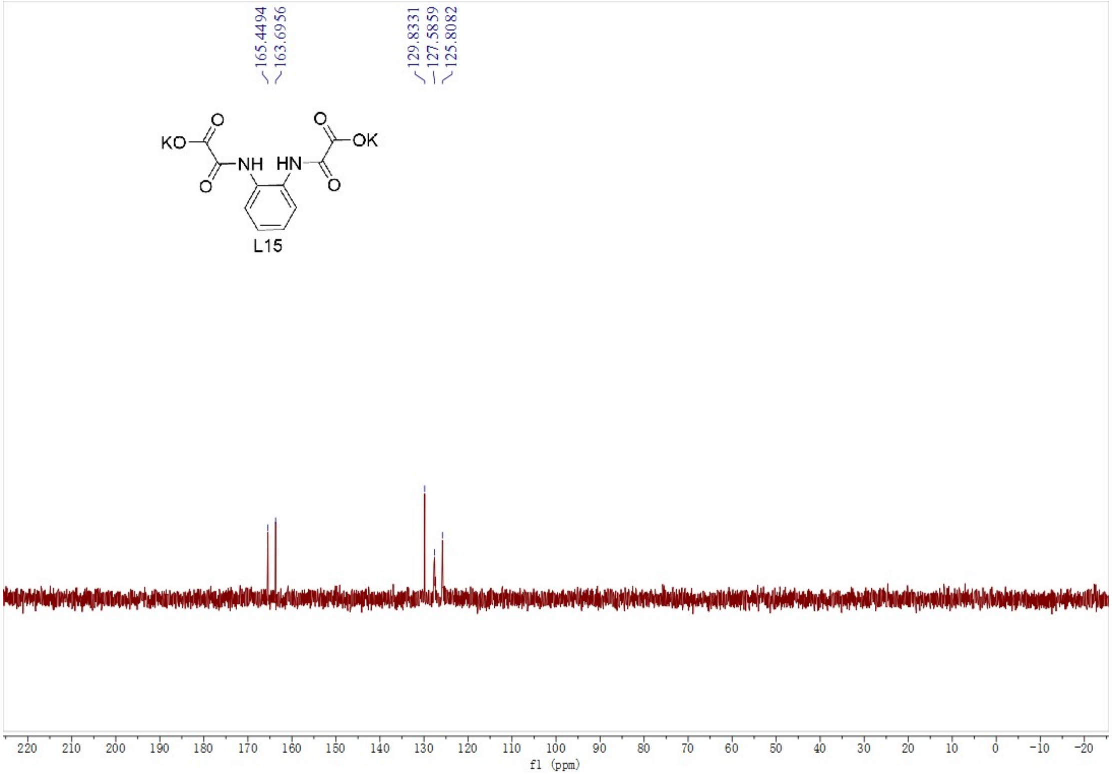

165.4494
163.6956
129.8331
127.5859
125.8082
KO
O
O
NH
HN
O
O
OK
L15
220 210 200 190 180 170 160 150 140 130 120 110 100 90 80 70 60 50 40 30 20 10 0 −10 −20
f1 (ppm)

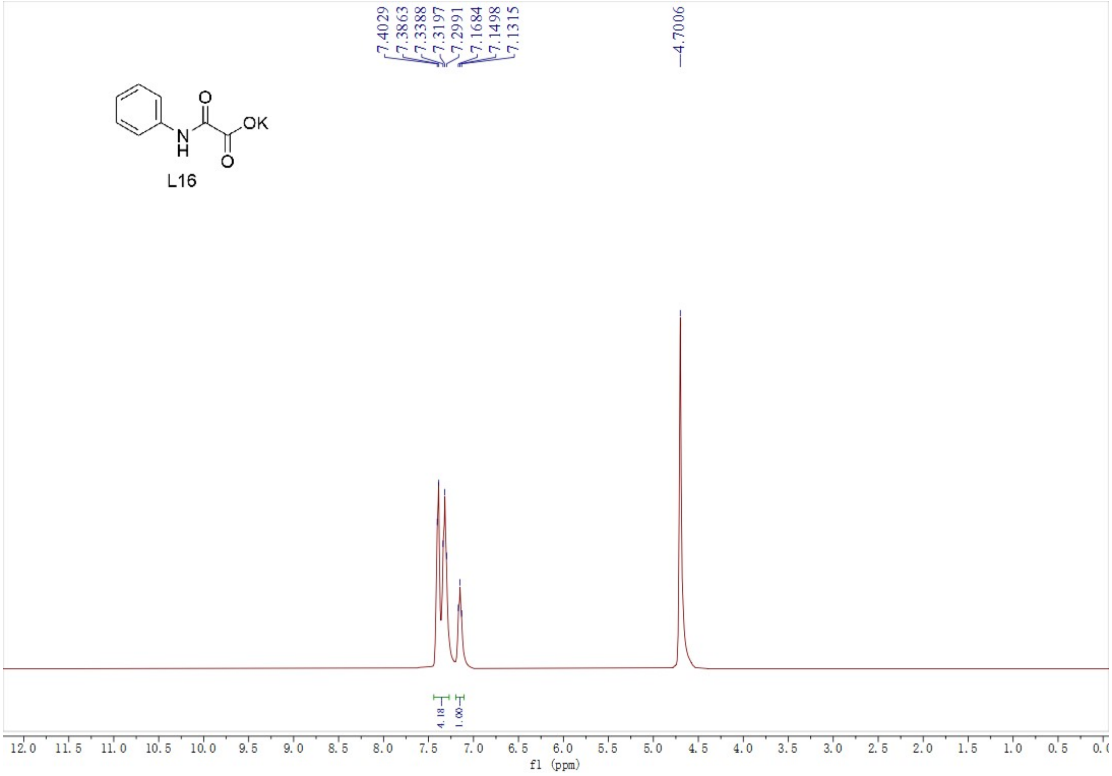
7.4029
7.3863
7.3388
7.3197
7.2991
7.1684
7.1498
7.1315
4.7006
O
OK
N
H
O
L16
4.18
1.00
12.0 11.5 11.0 10.5 10.0 9.5 9.0 8.5 8.0 7.5 7.0 6.5 6.0 5.5 5.0 4.5 4.0 3.5 3.0 2.5 2.0 1.5 1.0 0.5
f1 (ppm)

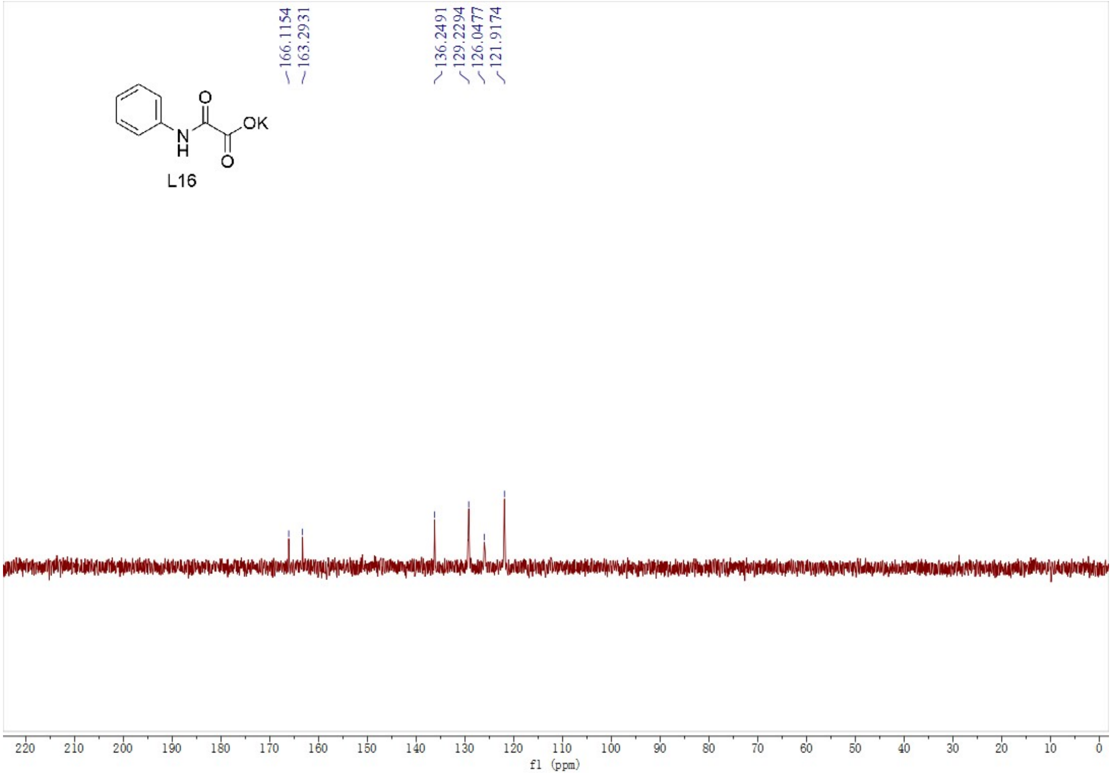
166.1154
163.2931
136.2491
129.2294
126.0477
121.9174
O
OK
N
H
O
L16
220 210 200 190 180 170 160 150 140 130 120 110 100 90 80 70 60 50 40 30 20 10 0
f1 (ppm)

…

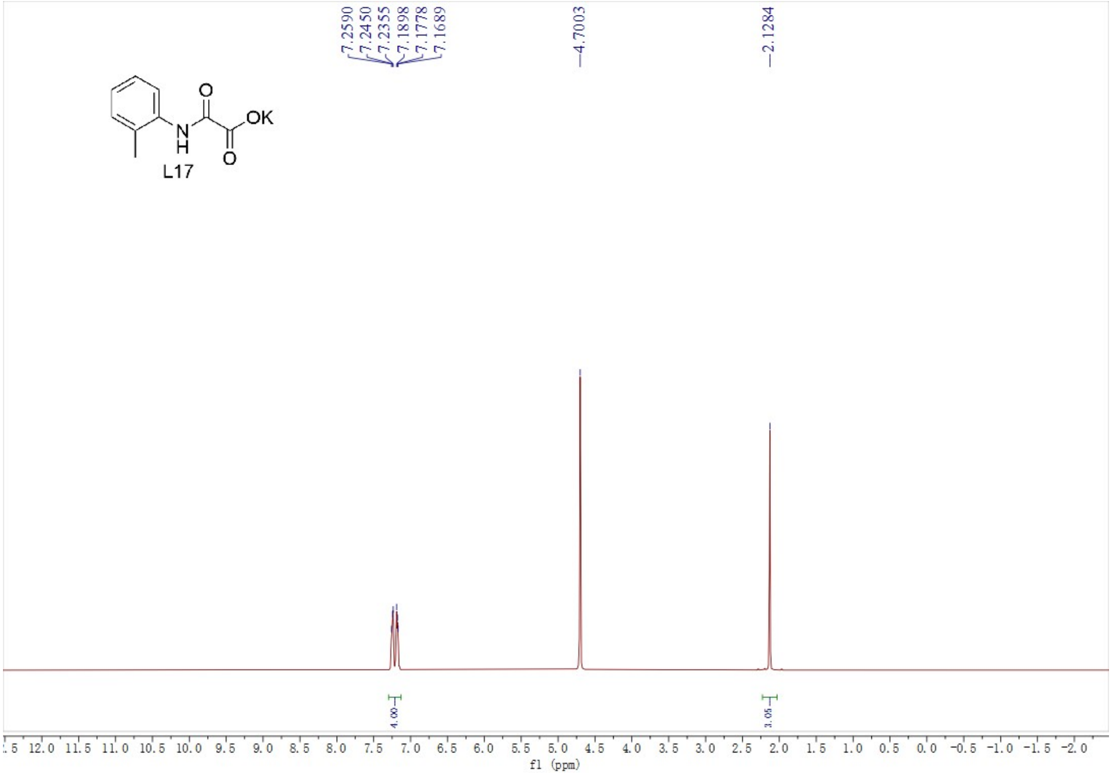
7.2590
7.2450
7.2355
7.1898
7.1778
7.1689
4.7003
2.1284
OK
N
H
O
O
L17
4.00
3.05
fl (ppm)

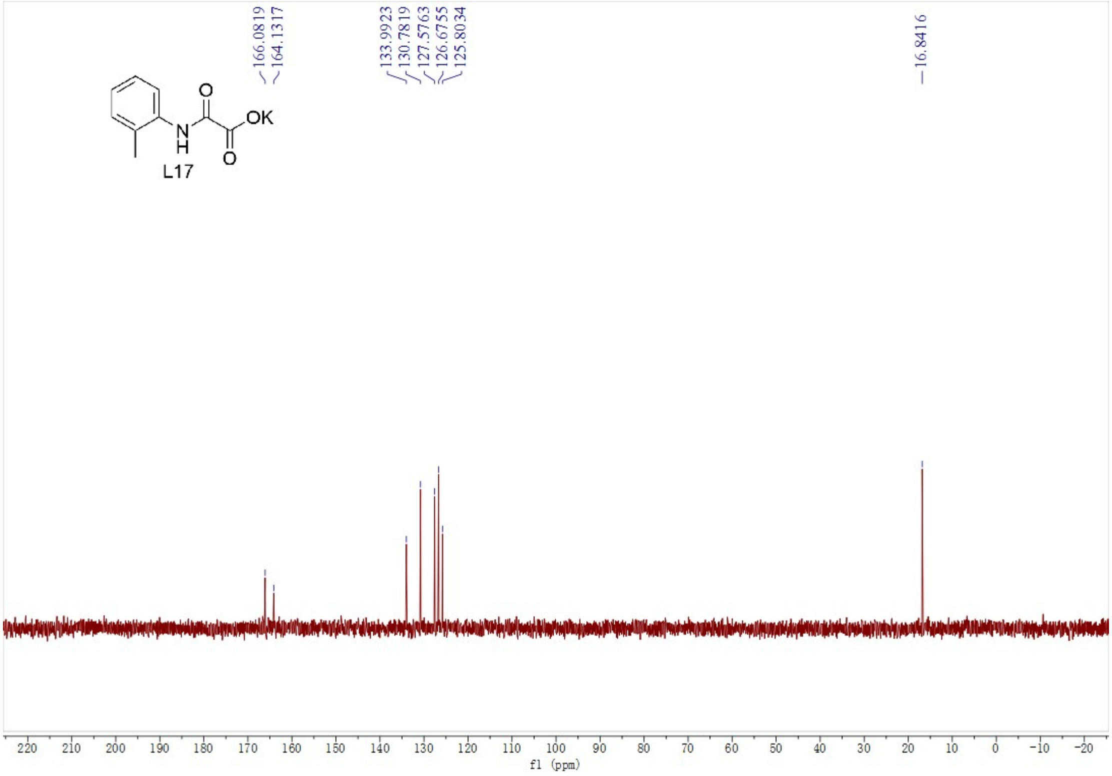
166.0819
164.1317
133.9923
130.7819
127.5763
126.6755
125.8034
16.8416
OK
N
H
O
O
L17
fl (ppm)

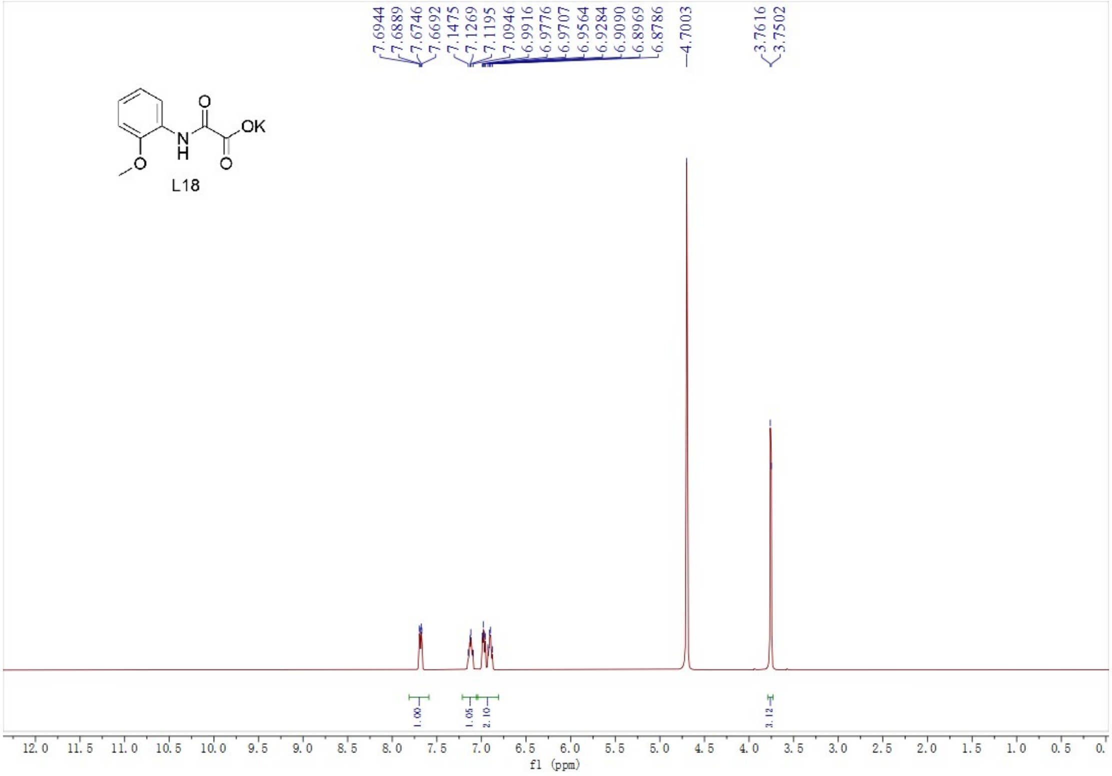
7.6944
7.6889
7.6746
7.6692
7.1475
7.1269
7.1195
7.0946
6.9916
6.9776
6.9707
6.9564
6.9284
6.9090
6.8969
6.8786
4.7003
3.7616
3.7502
OK
N
H
O
L18
1.00
1.05
2.10
3.12
12.0 11.5 11.0 10.5 10.0 9.5 9.0 8.5 8.0 7.5 7.0 6.5 6.0 5.5 5.0 4.5 4.0 3.5 3.0 2.5 2.0 1.5 1.0 0.5 0.
f1 (ppm)

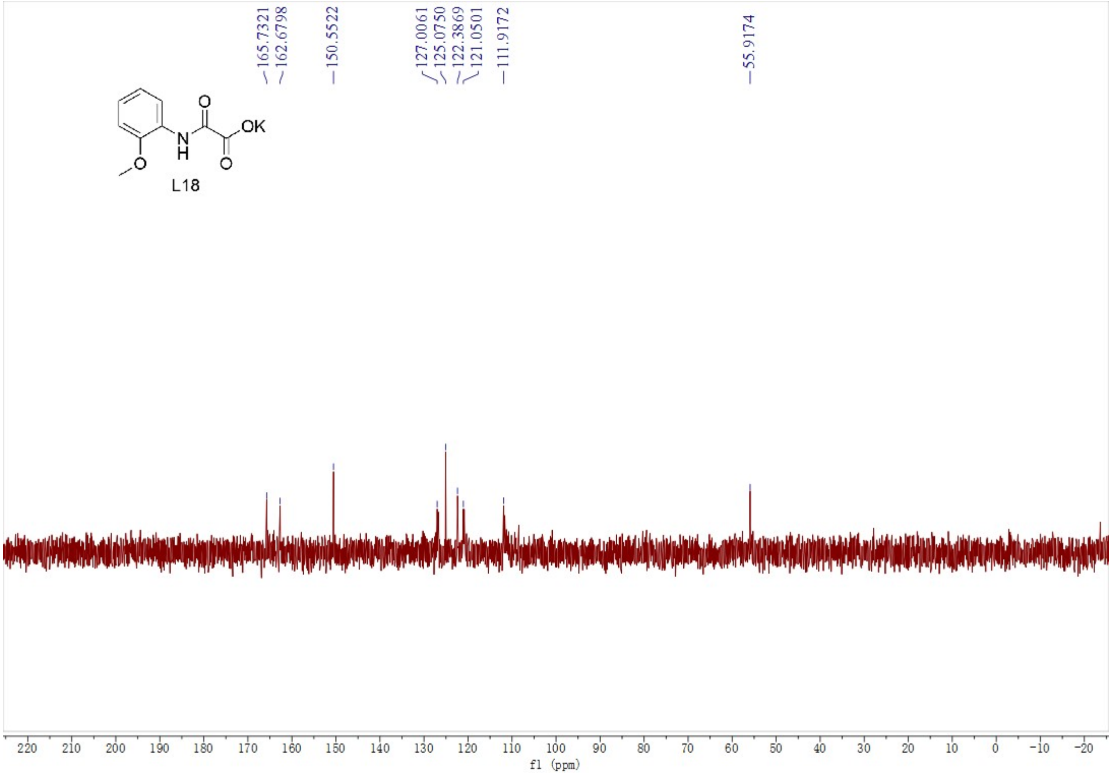
165.7321
162.6798
150.5522
127.0061
125.0750
122.3869
121.0501
111.9172
55.9174
OK
N
H
O
L18
220 210 200 190 180 170 160 150 140 130 120 110 100 90 80 70 60 50 40 30 20 10 0 -10 -20
f1 (ppm)

…

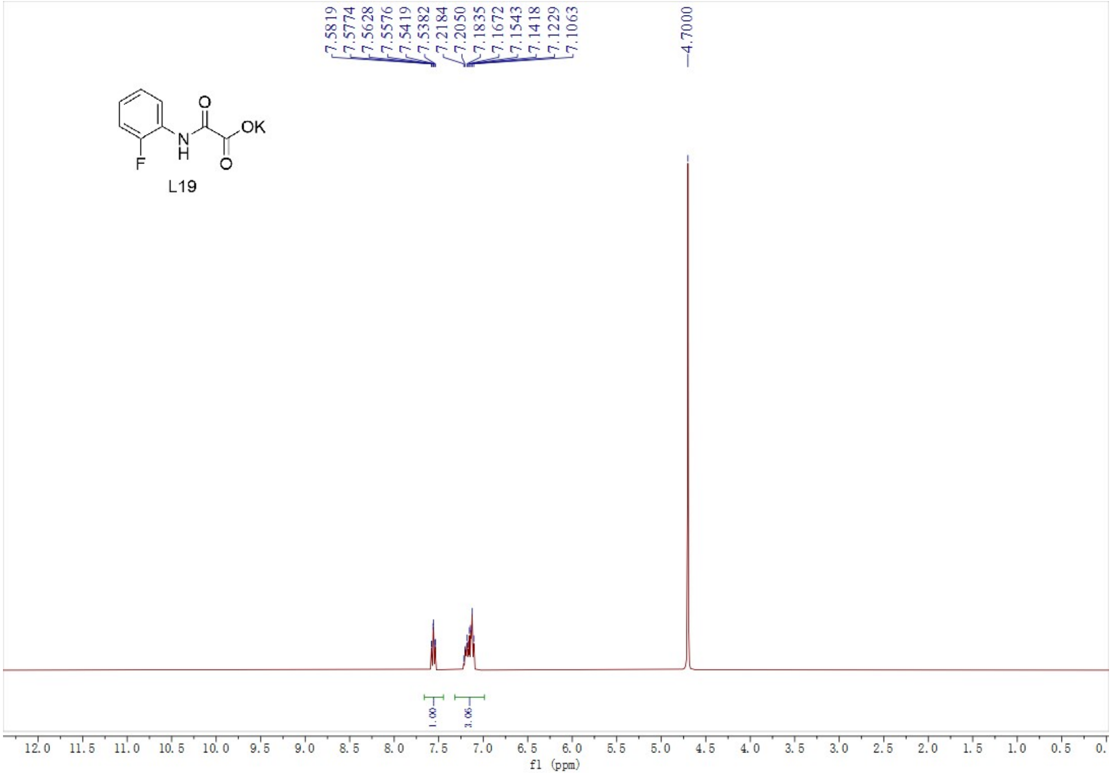
7.5819
7.5774
7.5628
7.5576
7.5419
7.5382
7.2184
7.2050
7.1835
7.1672
7.1543
7.1418
7.1229
7.1063
4.7000
OK
N
H
O
O
F
L19
1.00
3.06
12.0 11.5 11.0 10.5 10.0 9.5 9.0 8.5 8.0 7.5 7.0 6.5 6.0 5.5 5.0 4.5 4.0 3.5 3.0 2.5 2.0 1.5 1.0 0.5 0.
f1 (ppm)

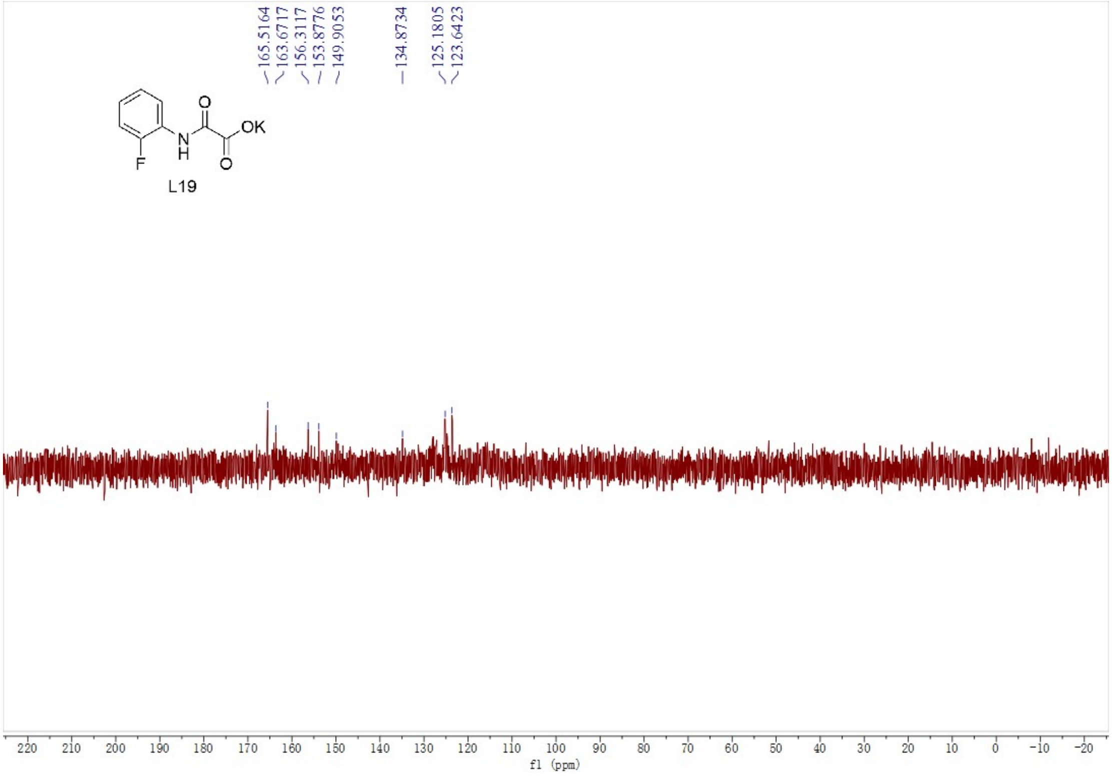
165.5164
163.6717
156.3117
153.8776
149.9053
134.8734
125.1805
123.6423
OK
N
H
O
O
F
L19
220 210 200 190 180 170 160 150 140 130 120 110 100 90 80 70 60 50 40 30 20 10 0 -10 -20
f1 (ppm)

…

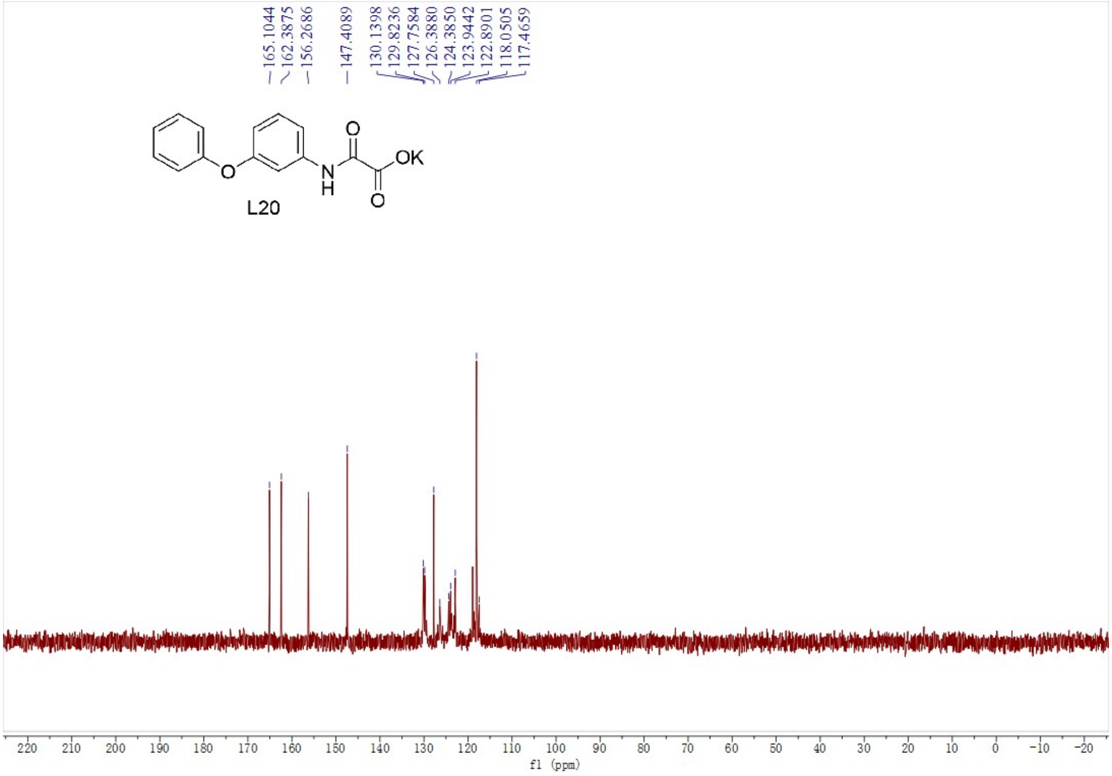
165.1044
162.3875
156.2686
147.4089
130.1398
129.8236
127.7584
126.3880
124.3850
123.9442
122.8901
118.0505
117.4659
OK
N
H
O
O
O
L20
220 210 200 190 180 170 160 150 140 130 120 110 100 90 80 70 60 50 40 30 20 10 0 -10 -20
f1 (ppm)

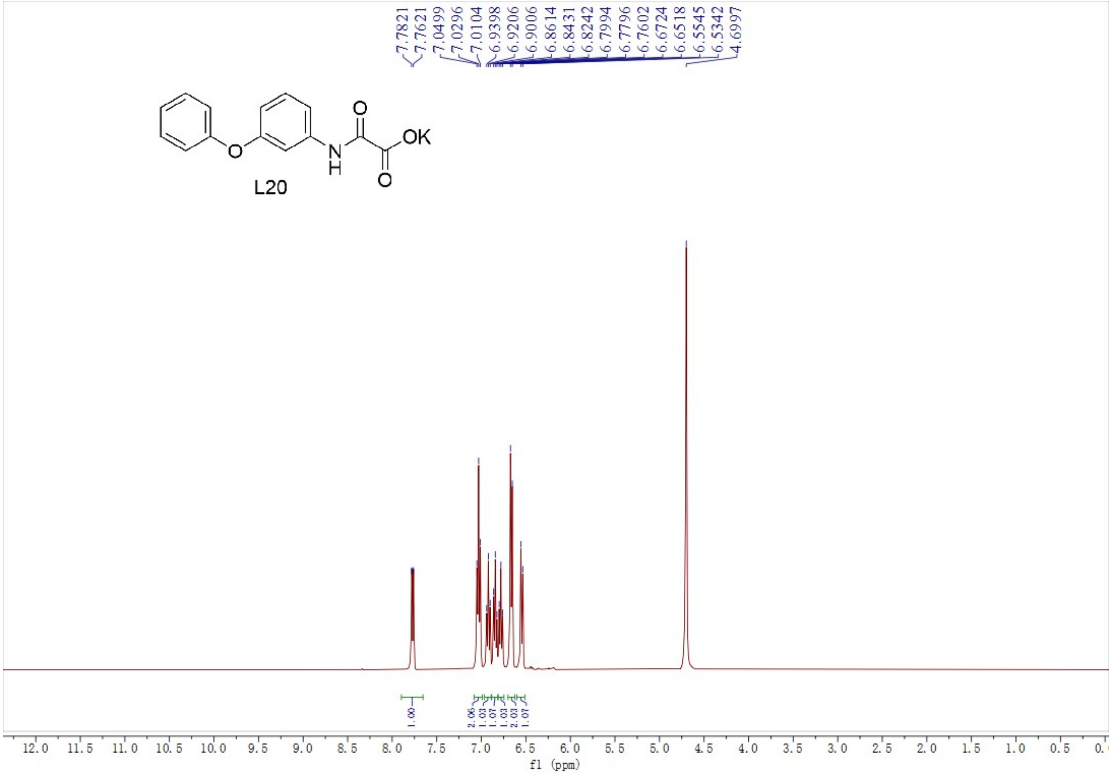
7.7821
7.7621
7.0499
7.0296
7.0104
6.9398
6.9206
6.9006
6.8614
6.8431
6.8242
6.7994
6.7796
6.7602
6.6724
6.6518
6.5545
6.5342
4.6997
OK
N
H
O
O
O
L20
1.00
12.0 11.5 11.0 10.5 10.0 9.5 9.0 8.5 8.0 7.5 7.0 6.5 6.0 5.5 5.0 4.5 4.0 3.5 3.0 2.5 2.0 1.5 1.0 0.5
f1 (ppm)

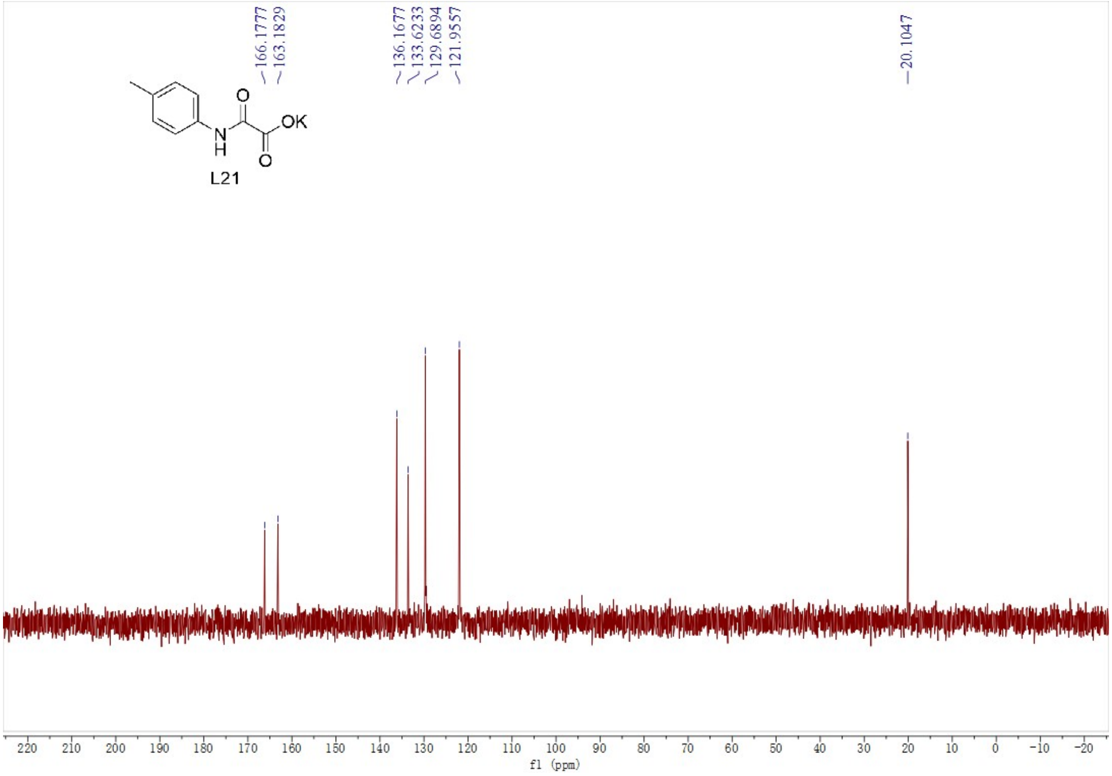
166.1777
163.1829
136.1677
133.6233
129.6894
121.9557
20.1047
O
N
H
OK
O
L21
220 210 200 190 180 170 160 150 140 130 120 110 100 90 80 70 60 50 40 30 20 10 0 -10 -20
f1 (ppm)

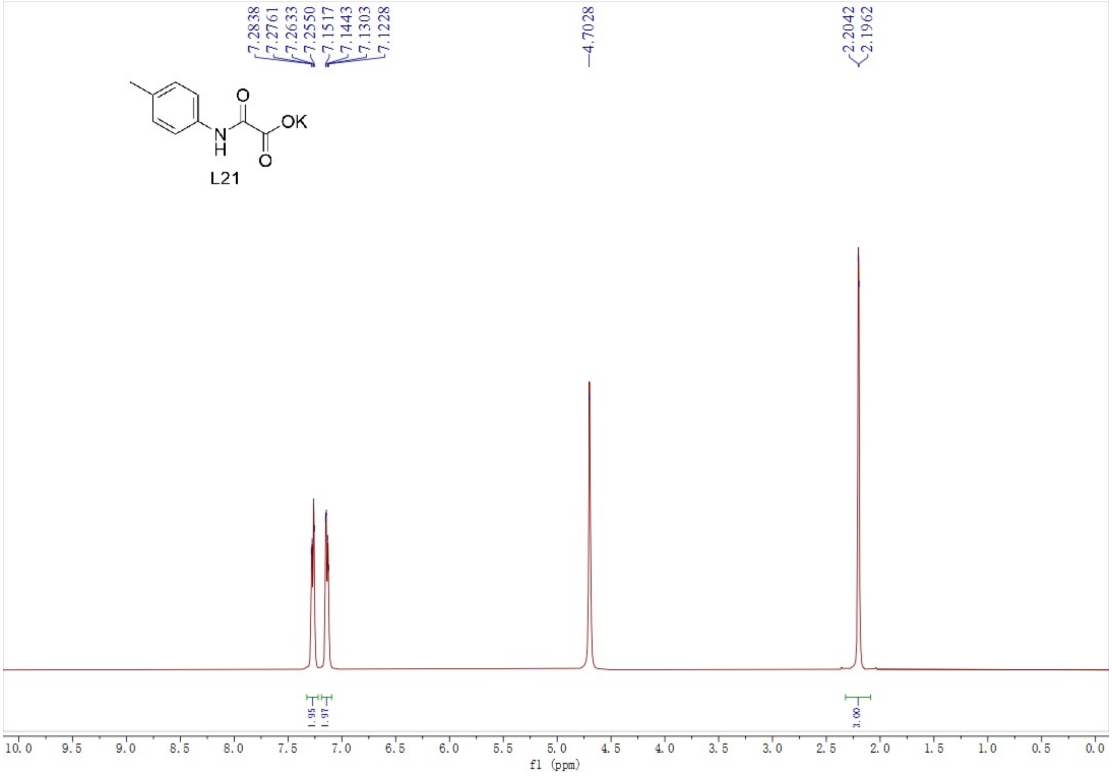
7.2838
7.2761
7.2633
7.2550
7.1517
7.1443
7.1303
7.1228
4.7028
2.2042
2.1962
O
N
H
OK
O
L21
1.95
1.97
3.00
10.0 9.5 9.0 8.5 8.0 7.5 7.0 6.5 6.0 5.5 5.0 4.5 4.0 3.5 3.0 2.5 2.0 1.5 1.0 0.5 0.0
f1 (ppm)

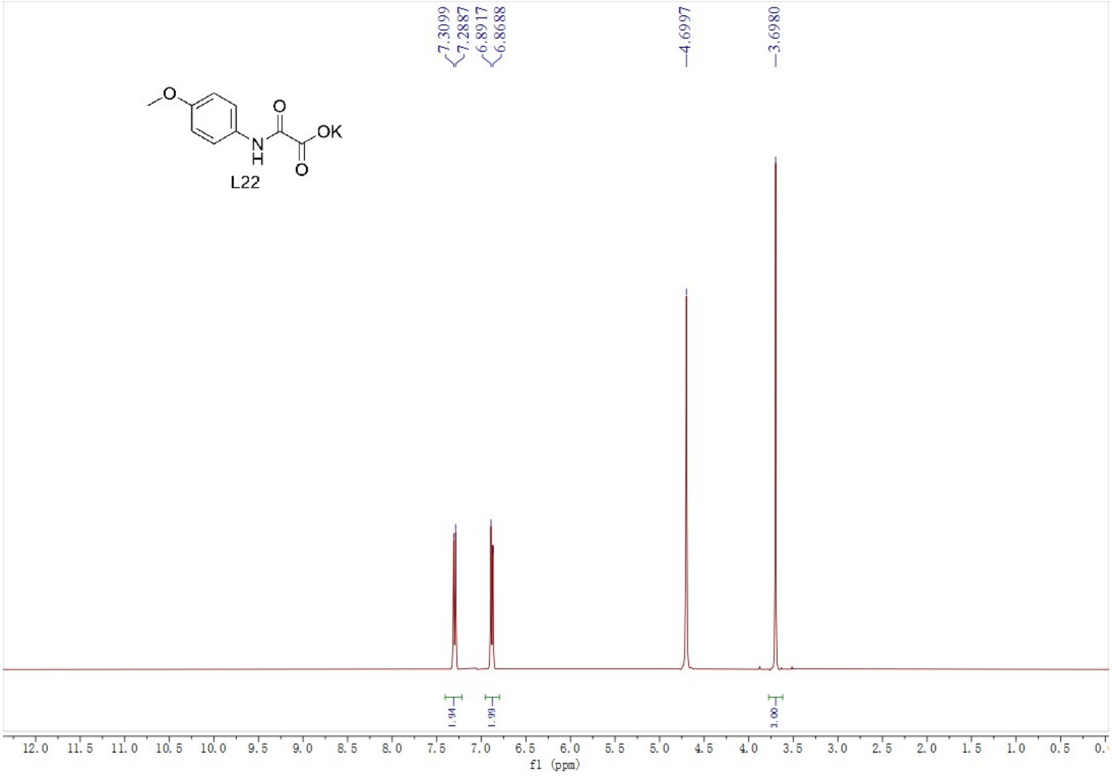
7.3099
7.2887
6.8917
6.8688
4.6997
3.6980
OK
N
H
O
O
L22
1.94
1.99
3.00
12.0
11.5
11.0
10.5
10.0
9.5
9.0
8.5
8.0
7.5
7.0
6.5
6.0
5.5
5.0
4.5
4.0
3.5
3.0
2.5
2.0
1.5
1.0
0.5
f1 (ppm)

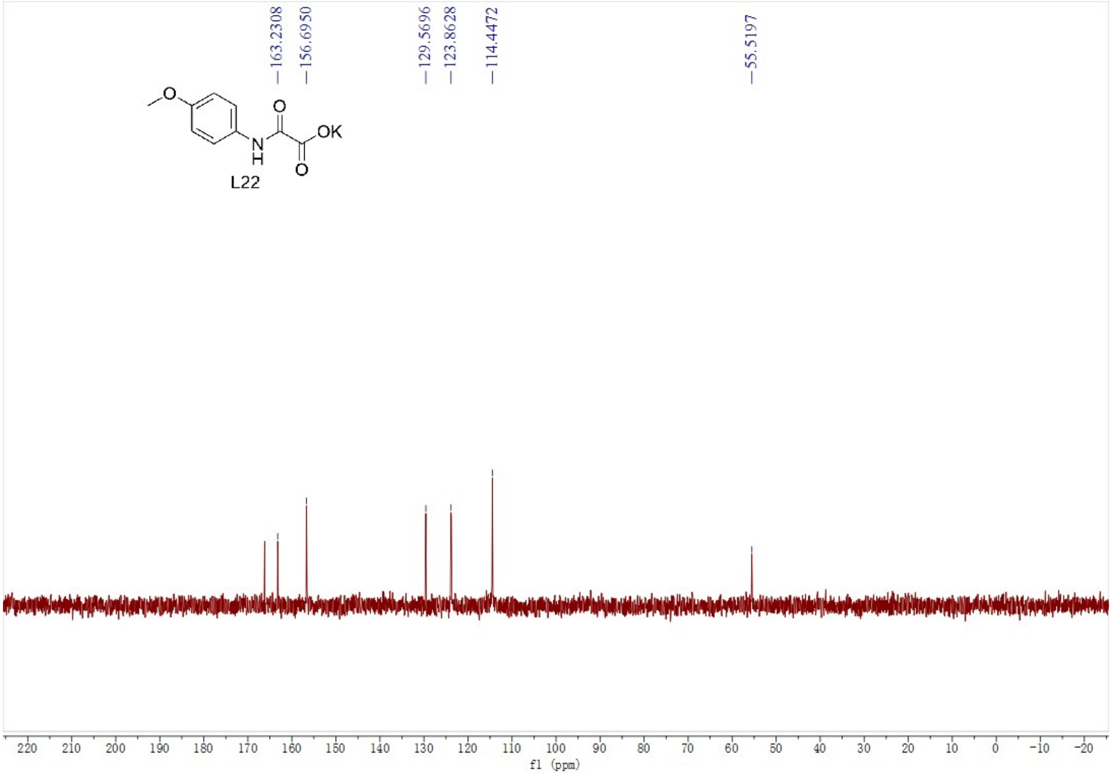
163.2308
156.6950
129.5696
123.8628
114.4472
55.5197
OK
N
H
O
O
L22
220
210
200
190
180
170
160
150
140
130
120
110
100
90
80
70
60
50
40
30
20
10
0
-10
-20
f1 (ppm)

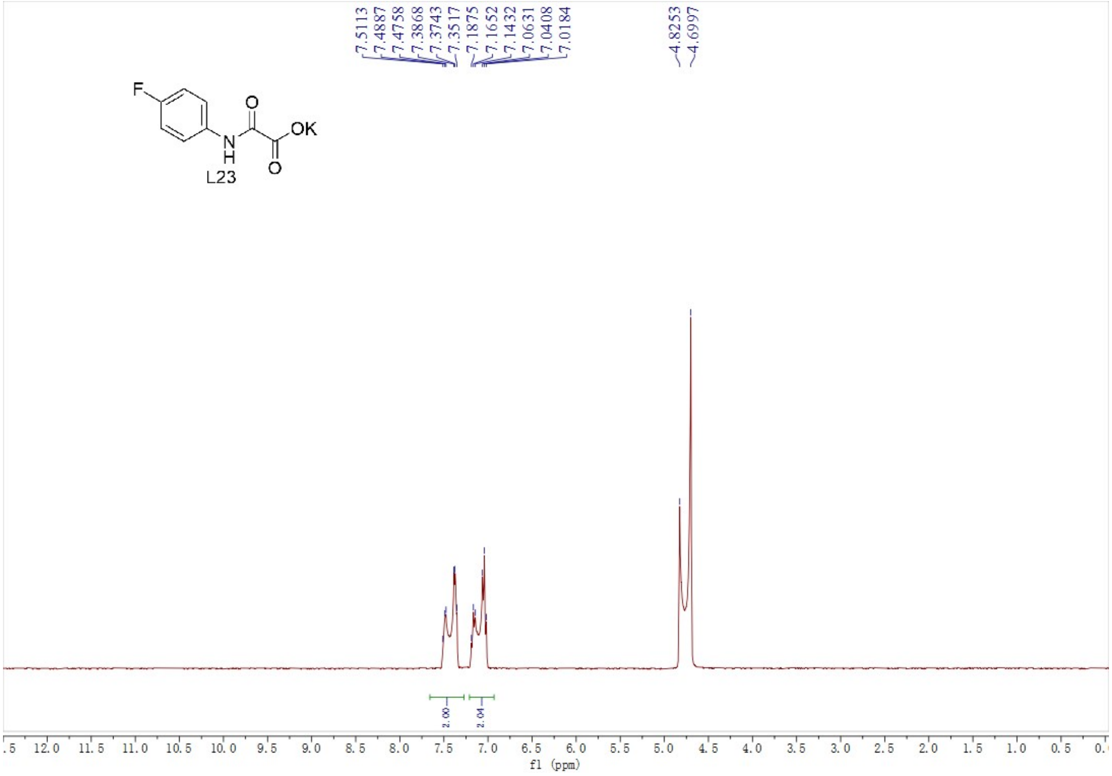
7.5113
7.4887
7.4758
7.3868
7.3743
7.3517
7.1875
7.1652
7.1432
7.0631
7.0408
7.0184
4.8253
4.6997
F
N
H
O
OK
O
L23
2.00
2.04
f1 (ppm)

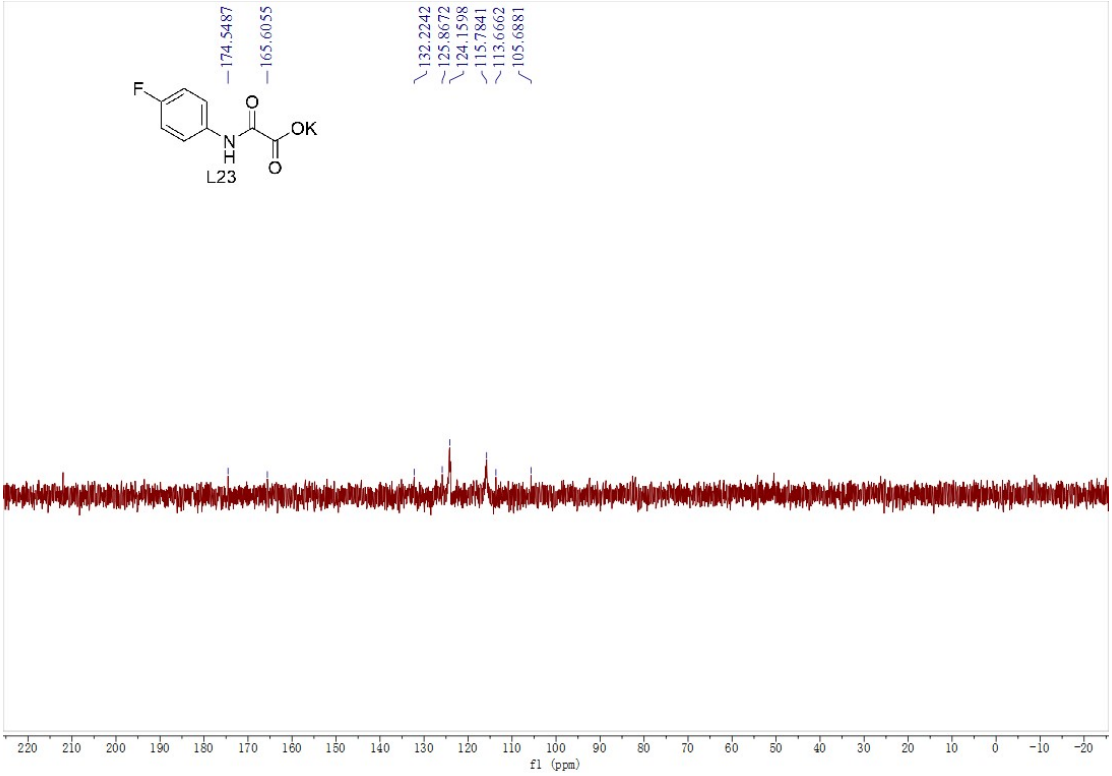
174.5487
165.6055
132.2242
125.8672
124.1598
115.7841
113.6662
105.6881
F
N
H
O
OK
O
L23
f1 (ppm)

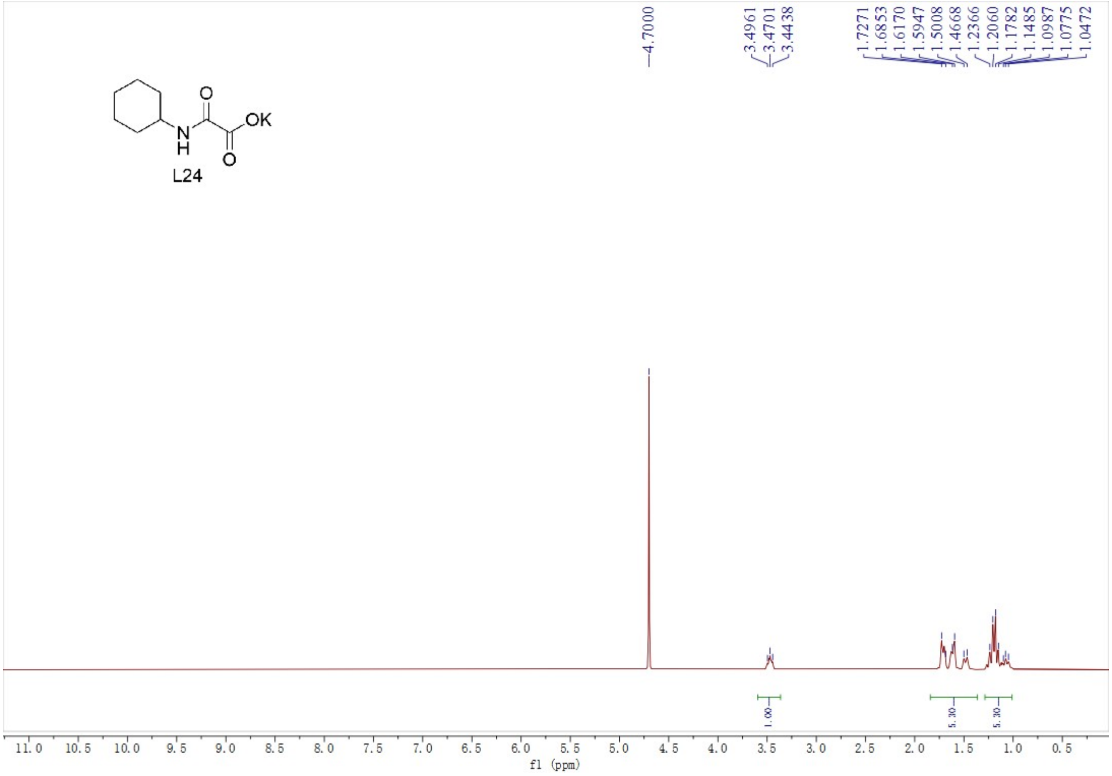
4.7000
3.4961
3.4701
3.4438
1.7271
1.6853
1.6170
1.5947
1.5008
1.4668
1.2366
1.2060
1.1782
1.1485
1.0987
1.0775
1.0472
OK
L24
1.00
5.30
5.30
11.0 10.5 10.0 9.5 9.0 8.5 8.0 7.5 7.0 6.5 6.0 5.5 5.0 4.5 4.0 3.5 3.0 2.5 2.0 1.5 1.0 0.5
f1 (ppm)

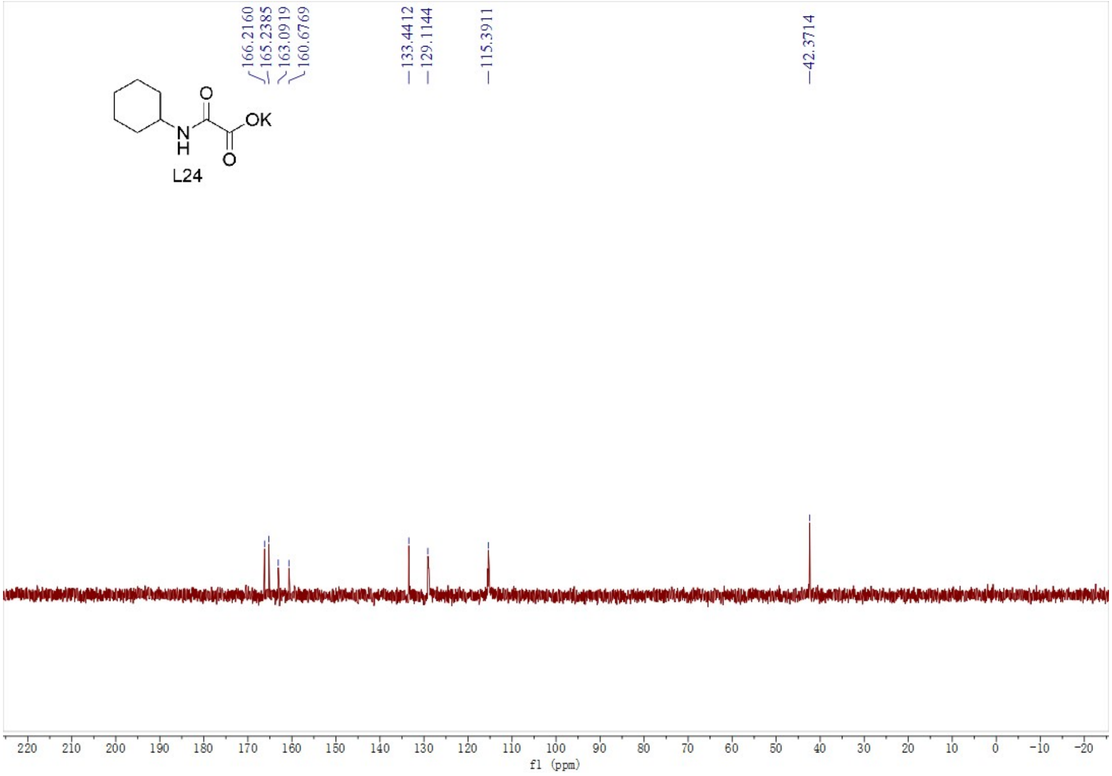
166.2160
165.2385
163.0919
160.6769
133.4412
129.1144
115.3911
42.3714
OK
L24
220 210 200 190 180 170 160 150 140 130 120 110 100 90 80 70 60 50 40 30 20 10 0 -10 -20
f1 (ppm)

…

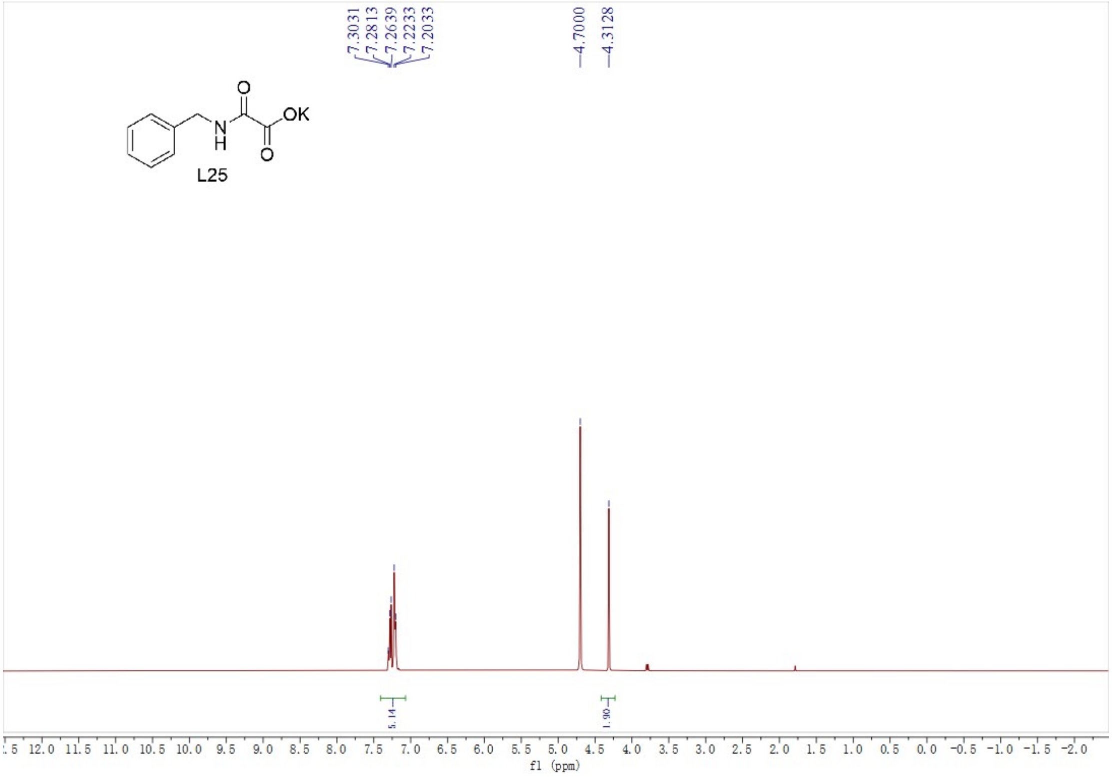
7.3031
7.2813
7.2639
7.2233
7.2033
4.7000
4.3128
OK
O
O
N
H
L25
5.14
1.90
12.0 11.5 11.0 10.5 10.0 9.5 9.0 8.5 8.0 7.5 7.0 6.5 6.0 5.5 5.0 4.5 4.0 3.5 3.0 2.5 2.0 1.5 1.0 0.5 0.0 -0.5 -1.0 -1.5 -2.0
f1 (ppm)

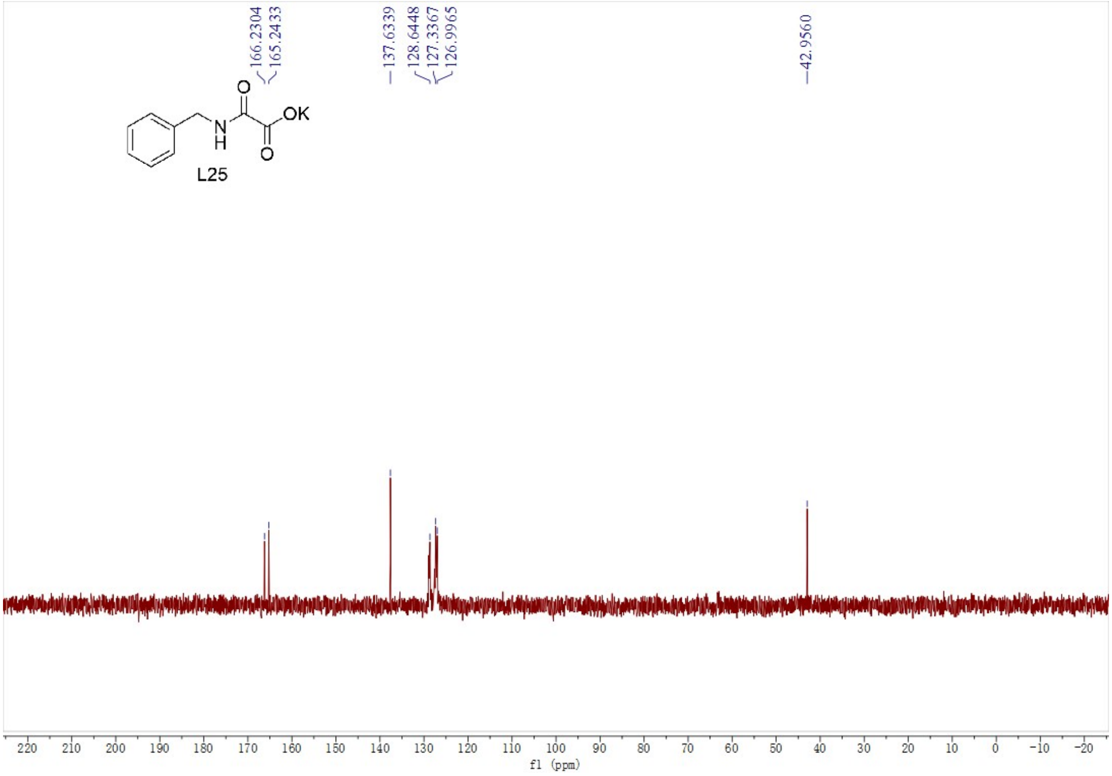
166.2304
165.2433
137.6339
128.6448
127.3367
126.9965
42.9560
OK
O
O
N
H
L25
220 210 200 190 180 170 160 150 140 130 120 110 100 90 80 70 60 50 40 30 20 10 0 -10 -20
f1 (ppm)

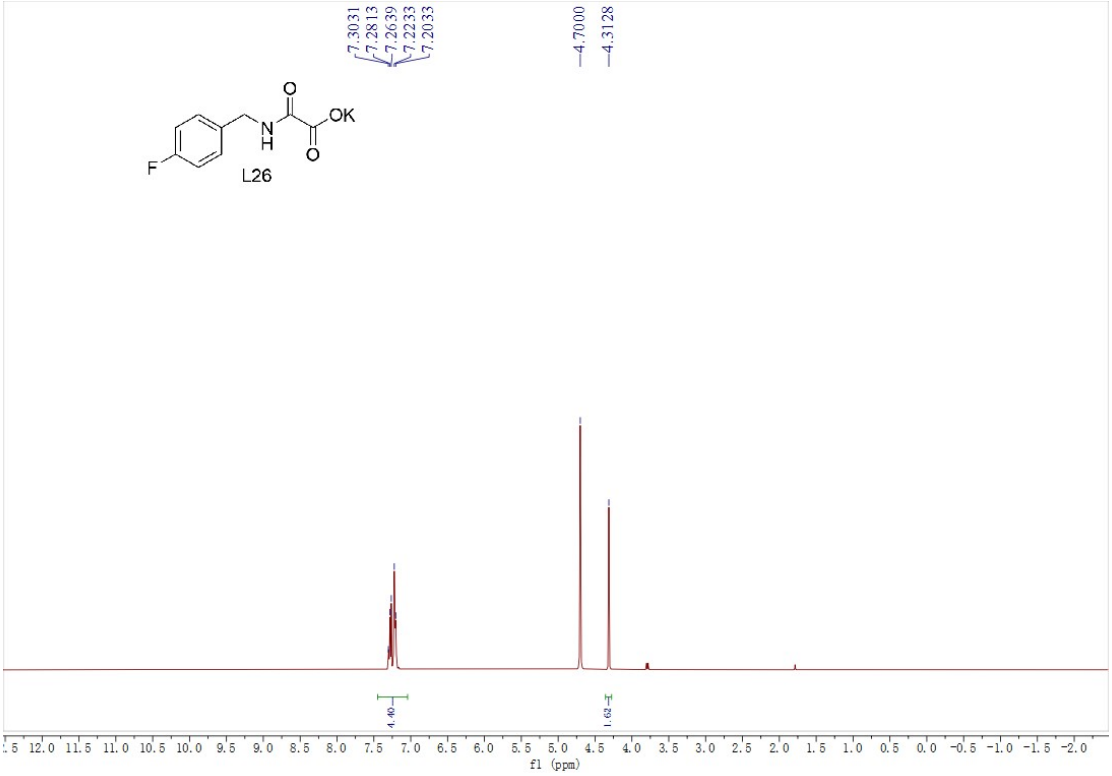
7.3031
7.2813
7.2639
7.2233
7.2033
4.7000
4.3128
OK
N
H
O
O
F
L26
4.40
1.62
f1 (ppm)

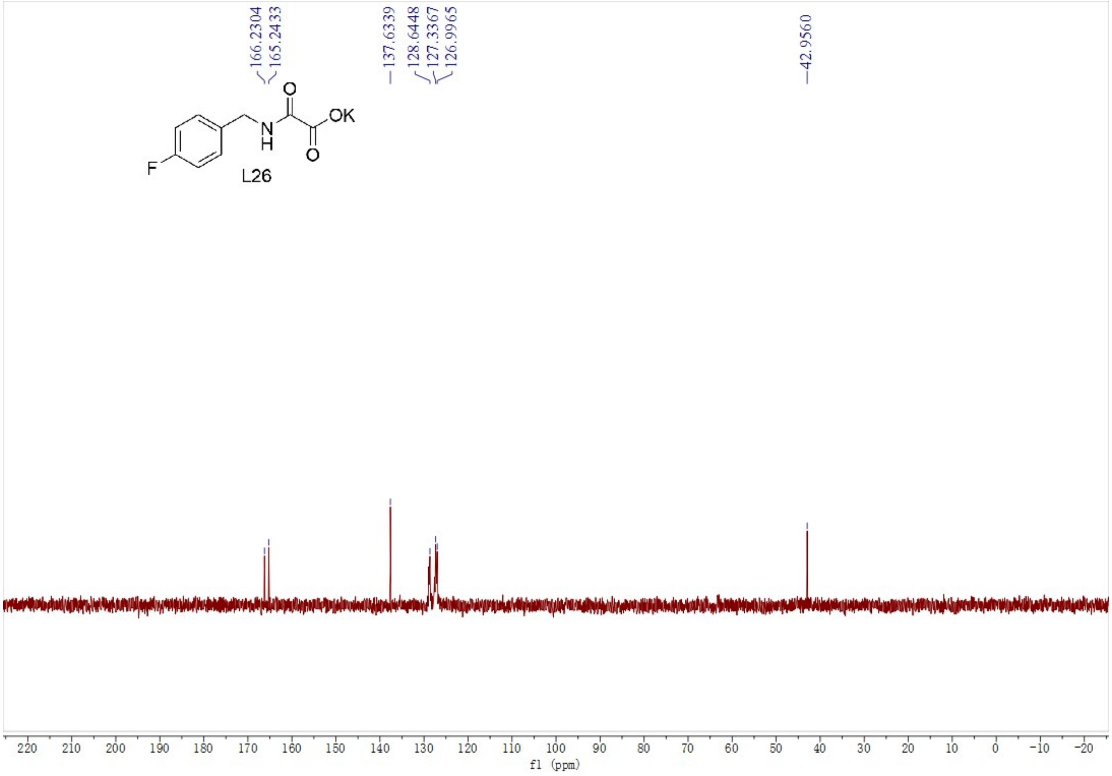
166.2304
165.2433
137.6339
128.6448
127.3367
126.9965
42.9560
OK
N
H
O
O
F
L26
f1 (ppm)

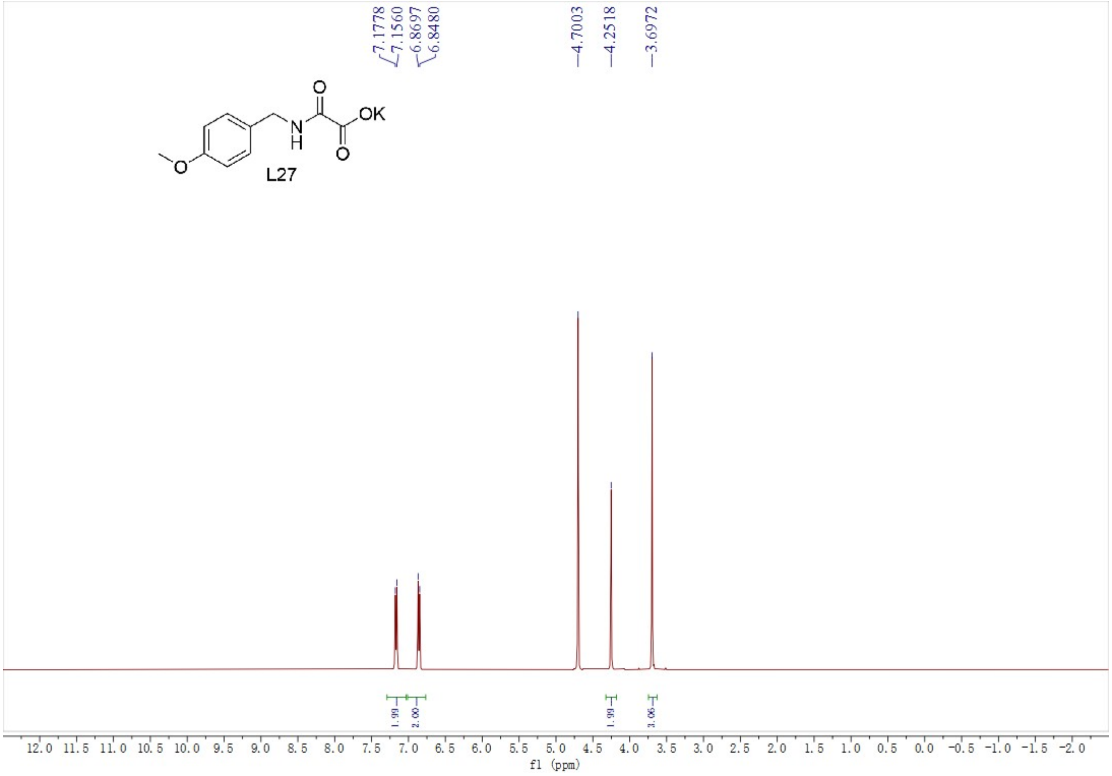
7.1778
7.1560
6.8697
6.8480
4.7003
4.2518
3.6972
OK
O
O
N
H
O
L27
1.99
2.00
1.99
3.06
12.0 11.5 11.0 10.5 10.0 9.5 9.0 8.5 8.0 7.5 7.0 6.5 6.0 5.5 5.0 4.5 4.0 3.5 3.0 2.5 2.0 1.5 1.0 0.5 0.0 -0.5 -1.0 -1.5 -2.0
f1 (ppm)

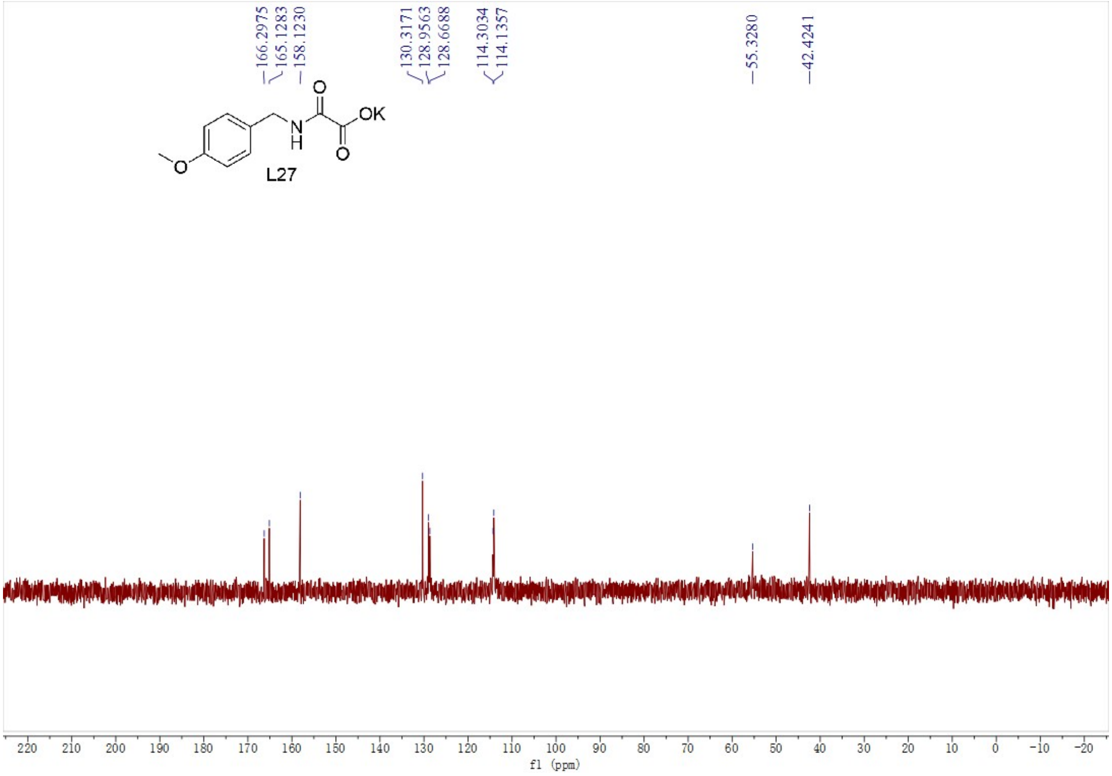
166.2975
165.1283
158.1230
130.3171
128.9563
128.6688
114.3034
114.1357
55.3280
42.4241
OK
O
O
N
H
O
L27
220 210 200 190 180 170 160 150 140 130 120 110 100 90 80 70 60 50 40 30 20 10 0 -10 -20
f1 (ppm)

…

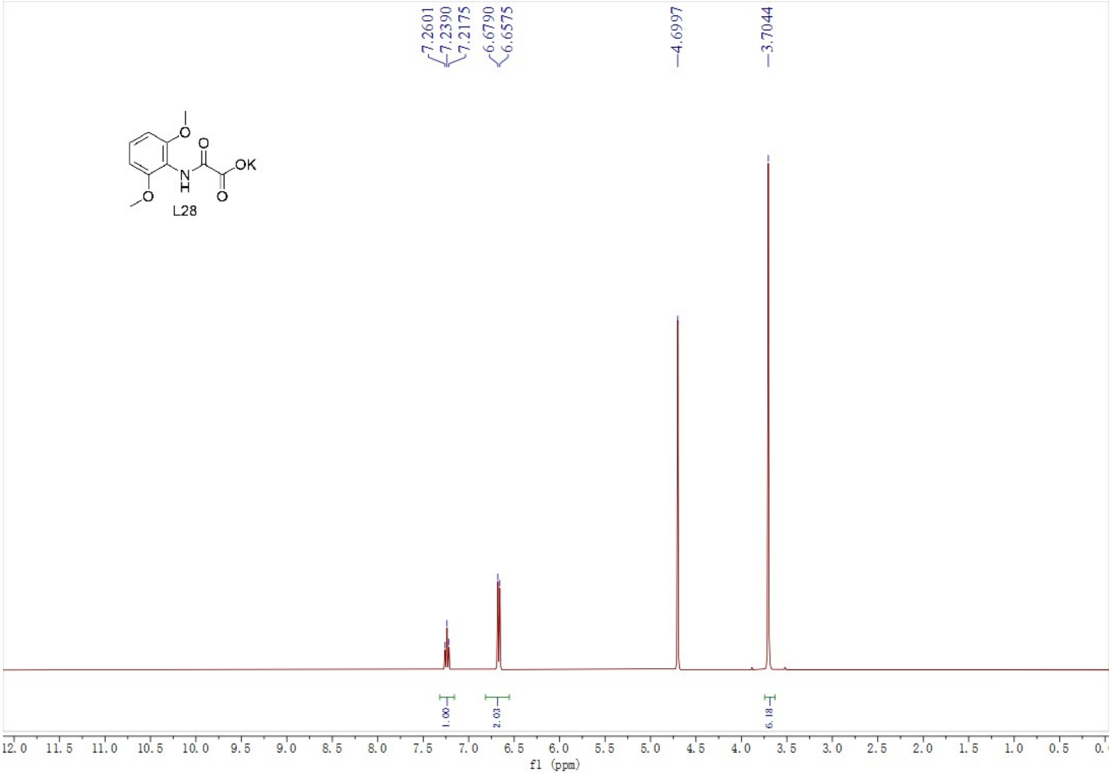
7.2601
7.2390
7.2175
6.6790
6.6575
4.6997
3.7044
OK
N
H
O
O
O
O
L28
1.00
2.03
6.18
12.0 11.5 11.0 10.5 10.0 9.5 9.0 8.5 8.0 7.5 7.0 6.5 6.0 5.5 5.0 4.5 4.0 3.5 3.0 2.5 2.0 1.5 1.0 0.5 0.0
f1 (ppm)

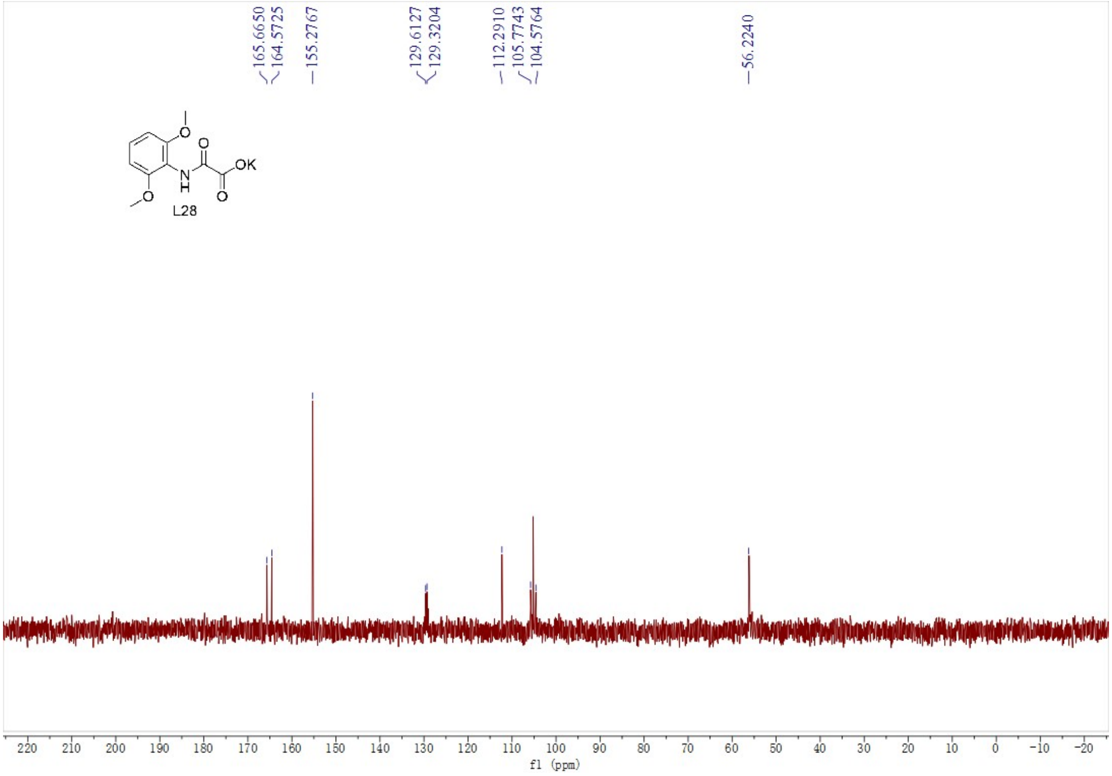
165.6650
164.5725
155.2767
129.6127
129.3204
112.2910
105.7743
104.5764
56.2240
OK
N
H
O
O
O
O
L28
220 210 200 190 180 170 160 150 140 130 120 110 100 90 80 70 60 50 40 30 20 10 0 −10 −20
f1 (ppm)

…

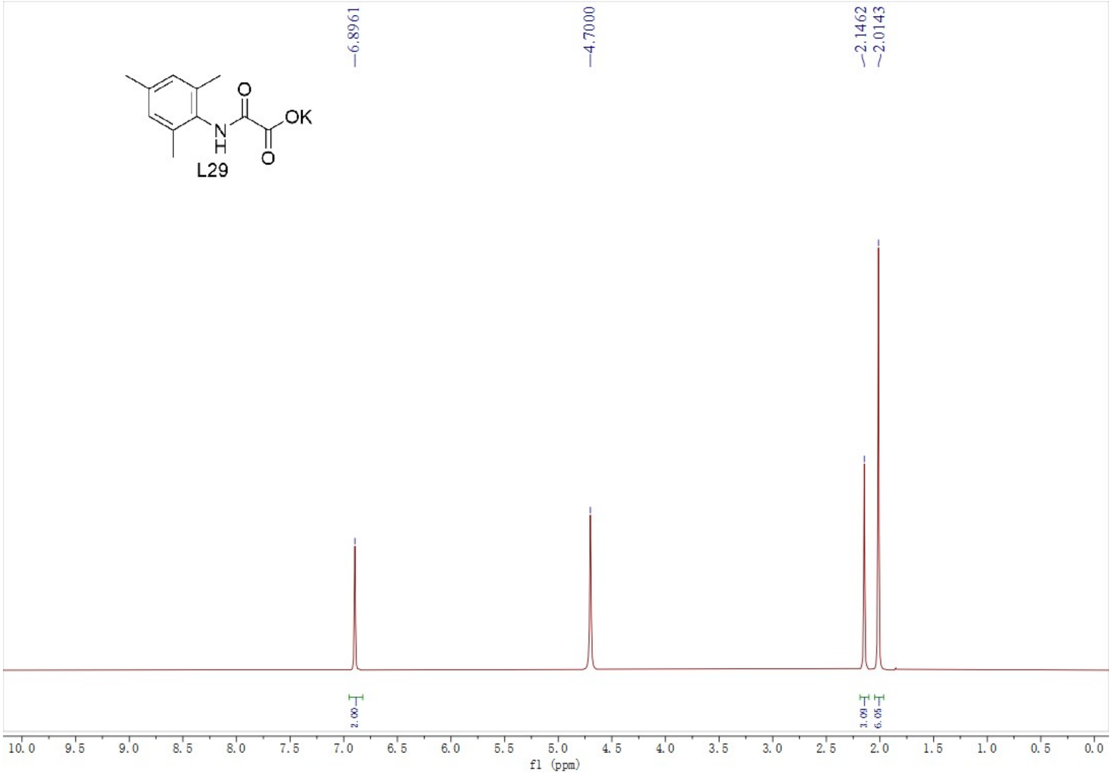
6.8961
4.7000
2.1462
2.0143
L29
OK
2.00
3.09
6.05
f1 (ppm)

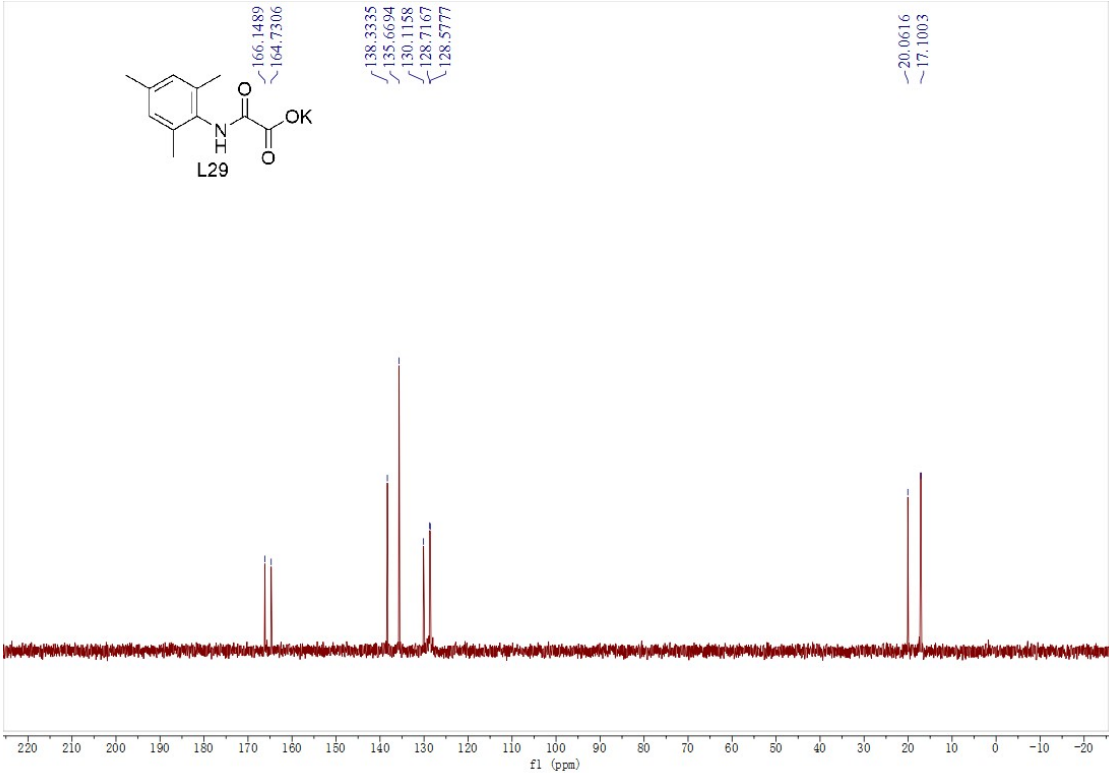
166.1489
164.7306
138.3335
135.6694
130.1158
128.7167
128.5777
20.0616
17.1003
L29
OK
f1 (ppm)

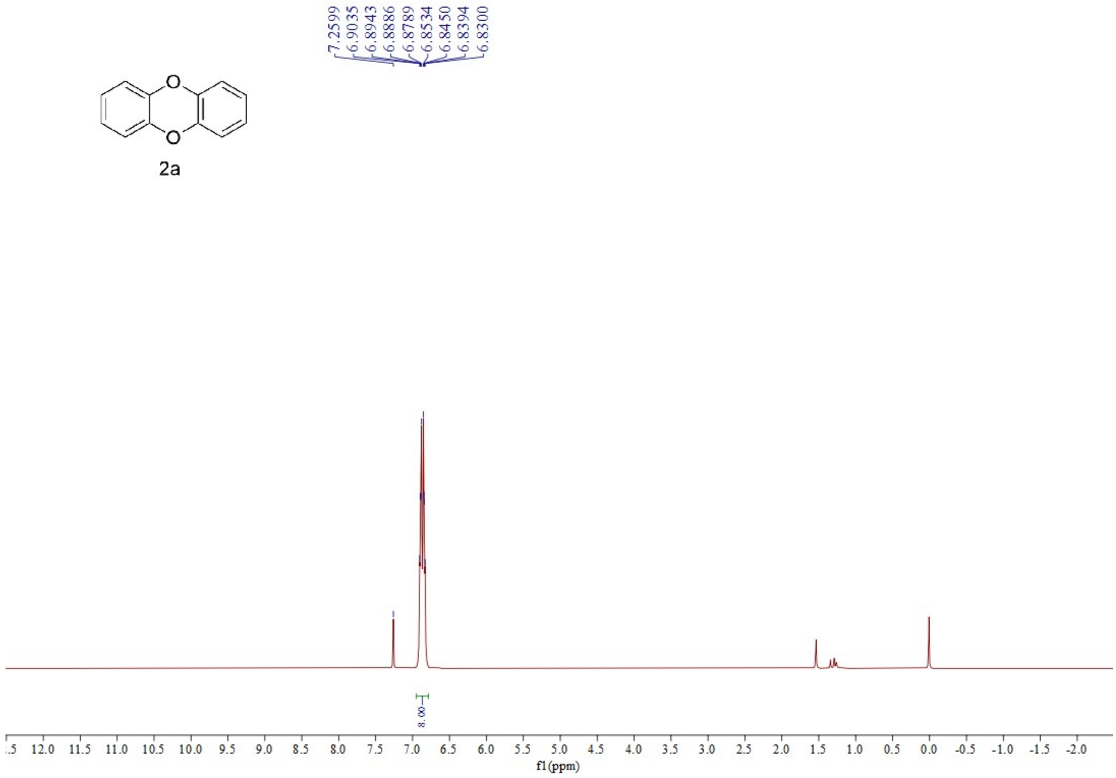
2a
7.2599
6.9035
6.8943
6.8886
6.8789
6.8534
6.8450
6.8394
6.8300
8.00
.5 12.0 11.5 11.0 10.5 10.0 9.5 9.0 8.5 8.0 7.5 7.0 6.5 6.0 5.5 5.0 4.5 4.0 3.5 3.0 2.5 2.0 1.5 1.0 0.5 0.0 -0.5 -1.0 -1.5 -2.0
f1 (ppm)

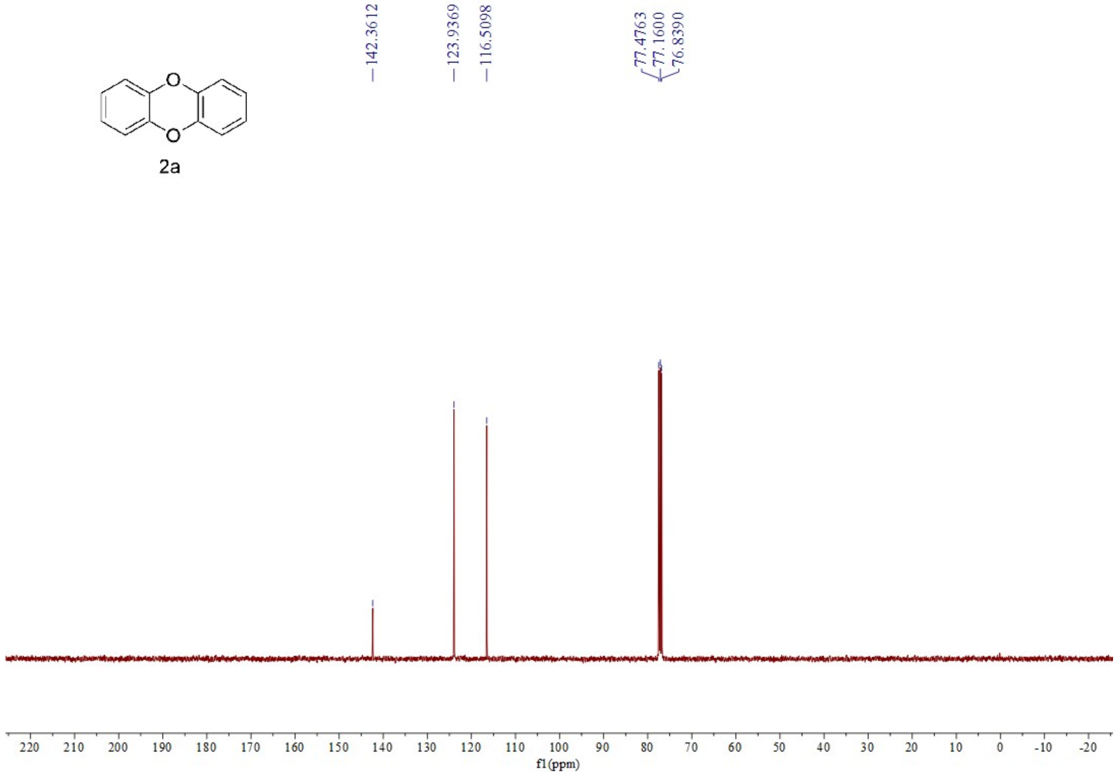
2a
142.3612
123.9369
116.5098
77.4763
77.1600
76.8390
220 210 200 190 180 170 160 150 140 130 120 110 100 90 80 70 60 50 40 30 20 10 0 -10 -20
f1 (ppm)

…

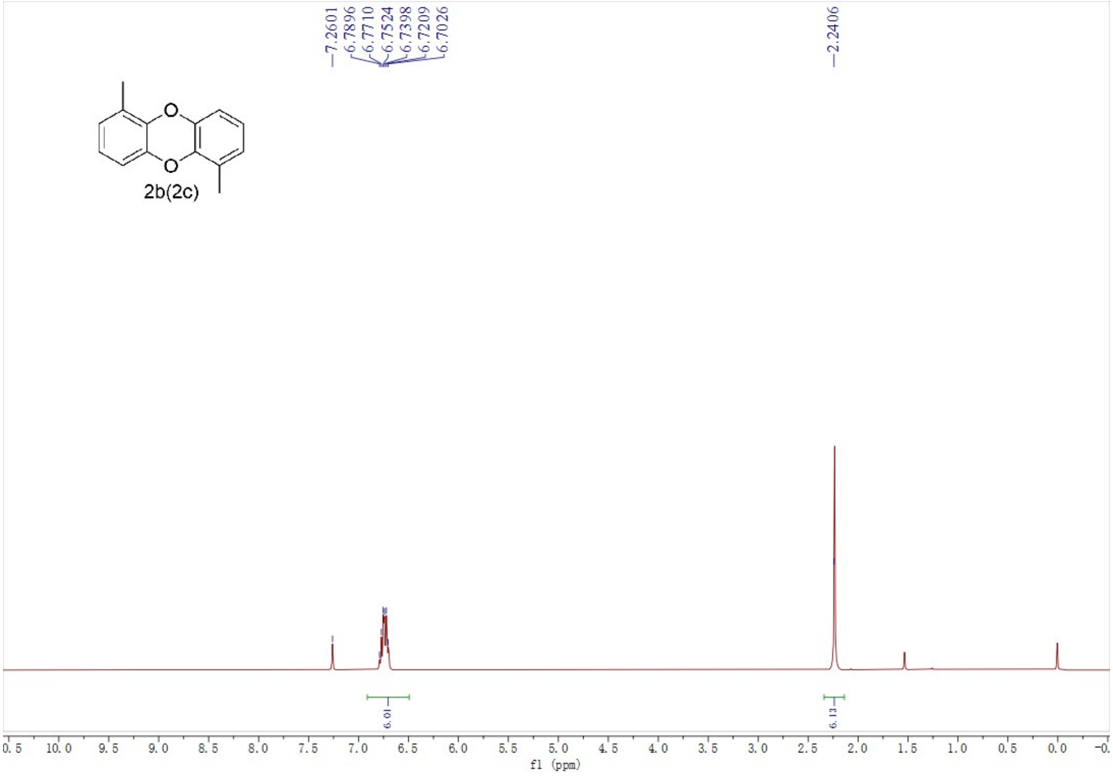

7.2601
6.7896
6.7710
6.7524
6.7398
6.7209
6.7026
2.2406
2b(2c)
6.01
6.13
fl (ppm)

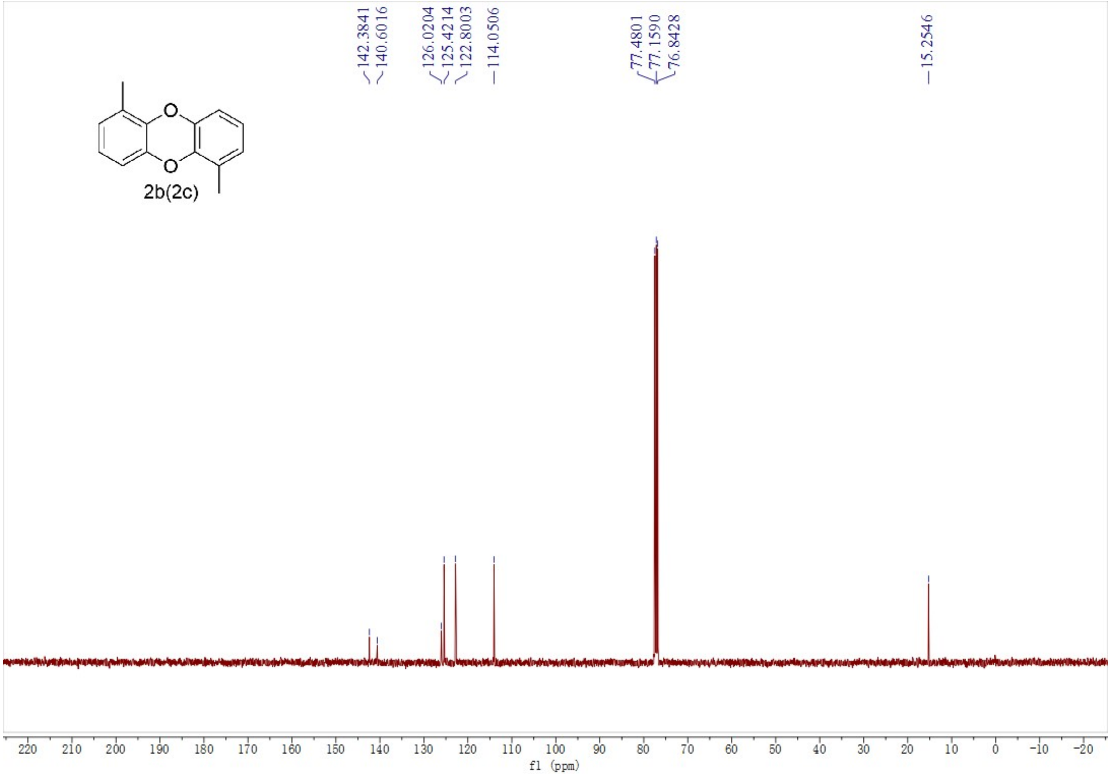

142.3841
140.6016
126.0204
125.4214
122.8003
114.0506
77.4801
77.1590
76.8428
15.2546
2b(2c)
fl (ppm)

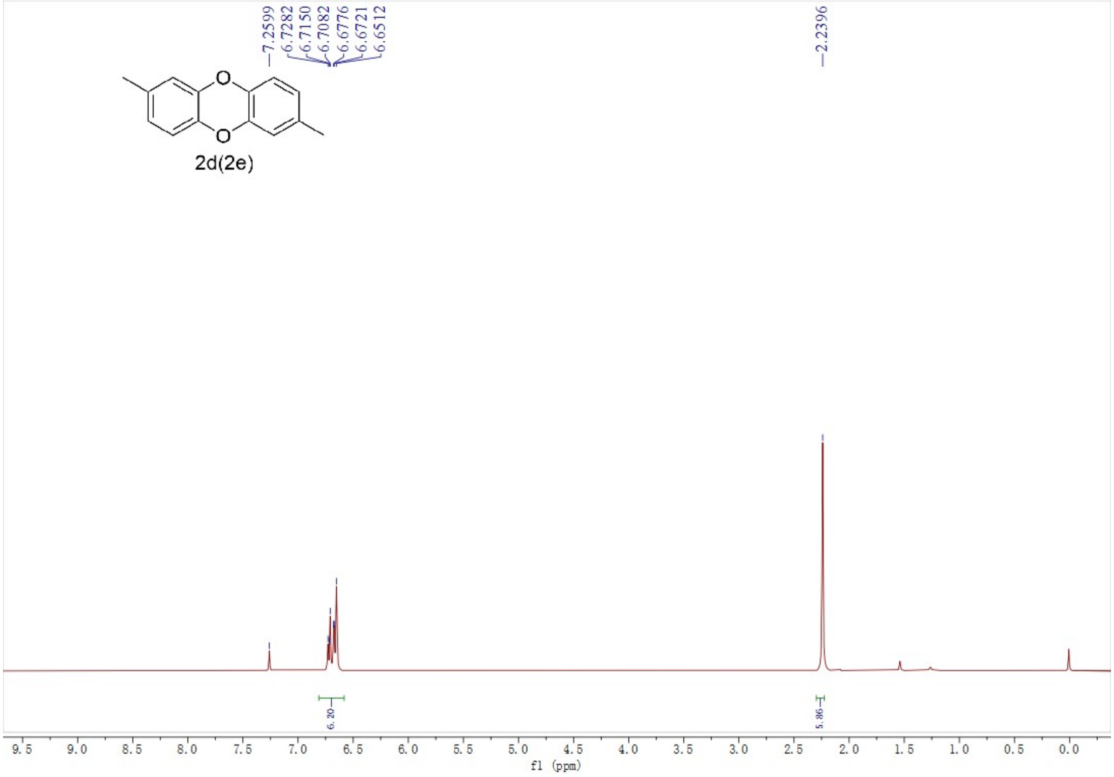
7.2599
6.7282
6.7150
6.7082
6.6776
6.6721
6.6512
2.2396
2d(2e)
6.20
5.86
9.5 9.0 8.5 8.0 7.5 7.0 6.5 6.0 5.5 5.0 4.5 4.0 3.5 3.0 2.5 2.0 1.5 1.0 0.5 0.0
f1 (ppm)

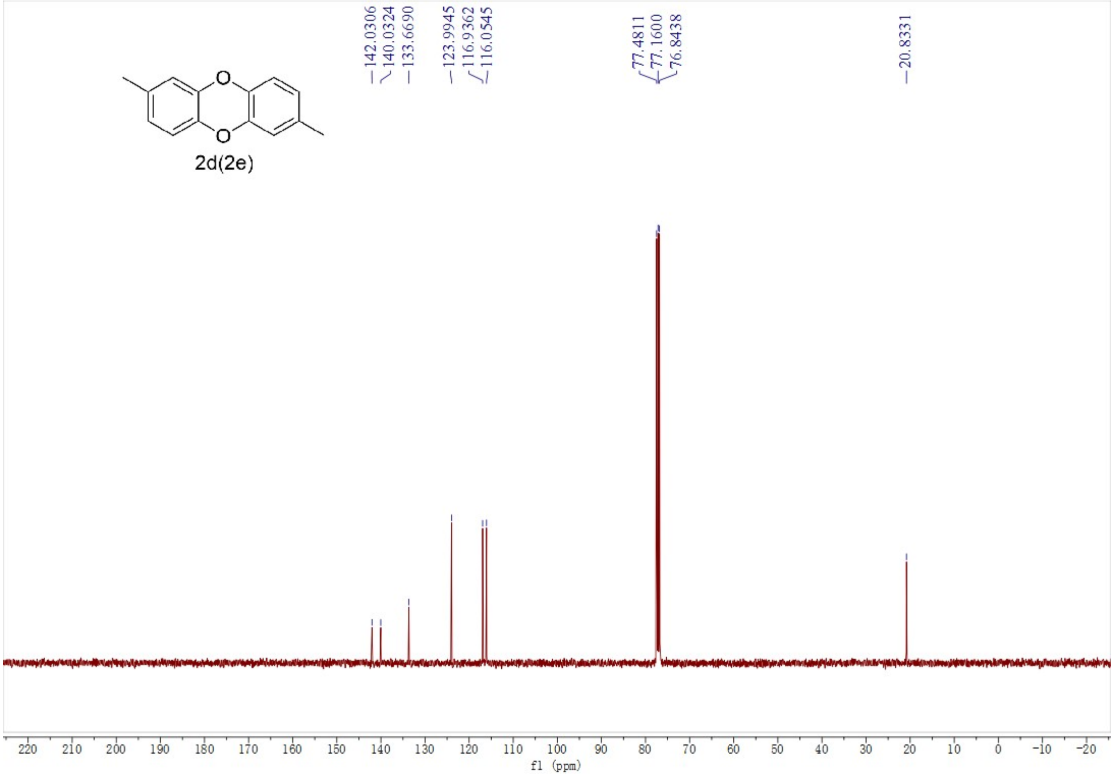
142.0306
140.0324
133.6690
123.9945
116.9362
116.0545
77.4811
77.1600
76.8438
20.8331
2d(2e)
220 210 200 190 180 170 160 150 140 130 120 110 100 90 80 70 60 50 40 30 20 10 0 -10 -20
f1 (ppm)

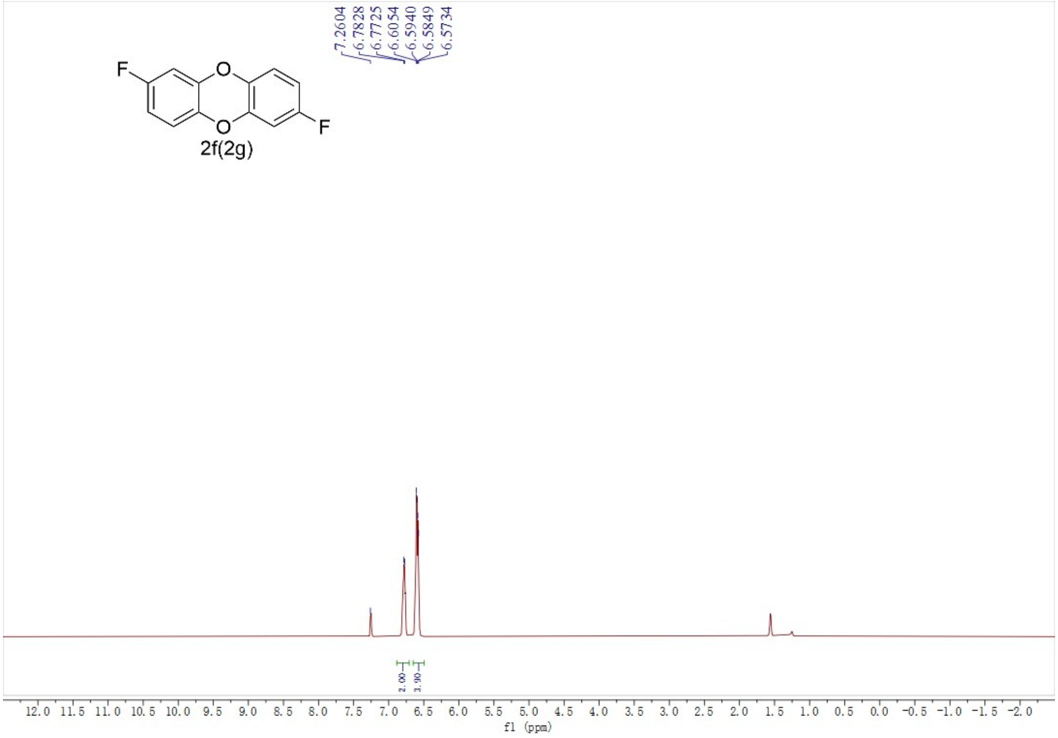
7.2604
6.7828
6.7725
6.6054
6.5940
6.5849
6.5734
F
O
O
F
2f(2g)
2.00
3.90
12.0 11.5 11.0 10.5 10.0 9.5 9.0 8.5 8.0 7.5 7.0 6.5 6.0 5.5 5.0 4.5 4.0 3.5 3.0 2.5 2.0 1.5 1.0 0.5 0.0 -0.5 -1.0 -1.5 -2.0
f1 (ppm)

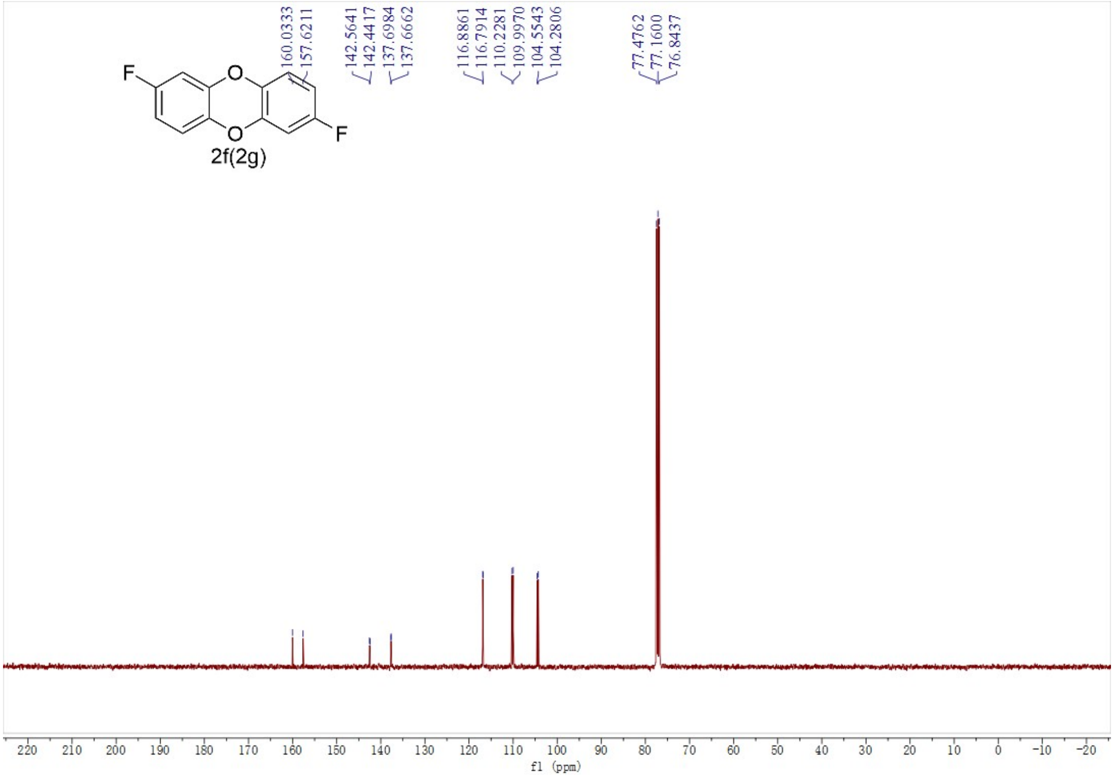
160.0333
157.6211
142.5641
142.4417
137.6984
137.6662
116.8861
116.7914
110.2281
109.9970
104.5543
104.2806
77.4762
77.1600
76.8437
F
O
O
F
2f(2g)
220 210 200 190 180 170 160 150 140 130 120 110 100 90 80 70 60 50 40 30 20 10 0 -10 -20
f1 (ppm)

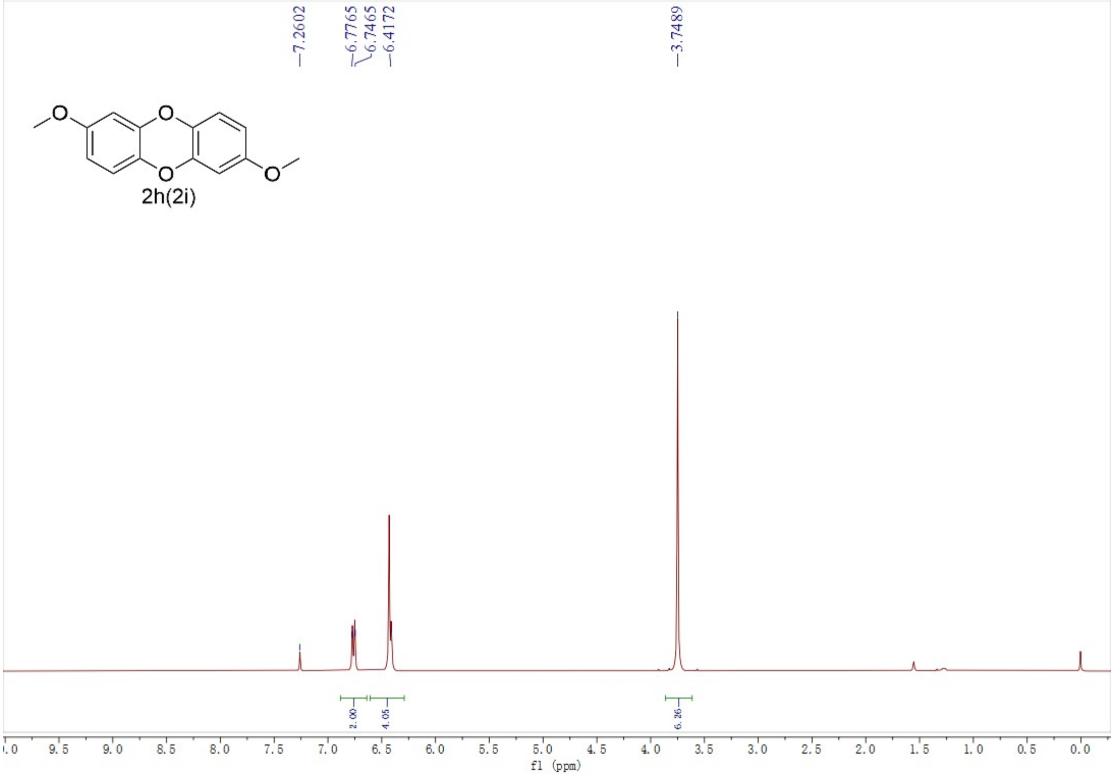
7.2602
6.7765
6.7465
6.4172
3.7489
2h(2i)
2.00
4.05
6.26
9.5 9.0 8.5 8.0 7.5 7.0 6.5 6.0 5.5 5.0 4.5 4.0 3.5 3.0 2.5 2.0 1.5 1.0 0.5 0.0
f1 (ppm)

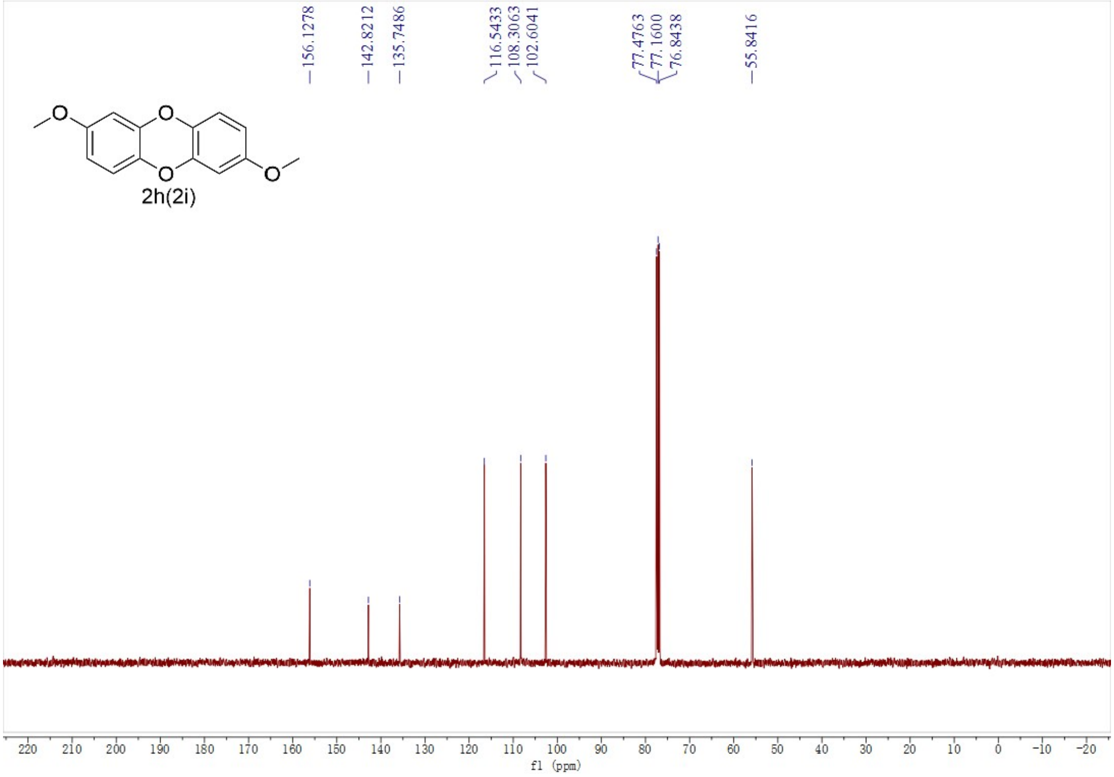
156.1278
142.8212
135.7486
116.5433
108.3063
102.6041
77.4763
77.1600
76.8438
55.8416
2h(2i)
220 210 200 190 180 170 160 150 140 130 120 110 100 90 80 70 60 50 40 30 20 10 0 -10 -20
f1 (ppm)

…

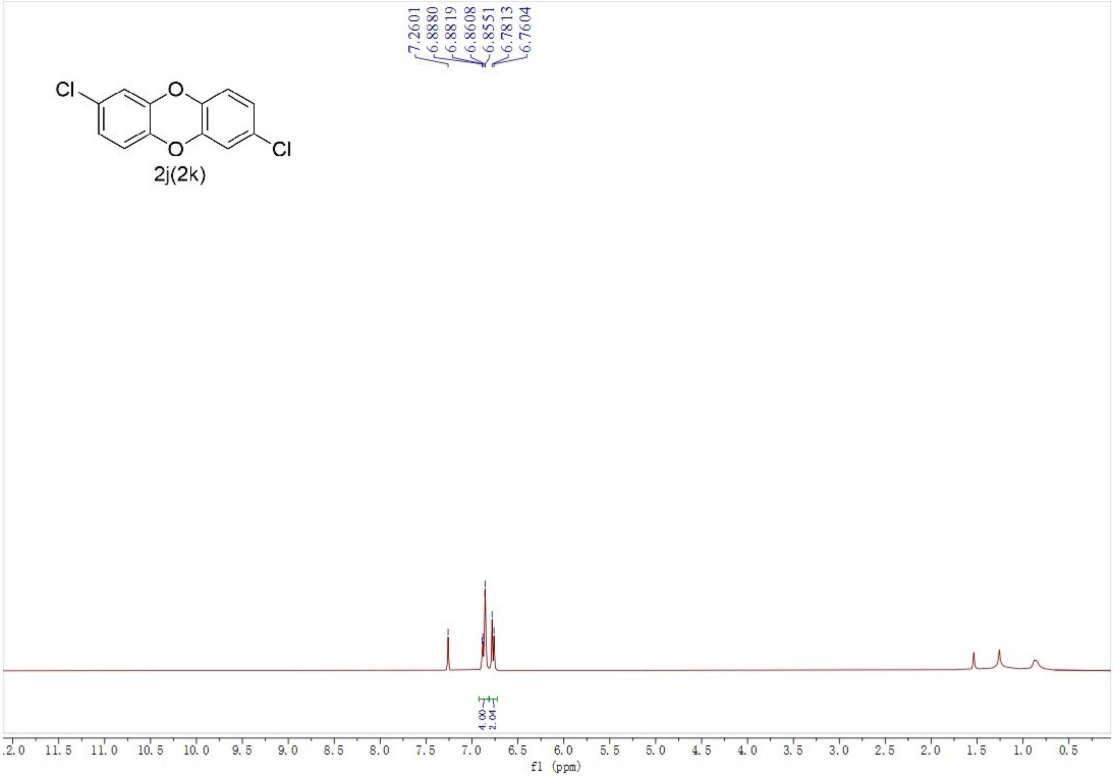
7.2601
6.8880
6.8819
6.8608
6.8551
6.7813
6.7604
Cl
O
O
Cl
2j(2k)
4.00
2.04
12.0 11.5 11.0 10.5 10.0 9.5 9.0 8.5 8.0 7.5 7.0 6.5 6.0 5.5 5.0 4.5 4.0 3.5 3.0 2.5 2.0 1.5 1.0 0.5
f1 (ppm)

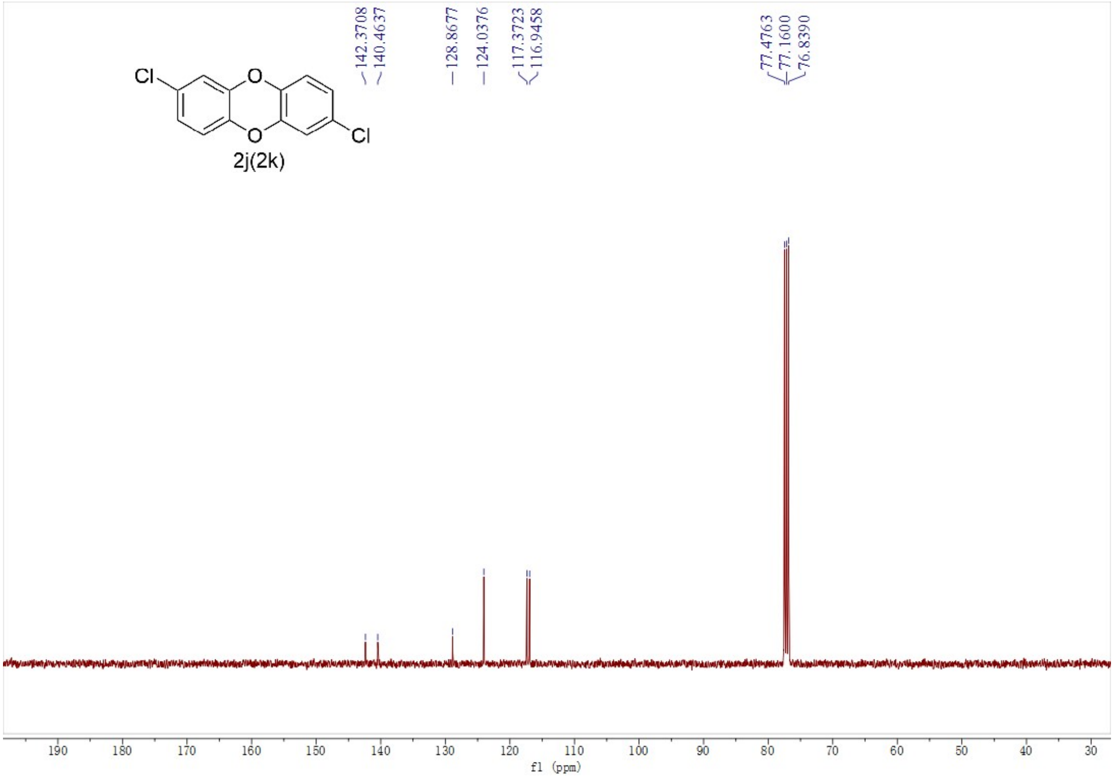
142.3708
140.4637
128.8677
124.0376
117.3723
116.9458
77.4763
77.1600
76.8390
Cl
O
O
Cl
2j(2k)
190 180 170 160 150 140 130 120 110 100 90 80 70 60 50 40 30
f1 (ppm)

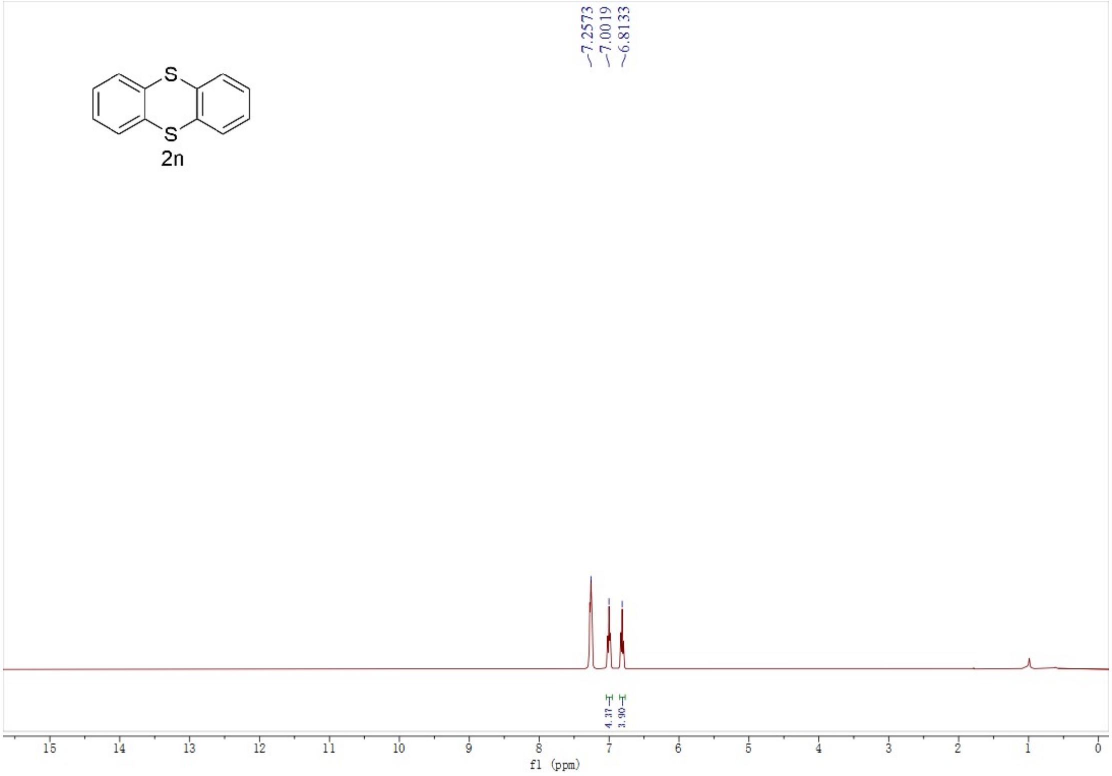
7.2573
7.0019
6.8133
S
S
2n
4.17
3.90
15 14 13 12 11 10 9 8 7 6 5 4 3 2 1 0
f1 (ppm)

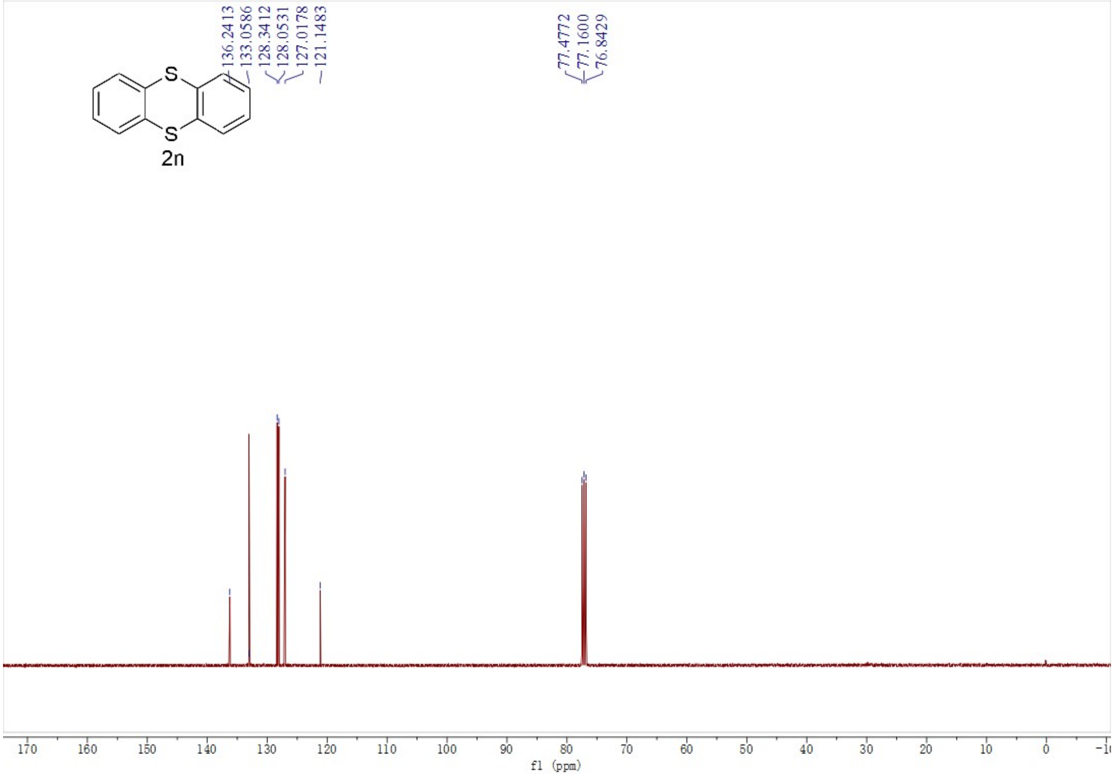
136.2413
133.0586
128.3412
128.0531
127.0178
121.1483
77.4772
77.1600
76.8429
S
S
2n
170 160 150 140 130 120 110 100 90 80 70 60 50 40 30 20 10 0
f1 (ppm)
